# High Expression of UPK3A Promotes the Progression of Gastric Cancer Cells by Inactivating p53 Pathway

**DOI:** 10.1155/2022/6897561

**Published:** 2022-06-21

**Authors:** Deliang Xu, Jing Guo, Hongwei Xu

**Affiliations:** ^1^Department of Gastroenterology, Zaozhuang Municipal Hospital, Zaozhuang, Shandong 277100, China; ^2^Department of Gastroenterology, Shandong Provincial Hospital, Jinan, Shandong 250000, China

## Abstract

**Background:**

Gastric cancer is a common gastrointestinal tract cancer and is a considerable health burden worldwide. TCGA analysis found Uroplakin 3A (UPK3A) was upregulated in gastric cancer tissues. Our study was designed to investigate the underlying mechanism of Uroplakin 3A (UPK3A) in gastric cancer.

**Methods:**

Data from TCGA database were used to assess the expression, and Kaplan-Meier plotter analysis was used to assess the prognosis value of UPK3A. Furthermore, there are effects of UPK3A silencing on the activity, proliferation, migration, and invasion of human gastric cancer cells (SNU-216 and HGC-27) using 3-(4,5-dimethylthiazol-2-yl)-2,5-diphenyltetrazolium bromide (MTT), colony formation, wound healing, and Transwell assays. In addition, the expression of UPK3A, p53, KLF4, ZMAT3, MDM2, and SP1 was detected by qRT-PCR and Western blot assay.

**Results:**

UPK3A was markedly upregulated in gastric cancer tissues compared to that in normal tissues, and patients with high UPK3A level showed poor prognosis. UPK3A was highly expressed in human gastric cancer cell lines compared to that in a normal human gastric epithelial cell line. Silencing of UPK3A inhibited the proliferation, migration, and invasion of gastric cancer cells. Kyoto Encyclopedia of Genes and Genomes (KEGG) pathway enrichment analysis revealed that UPK3A was involved in the p53 signaling pathway. UPK3A suppressed the activation of p53 signaling pathway, and treatment with Pifithrin-*α* (an inhibitor of the p53 signaling pathway) or silencing of p53 significantly reversed the effect of UPK3A silencing on the expression of p53, KLF4, ZMAT3, MDM2, and SP1.

**Conclusion:**

Our findings showed that UPK3A promotes the progression of gastric cancer by regulating the p53 signaling pathway and could be a potential therapeutic target for gastric cancer.

## 1. Introduction

Gastric cancer is one of the most common malignant cancers worldwide. The “Global Cancer Statistics 2018” report indicated that there were over one million new cases of gastric cancer worldwide in 2018 and ranked gastric cancer as the 5^th^ most prevalent cancer [1]. Traditional treatments for gastric cancer include surgery, chemotherapy, and radiotherapy [2, 3]. Although available treatments have been greatly improved in recent years, their effectiveness against advanced gastric cancer remains limited, as the 5-year survival rate still remains at 30%-35% [4]. Molecular-targeted therapy has recently emerged as an effective treatment for tumors in the circulatory system and solid tumors and has opened up new avenues for tumor prevention and treatment strategies [5–8].

Uroplakin 3A (UPK3A) was originally isolated from a human asymmetric unit membrane. The ~1.7 kb UPK3A transcript encodes a protein with a molecular weight of 28.9 kD [9]. A previous study reported that urine UPK3A level is higher in patients with bladder cancer, and UPK3A in the urine is regarded as a sensitive biomarker for the detection of bladder cancer [10]. However, the role of UPK3A in the progression of gastric cancer remains unclear.

p53 is an important tumor suppressor gene, and p53 mutations are often responsible for the occurrence of many malignant tumors [11, 12]. The expression of mutant p53 is closely related to the occurrence, development, degree of differentiation, depth of invasion, metastasis, and prognosis of gastrointestinal tumors [13, 14]. The study of Wang et al. indicated that activation of the p53 signaling pathway sensitizes colorectal cancer cells to chemotherapy and inhibits tumor growth [14]. Additionally, the research of Choi et al. showed that silencing of p53 inhibits the apoptosis of gastric cancer cells and the expression of cleaved caspase-3 protein [15]. However, whether UPK3A can regulate the p53 signaling pathway and affect the progression of gastric cancer requires further investigation.

In this study, we demonstrate that UPK3A plays an oncogenic role in the progression of gastric cancer *in vitro*. We further explored the mechanism of UPK3A promoting the progression of gastric cancer. Our findings indicate that UPK3A may be a potential target for the treatment of gastric cancer.

## 2. Methods

### 2.1. Bioinformatic Analysis

UPK3A expressions in gastric cancer cases and normal samples were analyzed based on TCGA database. Survival analysis was performed using the Kaplan–Meier curve method. Kyoto Encyclopedia of Genes and Genomes (KEGG) pathway enrichment analysis was performed to analyze the pathways and target genes of UPK3A.

### 2.2. Gene Set Enrichment Analysis (GSEA)

For GSEA, UPK3A expression was treated as a numeric variable. The Pearson correlation coefficient of other genes and UPK3A expression was calculated, and then the genes were sequenced according to the correlation coefficient. Using the hallmark gene sets deposited in the GSEA Molecular Signatures Database resource (h.all.v7.1.symbols.gmt, https://www.gsea-msigdb.org/gsea/index.jsp), we identified the differential pathways between the high-UPK3A expression and low-UPK3A expression specimens. The number of permutations was 1000. NES (normalized enrichment score) > 1 and FDR (false discovery rate) *q* val < 0.05 were set as cut-offs for significant enrichment.

### 2.3. Cell Culture and Transfection

Human gastric cancer cell lines SNU-216 (harboring mutant p53), AGS (harboring mutant p53), and HGC-27 (harboring mutant p53) and the GES-1 human gastric epithelial cell line were obtained from COBIOER (Nanjing, China). Cell lines were cultured in RPMI-1640 medium (Hyclone, Logan, USA) supplemented with 10% FBS.

The UPK3A-specific siRNA, p53-specific siRNA, and negative control siRNA were purchased from RiboBio Co. Ltd. (Guangzhou, China). Cells were assigned to the following groups: Mock (untreated SNU-216 and HGC-27 cells), si-NC (SNU-216 and HGC-27 cells transfected with siRNA negative control), si-UPK3A#1 (SNU-216 and HGC-27 cells transfected with siRNA-UPK3A#1), si-UPK3A#2 (SNU-216 and HGC-27 cells transfected with siRNA-UPK3A#2), si-p53#1 (SNU-216 and HGC-27 cells transfected with siRNA-p53#1), si-p53#2 (SNU-216 and HGC-27 cells transfected with siRNA-p53#2), si-UPK3A#2+si-p53#1 (SNU-216 and HGC-27 cells co-transfected with iRNA-UPK3A#2 and siRNA-p53#1), and si-UPK3A#2+Pifithrin-*α* (SNU-216 and HGC-27 cells pre-treated with Pifithrin-*α* (p53 inhibitor) and transfected with siRNA-UPK3A#2). Transfection was performed using Lipofectamine 2000 (Invitrogen, Waltham, USA).

### 2.4. MTT Assay

The viability of gastric cancer cells was determined by MTT assay. A total of 10^4^ cells/well were inoculated into 96-well plates, with three replicates per group. Cell viability was detected after incubation for 0, 24, 48, and 72 hrs. Subsequently, 20 *μ*l MTT solution (5 mg/ml) was added to each well for 4 hrs at 37°C. Dimethyl sulfoxide (150 *μ*l) was added to each well, plates were gently agitated, and the absorbance at 490 nm was determined using a microplate reader. Each experiment was repeated thrice.

### 2.5. Colony Formation Assay

Following 48 hrs of transfection, cells were trypsinized and seeded in a 35 mm petri dish at a density of 500 cells/dish. The cell culture dishes were incubated for 1-2 weeks, and 1 ml fresh culture medium was added into the dishes every 2-3 days. When the cell clones became visible, the culture solution was removed, and cells were washed twice with PBS. After fixing with 4% paraformaldehyde for 30 minutes and staining with 0.1% crystal violet for 30 minutes, the number of cell clones was counted. Each experiment was repeated three times.

### 2.6. Wound Healing Assay

After transfection, the cells were trypsinized, seeded in a 6-well plate at a density of 5 × 10^5^ cells/well, and cultured at 37°C for 12 hrs. A wound was inflicted in the middle of well using a sterilized tip. The cells were then washed with PBS and photographed. After 24 hrs of incubation, the wound was photographed again. The Image J software was used to analyze the images to measure wound closure. The wound healing assays was performed in triplicate.

### 2.7. Transwell Assay

Matrigel was diluted with a serum-free culture solution and spread on the bottom of the upper chamber of the Transwell chamber. Chambers were incubated at 37°C for 5 hrs. After 24 hrs of transfection, 500 *μ*l of cell suspension (3 × 10^4^ cells) was added to the upper chamber of the Transwell chamber, and 1 ml of medium containing FBS was added to the lower chamber. After incubating overnight, the Transwell chamber was taken out, and the remaining cells in the upper chamber were wiped with a cotton swab. The invading and migrating cells on the membrane bottom were fixed with 4% paraformaldehyde and stained with 1% crystal violet. Three fields of view were randomly selected under the microscope and photographed for observation, counting, and photography.

### 2.8. Quantitative Reverse Transcription Polymerase Chain Reaction (qRT-PCR)

Cells were collected by centrifugation, and total RNA was extracted using the Ultrapure RNA Kit (CoWin Biotech Co. Ltd., Beijing, China). Then, RNA was reverse transcribed into cDNA using the HiFiScript cDNA Synthesis Kit (CoWin Biotech Co. Ltd., Beijing, China). The reaction conditions were as follows: 42°C for 15 minutes and 85°C for 5 minutes. The SYBR Premix Ex Taq II kit was used for qRT-PCR. The reaction conditions were as follows: 95°C for 10 seconds, 40 cycles of 95°C for 5 seconds, and 60°C for 25 seconds. The primers were as follows: UPK3A-F 5′-TCGACTCAGTCACCCCATACT-3′, UPK3A-R 5′-GAACTCCCCATGTCCACGAG-3′; p53-F 5′-TCAACAAGATGTTTTGCCAACTG-3′, p53-R 5′-ATGTGCTGTGACTGCTTGTAGATG-3′; KLF4-F 5′-CCACCTTCTTCACCCCTAGA-3′, KLF4-R 5′-AAGGTTTCTCACCTGTGTGG-3′; ZMAT3-F 5′-TCCTTCCTGTCTTGCAGGCATTT-3′, ZMAT3-R 5′-GGGAAGCCTGGGGCATAATC-3′; MDM2-F 5′-TCAGGTGATTGGTTGGATCAGG-3′, MDM2-R 5′-AGTGCATTTCCAATAGTCCTCA-3′; SP1-F 5′-CCACCATGAGCGACCAAGAT-3′, SP1-R 5′-TGAAAAGGCACCACCACCAT-3′; GADPH-F 5′-GGATTTGGTCGTATTGGGCG-3′; and GADPH-R 5′-TCCCGTTCTCAGCCATGTAGT-3′. GADPH was used as the internal reference gene. The data obtained were analyzed by the 2^−ΔΔCt^ method. Each experiment was performed in triplicate.

### 2.9. Western Blot Assay

Total protein form cells was extracted with RIPA lysis buffer, and the protein concentration of each sample was determined using the BCA kit (CWBIO, Beijing, China). After boiling them at 95°C for 5 minutes, proteins were separated by electrophoresis on a 10% sodium dodecyl sulfate polyacrylamide gel. The resolved proteins were transferred onto a PVDF membrane, which was then blocked with 5% skim milk for 1 h at room temperature. The PVDF was then incubated with an appropriate primary antibody at 4°C overnight, washed with TBST for 3 × 5 minutes, and incubated with a secondary antibody for 1 h at room temperature. After washing the membrane with TBST, the ECL kit (Tanon Science and Technology Co., Ltd., Shanghai, China) was used to develop the protein bands. Protein levels were semiquantitatively analyzed using the Image J software (v1.48; National Institutes of Health). The antibodies used were as follows: primary antibodies were anti-UPK3A, anti-p-p53, anti-KLF4, anti-ZMAT3, anti-MDM2, anti-SP1, and anti-GAPDH (Sigma-Aldrich, St. Louis, USA); secondary antibody was goat anti-rabbit IgG antibody (Abcam, USA).

### 2.10. Statistical Analysis

All experiments were performed in triplicate. The experimental data are expressed as mean ± SD and were analyzed using the GraphPad Prism software (version 7.0; GraphPad Software, Inc.). Statistically significant differences between groups were analyzed using Student's *t* test or one-way ANOVA, and differences with a *P* value less than 0.05 were considered statistically significant.

## 3. Results

### 3.1. UPK3A Expression Levels in Gastric Tissues and Cell Lines

**Figure 1:**
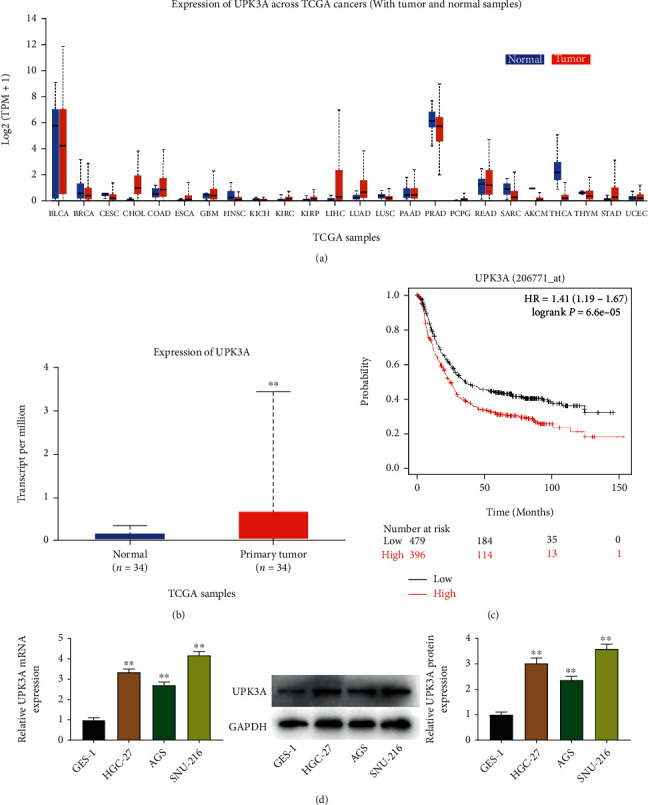
UPK3A expression levels in gastric cancer cell lines. (a) The expression of UPK3A in cancers was assessed by TCGA database. (b) The expression of UPK3A in gastric cancer was assessed by TCGA database. (c) Prognosis value of UPK3A was analyzed by Kaplan-Meier plotter analysis. (d) The UPK3A level in GES-1, SNU-216, AGS, and HGC-27 cells was measured by qPCR and Western blot assays. ^∗∗^*P* < 0.01 vs. GES-1 cells.

Data from TCGA database and Kaplan-Meier plotter analysis were used to assess the expression and prognostic value of UPK3A. UPK3A was markedly upregulated in gastric cancer tissues compared to that in normal tissues (*P* < 0.01, Figures 1(a) and 1(b)). Besides, the overall survival of patients with gastric cancer was analyzed based on TCGA database. As shown in Figure 1(c), a total of 1222 patients with gastric cancer were divided into high-expression group (*n* = 497) and low-expression group (*n* = 698) by the median survival. The analysis results indicated that UPK3A levels were significantly correlated with the prognosis of patients with gastric cancer (*P* < 0.001) and that patients with high UPK3A levels had poor prognoses (Figure 1(c)).

The next is the mRNA and protein levels of UPK3A in GES-1, SNU-217, AGS, and HGC-27 cells by qRT-PCR and Western blot, respectively. UPK3A was highly expressed in SNU-216, AGS, and HGC-27 cells compared to that in GES-1 cells (*P* < 0.01, Figure 1(d)). As the levels of UPK3A were highest in the SNU-216 and HGC-27 cell lines, these cell lines were used in subsequent experiments.

### 3.2. Silencing of UPK3A Inhibits the Activity and Proliferation of Gastric Cancer Cells

**Figure 2:**
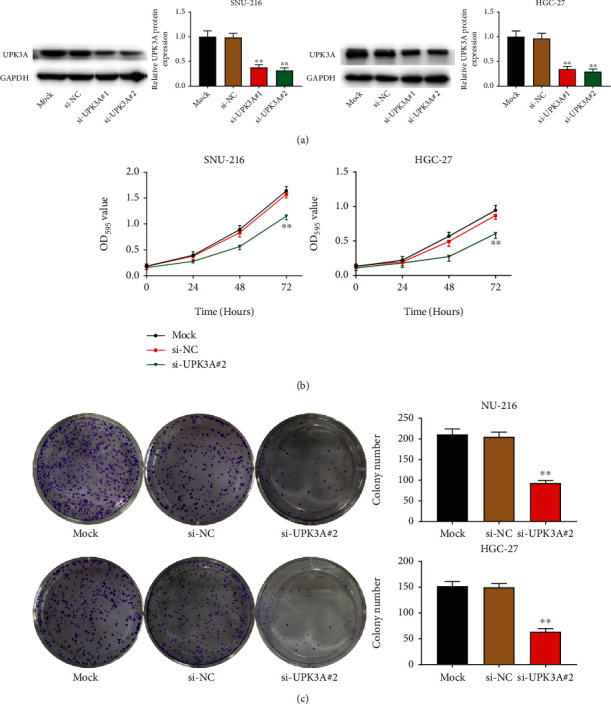
Silencing of UPK3A inhibits the activity and proliferation of gastric cancer cells. (a) The expression of UPK3A both in SNU-216 and HGC-27 cells was detected by Western blot assay. (b) The cell viability of SNU-216 and HGC-27 was detected by MTT assay. (c) The cell growth of SNU-216 and HGC-27 cells was measured by colony formation assay. ^∗∗^*P* < 0.01 vs. Mock group and si-NC group.

To achieve a preliminary understanding of the specific role played by UPK3A in the gastric cancer progression, SNU-216 and HGC-27 cells were transfected with UPK3A siRNAs (si-UPK3A#1 and si-UPK3A#2). Both siRNAs significantly inhibited UPK3A expression in SNU-216 and HGC-27 cells, especially si-UPK3A#2, compared to the Mock and si-NC groups (*P* < 0.01, Figure 2(a)). Thus, si-UPK3A#2 was used in subsequent experiments. Results of the MTT assay showed that silencing of UPK3A decreased the cell viability of SNU-216 and HGC-27 cells (*P* < 0.01, Figure 2(b)). The colony formation results showed that silencing of UPK3A significantly decreased the colony number of SNU-216 and HGC-27 cells (*P* < 0.01, Figure 2(c)).

### 3.3. UPK3A Silencing Suppresses the Migration and Invasion of Gastric Cancer Cells

**Figure 3:**
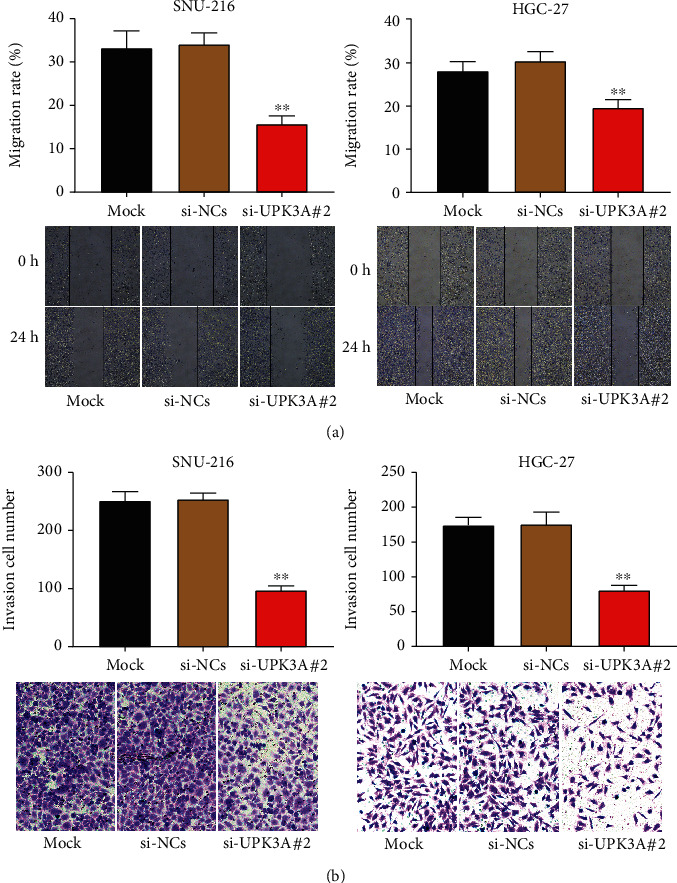
Downregulated UPK3A suppresses the migration and invasion of gastric cancer cells. (a) The migration ability of SNU-216 and HGC-27 cells was detected by wound healing assay. (b) The invasion ability of SNU-216 and HGC-27 cells was measured by Transwell assay. ^∗∗^*P* < 0.01 vs. Mock group and si-NC group.

To illustrate the effect of UPK3A on the migration and invasion of gastric cancer cells, wound healing and Transwell assays were performed. UPK3A-silencing strongly inhibited the migration of SNU-216 and HGC-27 cells compared to the Mock and si-NC group (*P* < 0.01, Figure 3(a)). Cell invasion was also inhibited by UPK3A silencing (*P* < 0.01, Figure 3(b)). Collectively, silencing of UPK3A inhibited the migration and invasion of gastric cancer cells, indicating that UPK3A promotes the progression of gastric cancer.

### 3.4. UPK3A-Mediated Promotion of Gastric Cancer Is Influenced by p53 Signaling Pathway

**Figure 4:**
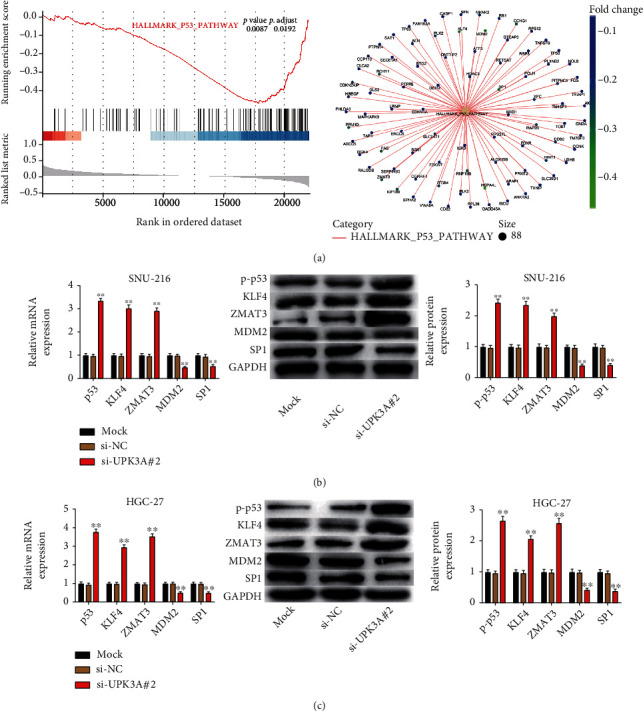
Tumor promoting property by UPK3A is mediated by p53 signaling pathway. (a) The pathways in which UPK3A involved were analyzed on Enriched KEGG pathway analysis. (b, c) The expression of p-p53, KLF4, ZMAT3, MDM2, and SP1 in SNU-216 and HGC-27 cells were detected using qRT-PCR and Western blot assay. ^∗∗^*P* < 0.01 vs. Mock group and si-NC group.

KEGG pathway enrichment analysis was performed to explore the downstream signaling pathway that is modulated by UPK3A in gastric cancer. The results indicated that UPK3A suppressed the activation of p53 signaling pathway (Figure 4(a)). QRT-PCR and Western blot assay showed that the expression of p53 and other genes involved in p53 signaling (KLF4 and ZMAT3) was significantly upregulated in SNU-216 and HGC-27 cells in the si-UPK3A#2 group, while the expression of additional genes involved in p53 signaling (MDM2 and SP1) was significantly decreased in the si-UPK3A#2 group compared to the Mock and si-NC groups (*P* < 0.01, Figures 4(b) and 4(c)).

### 3.5. Inhibition of p53 Signaling Pathway Reverses the Effects of UPK3A Silencing in Gastric Cancer Cells

**Figure 5:**
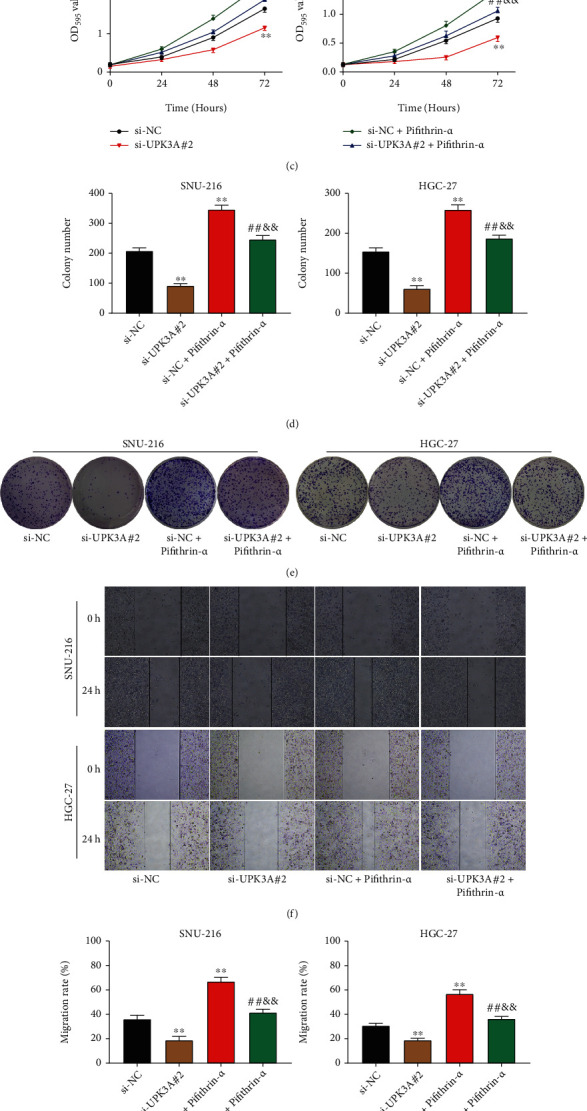
Inhibition of p53 signaling pathway eliminates the effect of UPK3A knockdown on gastric cancer cells. (a) The protein expression of UPK3A in SNU-216 and HGC-27 cells was detected by western blot assay. (b) The protein expression of p-p53, KLF4, ZMAT3, MDM2, and SP1 in SNU-216 and HGC-27 cells was detected by Western blot assay. (c) The cell activity of SNU-216 and HGC-27 cells was analyzed by MTT assay. (d, e) The cell proliferation of SNU-216 and HGC-27 cells was analyzed by colony formation assay. (f, g) The cell migration of SNU-216 and HGC-27 cells was detected by wound healing assay. (h, i) The cell invasion of SNU-216 was measured by Transwell assay. ^∗∗^*P* < 0.01 vs. si-NC group; ^##^*P* < 0.01 vs. si-UPK3A#2 group; ^&&^*P* < 0.01 vs. si-UPK3A#2+Pifithrin-*α* group.

To determine whether the function of UPK3A is directly related to the p53 signaling pathway, the p53 signaling pathway inhibitor Pifithrin-*α* was employed in follow-up experiments. Pifithrin-*α* had no significant effect on UPK3A protein expression in SNU-216 and HGC-27 cells (Figure 5(a), *P* > 0.05). Pifithrin-*α* treatment markedly inhibited the levels of p-p53, KLF4, and ZMAT3 in SNU-216 and HGC-27 cells and enhanced the expression of MDM2 and SP1 (*P* < 0.01, Figure 5(b)). Moreover, Pifithrin-*α* treatment significantly reversed the activation effects of UPK3A silencing on the expression of p-p53, KLF4, ZMAT3, MDM2, and SP1 (*P* < 0.01). Pifithrin-*α* treatment significantly suppressed the viability and proliferation of SNU-216 and HGC-27 cells and reversed the inhibitory effect of siUPK3A#2 on cell viability and proliferation (*P* < 0.01, Figures 5(c)–5(e)). Besides, Pifithrin-*α* treatment significantly enhanced the migration and invasion abilities of SNU-216 and HGC-27 cells and eliminated the inhibitory effect of UPK3A silencing on the migration and invasion ability of gastric cancer cells (*P* < 0.01, Figures 5(f)–5(i)). These results indicated that the p53 signaling pathway mediates the oncogenic role of UPK3A in the progression of gastric cancer.

### 3.6. Silencing of p53 Reverses the Effects of UPK3A Knockdown in Gastric Cancer Cells

**Figure 6:**
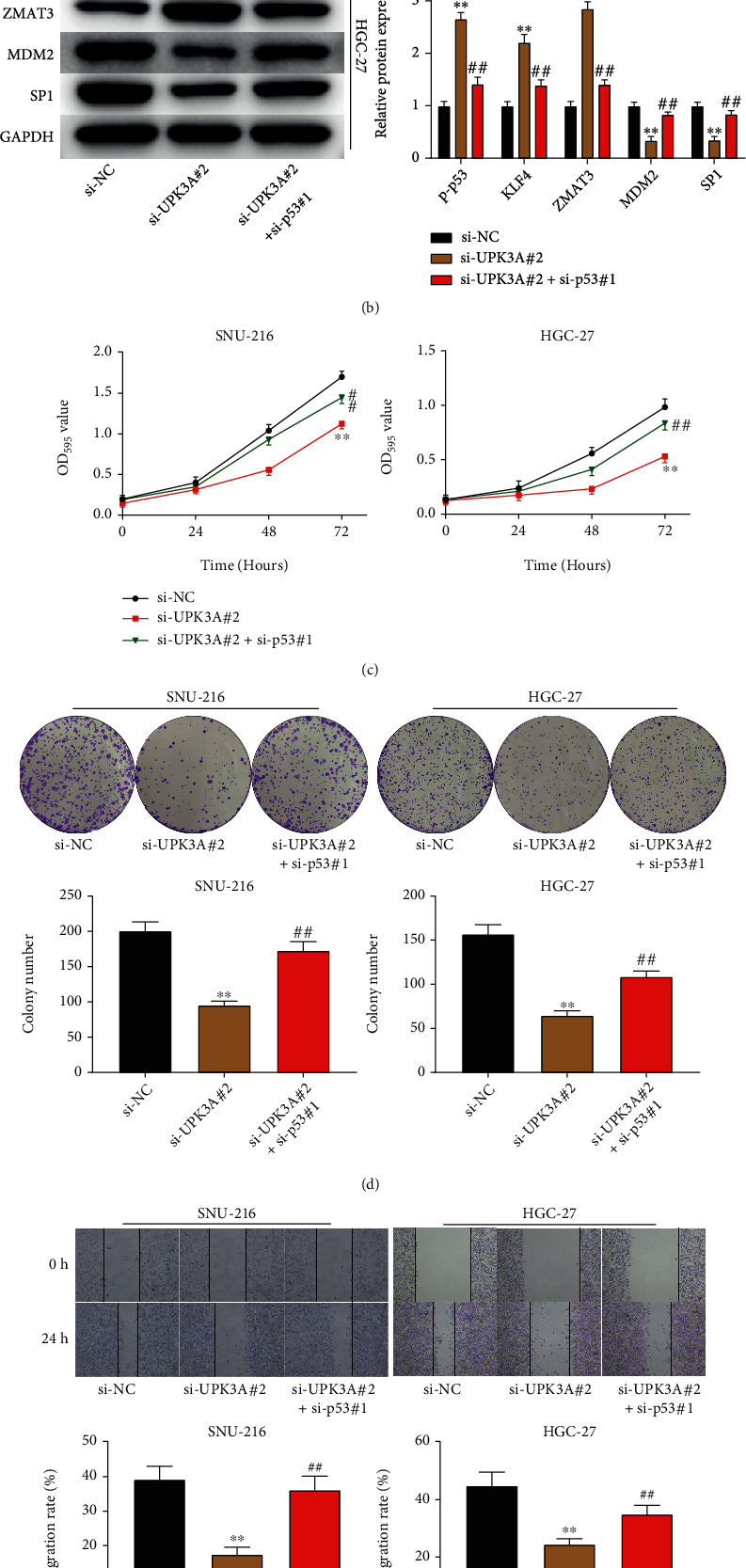
Silencing of p53 reverses the effects of UPK3A knockdown in gastric cancer cells. (a) The expression of p53 in SNU-216 and HGC-27 cells was detected by qRT-PCR and Western blot assay. (b) The protein expression of p-p53, KLF4, ZMAT3, MDM2, and SP1 in SNU-216 and HGC-27 cells was detected by Western blot assay. (c) The cell activity of SNU-216 and HGC-27 cells was analyzed by MTT assay. (d) The cell proliferation of SNU-216 and HGC-27 cells was analyzed by colony formation assay. (e) The cell migration of SNU-216 and HGC-27 cells was detected by wound healing assay. (f) The cell invasion of SNU-216 was measured by Transwell assay. ^∗∗^*P* < 0.01 vs. si-NC group; ^##^*P* < 0.01 vs. si-UPK3A#2 group.

Then, p53 siRNAs were transfected into SNU-216 and HGC-27 cells to further confirm the regulation of UPK3A on the p53 signaling pathway. The expression of p53 was markedly decreased by p53 siRNAs, especially the si-p53#1 (*P* < 0.01, Figure 6(a)). The data of Western blot showed that p53 silencing revered the activatory effect of UPK3A silencing on p53 signaling pathway and was shown by the decrease of the expression levels of p53, KLF4, and ZMAT3 in SNU-216 and HGC-27 cells and increase of the expression levels of MDM2 and SP1 in si-UPK3A#2+si-p53#1 group (*P* < 0.01, Figure 6(b)). The data in Figures 6(c) and 6(d) showed that p53 silencing reversed the inhibitory effect of UPK3A silencing on cell viability and proliferation of SNU-216 and HGC-27 cells (*P* < 0.01). The results of wound healing and Transwell assays showed that p53 silencing significantly promoted migration and invasion and reversed the inhibitory effect of UPK3A silencing on the migration and invasion of gastric cancer cells (*P* < 0.01, Figures 6(e) and 6(f)). These data indicated that UPK3A silencing inhibited the proliferation, migration, and invasion of gastric cancer cells by activating the p53 signaling pathway.

## 4. Discussion

Gastric cancer is a common malignancy of the digestive system [16]. Chemotherapy is an important treatment for advanced gastric cancer, but the combination with cytotoxic drugs does not fundamentally improve the effectiveness of chemotherapy [17, 18]. With recent advances in research on the molecular mechanisms underlying the occurrence and development of gastric cancer, molecular targeted therapy is gradually being adopted for the treatment of gastric cancer [19].

In the present study, the oncogenic function of UPK3A in human gastric cancer cells was investigated. In 2010, studies showed for the first time that UPK3A can serve as a biomarker for bladder cancer [10, 20]. Patients with bladder cancer had high UPK3A levels in the urine. These studies showed that the quantification of UPK3A levels in urine represents an effective and sensitive method for diagnosing of bladder cancer [10]. In our study, we first examined the effect of UPK3A on gastric cancer cells. UPK3A expression was significantly upregulated in gastric cancer cells. The viability, proliferation, migration, and invasion of SNU-216 and HGC-27 were significantly decreased upon UPK3A silencing, indicating that UPK3A promotes the progression of gastric cancer.

p53 is closely associated with the occurrence of tumors [21, 22]. A previous study showed that up to 40% of human tumors are associated with p53 mutations [23]. Most studies suggested that p53 inhibits cell proliferation and promotes apoptosis [24, 25]. KLF4 is a member of the Krüppel-like factor (KLF) family of proteins and acts as a tumor suppressor in colon, bladder, lung, gastric, and intestinal cancers [26, 27]. ZMAT3 is a tumor suppressor gene that acts downstream of p53 and regulates cell proliferation and survival [28]. MDM2 plays an important role in downregulating p53 activity via ubiquitin-dependent degradation [29]. The abnormal activation of SP1 upregulates the expression of tumor-related factors and promotes the proliferation and metastasis of colon, gastric, and pancreatic tumors [30–32]. In our study, the p53 signaling pathway was suppressed by UPK3A. UPK3A silencing promoted the activity of the p53 signaling pathway. The levels of p53, KLF4, and ZMAT3 were increased, while those of MDM2 and SP1 were decreased upon UPK3A silencing. However, the activatory effect of UPK3A silencing on p53 signaling pathway was reversed by p53 silencing. These results suggested that silencing of UPK3A might inhibit the progression of gastric cancer by activating the p53 signaling pathway.

Pifithrin-*α* is a p53 inhibitor that exerts its physiological effects by preventing nuclear translocation of p53 [33]. Our results showed that Pifithrin-*α* treatment inhibited the expression of p53, KLF4, and ZMAT3, while promoting that of MDM2 and SP1. Inhibition of p53 reduced the viability, colony number, migration rate, and invasion of gastric cancer cells. In addition, p53 suppression eliminated the inhibitory effect of UPK3A silencing on gastric cancer cells. The above-mentioned results suggested that the oncogenic effect of UPK3A in gastric cancer is mediated by p53 inhibition.

In recent years, significant advances have been made in the field of molecular targeted therapy with regard to colorectal cancer, lung cancer, breast cancer, and other tumors, resulting in its approval for clinical use [34–36]. Although targeted therapy for gastric cancer is relatively new, significant progress has been made in this field, for example, the development of HER2 monoclonal antibodies and inhibitors of epidermal growth factor receptors, angiogenesis, multitarget tyrosine kinases, cyclin-dependent kinases, mTOR, c-Met, matrix metalloproteinases, IGF-1R, and HSP 90 [37]. In the present study, we demonstrated that UPK3A is highly expressed in gastric cancer cells, and UPK3A silencing represses the proliferation and migration of gastric cancer cells. These findings suggested that UPK3A could be a potential therapeutic target for the treatment of gastric cancer.

However, there are still some limitations in this study. One is that the effect of UPK3A overexpression in gastric cancer has not been explored; the other is that the specific mechanism of UPK3A as a transmembrane protein regulating the p53 signaling pathway has not been explored. Besides, the effect of UPK3A in p53 wild-type gastric and other cancer cell lines need to be investigated in future studies. There are some hypotheses which might provide direction for future research with regard to the specific mechanism of UPK3A regulating p53 signal pathway: UPK3A may regulate p53 signaling pathway through receptors located on the cell membrane or inducing inflammatory response.

In conclusion, UPK3A silencing inhibits the viability, proliferation, invasion, and migration of gastric cancer cells and promotes activation of the p53 signaling pathway. These novel findings provide new avenues for the development of gastric cancer therapeutics.

## Data Availability

The data used to support the findings of this study were supplied by Deliang Xu under license and so cannot be made freely available. Requests for access to these data should be made to Deliang Xu (email: deliang1968@126.com).

## Abbreviations

CDKs: Cyclin dependence sex kinases
UPK3A: Uroplakin 3A.

## Supplementary Materials

Supplementary Materials Supplementary Figure 1: the web page screenshot of the UPK3A expression. Supplementary Figure 2: the UPK3A expression in gastric cancer that analyzed by GEPIA2. Supplementary Figure 3: the correlationship among p53 signaling pathway-related genes. (A) The correlationship between p53 signaling pathway and UPK3A. (B) The correlationship between TP53 and UPK3A. (C) The correlationship between KLF4 and UPK3A. (D) The correlationship between ZMAT3 and UPK3A. (E) The SP1 expression in gastric cancer. (F) The MDM2 expression in gastric cancer. (G) The correlationship between SP1 and MDM2.
