# Supplementary material for: High Expression of UPK3A Promotes the Progression of Gastric Cancer Cells by Inactivating p53 Pathway
Source: Anal Cell Pathol (Amst). 2022 Jun 21;2022:6897561. doi: 10.1155/2022/6897561 (PMC9239834; doi:10.1155/2022/6897561)
Supplement: Supplementary Materials — Supplementary Figure 1: the web page screenshot of the UPK3A expression. Supplementary Figure 2: the UPK3A expression in gastric cancer that analyzed by GEPIA2. Supplementary Figure 3: the correlationship among p53 signaling pathway-related genes. (A) The correlationship between p53 signaling pathway and UPK3A. (B) The correlationship between TP53 and UPK3A. (C) The correlationship between KLF4 and UPK3A. (D) The correlationship between ZMAT3 and UPK3A. (E) The SP1 expression in gastric cancer. (F) The MDM2 expression in gastric cancer. (G) The correlationship between SP1 and MDM2. [file 6897561.f1.zip › 1. web page screenshot of the UPK3A expression.pdf]

UALCAN
Analyze, Integrate, Discover

Home | Tutorial | TCGA | CPTAC | C

Select cancer

- Adrenocortical carcinoma
- Bladder urothelial carcinoma
- Brain lower grade glioma
- Breast invasive carcinoma
- Metastatic breast cancer
- Cervical squamous cell carcinoma
- Cholangiocarcinoma
- Colon adenocarcinoma
- Esophageal carcinoma
- Glioblastoma multiforme
- Head and Neck squamous cell carcinoma
- Kidney Chromophobe
- Kidney renal clear cell carcinoma
- Kidney renal papillary cell carcinoma
- Acute myeloid leukemia

UPK3A expression based on **Sample types**

Expression of UPK3A in STAD based on Sample types

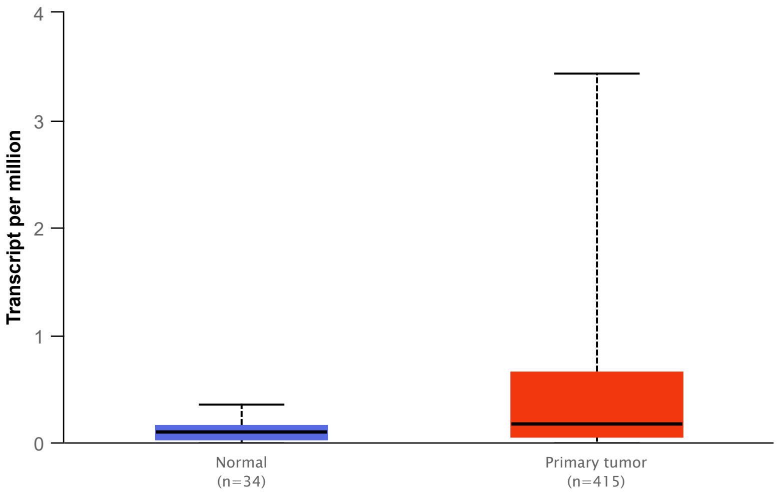

STAD : Stomach adenocarcinomna

Comparison	Statistical significance
Normal-vs-Primary	4.00729999999738E-05

Pan-cancer view **Methylation** **Survival** **Correlated genes** **External L**

UAB MEDICINE | UAB SCHOOL OF MEDICINE Department of Pathology | O'NEAL COMPREHENSIVE CANCER CENTER

Home | Tutorial | Analysis | FAQ | About Us| Contact Us | Citations |

Please cite: Chandrashekar DS, Bashel B, Balasubramanya SAH, Creighton CJ, Rodriguez IP, Chakravarthi BVSK and Varambally S. UALCAN: A pc facilitating tumor subgroup gene expression and survival analyses. Neoplasia. 2017 Aug;19(8):649-658. doi: 10.1016/j.neo.2017.05.002.[PMID:2873
