# Supplementary material for: High Expression of UPK3A Promotes the Progression of Gastric Cancer Cells by Inactivating p53 Pathway
Source: Anal Cell Pathol (Amst). 2022 Jun 21;2022:6897561. doi: 10.1155/2022/6897561 (PMC9239834; doi:10.1155/2022/6897561)
Supplement: Supplementary Materials — Supplementary Figure 1: the web page screenshot of the UPK3A expression. Supplementary Figure 2: the UPK3A expression in gastric cancer that analyzed by GEPIA2. Supplementary Figure 3: the correlationship among p53 signaling pathway-related genes. (A) The correlationship between p53 signaling pathway and UPK3A. (B) The correlationship between TP53 and UPK3A. (C) The correlationship between KLF4 and UPK3A. (D) The correlationship between ZMAT3 and UPK3A. (E) The SP1 expression in gastric cancer. (F) The MDM2 expression in gastric cancer. (G) The correlationship between SP1 and MDM2. [file 6897561.f1.zip › Supplementary figures (1).docx]

**Supplementary methods**

*Bioinformatic analysis*

UPK3A expression in gastric cancer cases and normal samples were analyzed using UALCAN web tool (http://ualcan.path.uab.edu/analysis.html) based on TCGA database (Supplementary figure 1) and GEPIA2 (http://gepia2.cancer-pku.cn/, Supplementary figure 2).

The expression of SP1 and MDM2, and the correlationship between SP1 and MDM2 were analyzed by GEPIA2 (Supplementary figure 3E-G).

The correlationship among UPK3A and p53 signaling pathway related genes was analyzed by GTBAdb (http://guotosky.vip:13838/GTBA/, Supplementary figure 3A-D).

**Supplementary figures**


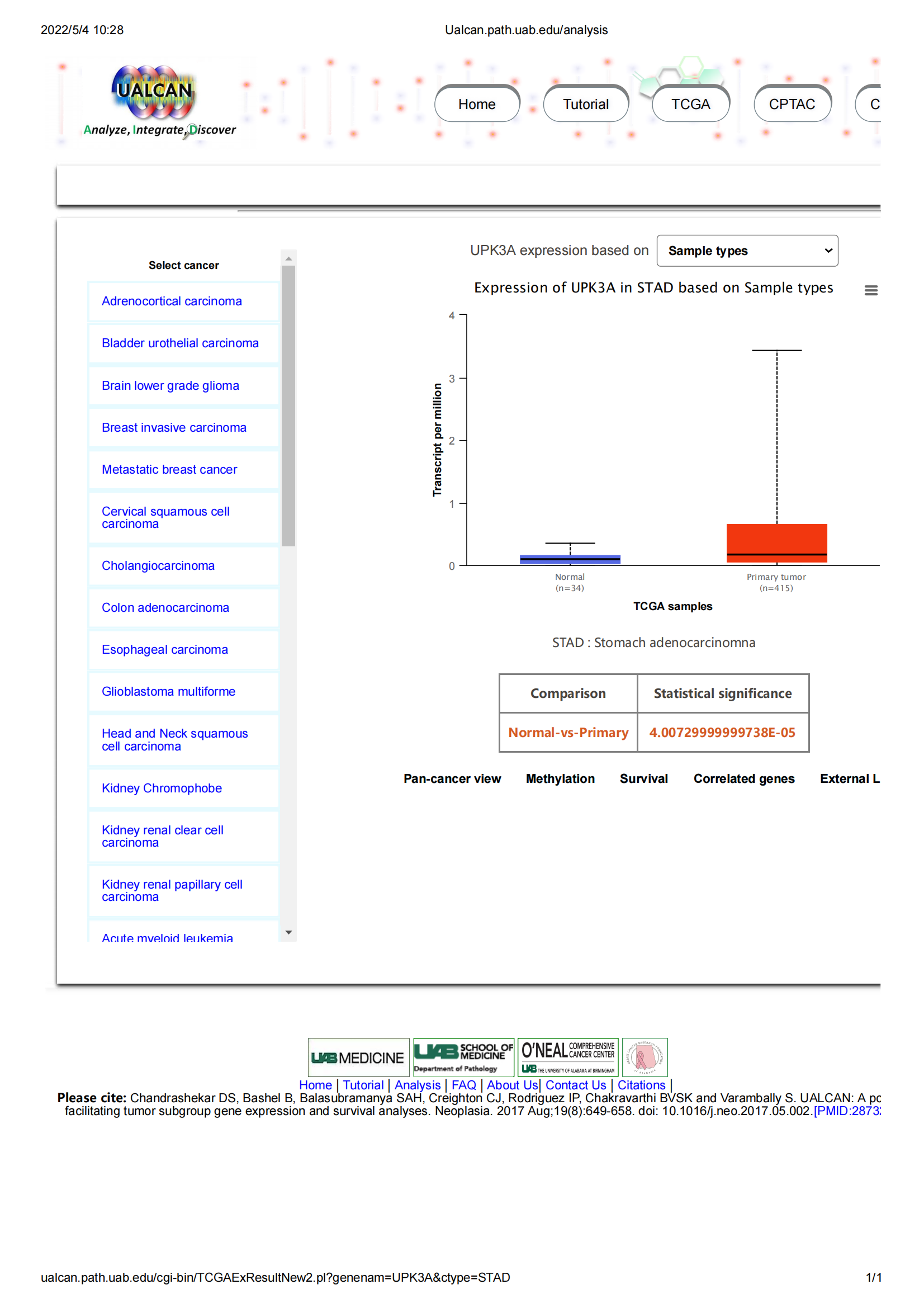


**Supplementary figure 1**: The web page screenshot of the UPK3A expression.


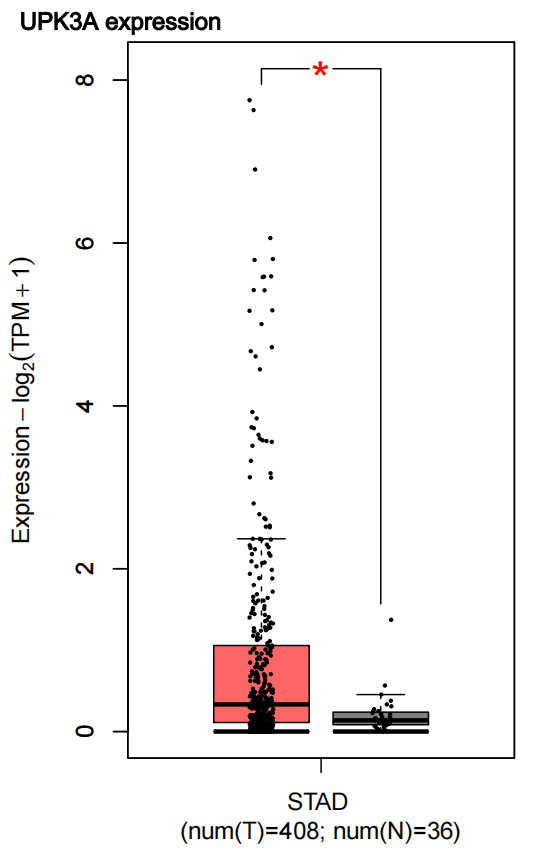


**Supplementary figure 2**: The UPK3A expression in gastric cancer that analyzed by GEPIA2.


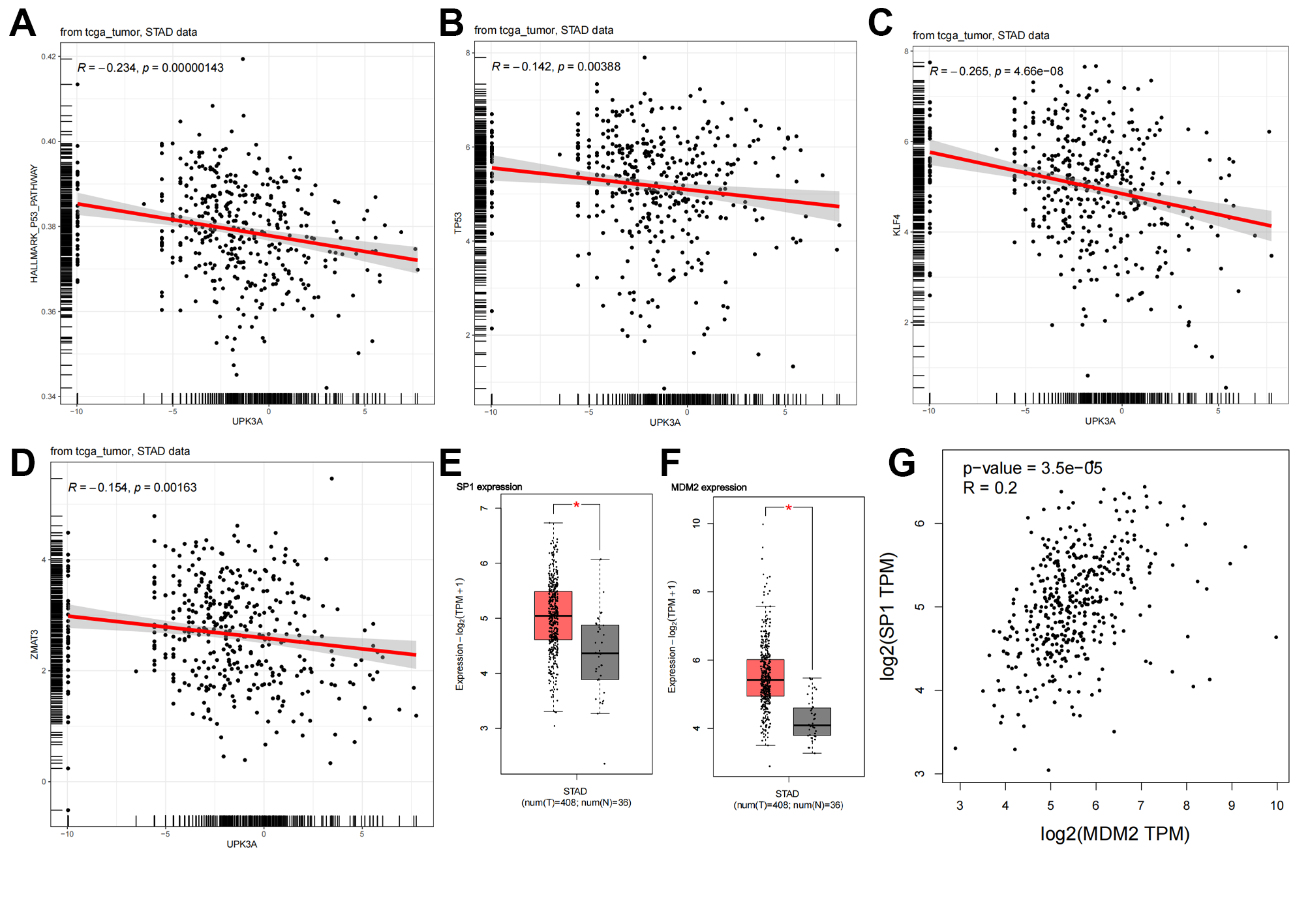


**Supplementary figure 3**: The correlationship among UPK3A and p53 signaling pathway related genes. (A) The correlationship between p53 signaling pathway and UPK3A. (B) The correlationship between TP53 and UPK3A. (C) The correlationship between KLF4 and UPK3A. (D) The correlationship between ZMAT3 and UPK3A. (E) The SP1 expression in gastric cancer. (F) The MDM2 expression in gastric cancer. (G) The correlationship between SP1 and MDM2.
