# Supplementary material for: High Expression of UPK3A Promotes the Progression of Gastric Cancer Cells by Inactivating p53 Pathway
Source: Anal Cell Pathol (Amst). 2022 Jun 21;2022:6897561. doi: 10.1155/2022/6897561 (PMC9239834; doi:10.1155/2022/6897561)
Supplement: Supplementary Materials — Supplementary Figure 1: the web page screenshot of the UPK3A expression. Supplementary Figure 2: the UPK3A expression in gastric cancer that analyzed by GEPIA2. Supplementary Figure 3: the correlationship among p53 signaling pathway-related genes. (A) The correlationship between p53 signaling pathway and UPK3A. (B) The correlationship between TP53 and UPK3A. (C) The correlationship between KLF4 and UPK3A. (D) The correlationship between ZMAT3 and UPK3A. (E) The SP1 expression in gastric cancer. (F) The MDM2 expression in gastric cancer. (G) The correlationship between SP1 and MDM2. [file 6897561.f1.zip › Western blot.docx]

| **Fig 1D** | | **UPK3A** | | **GAPDH** | |
| --- | --- | --- | --- | --- | --- |
|  | | **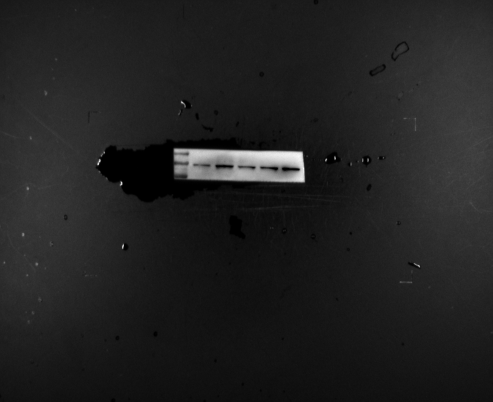** | **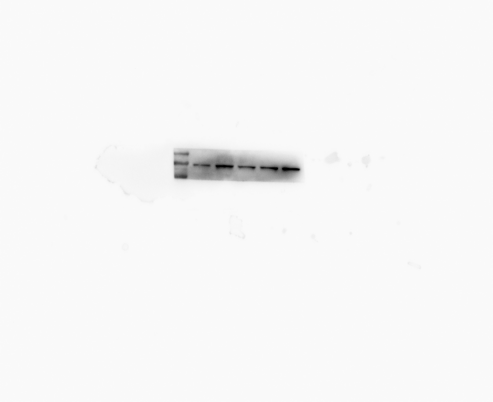** | **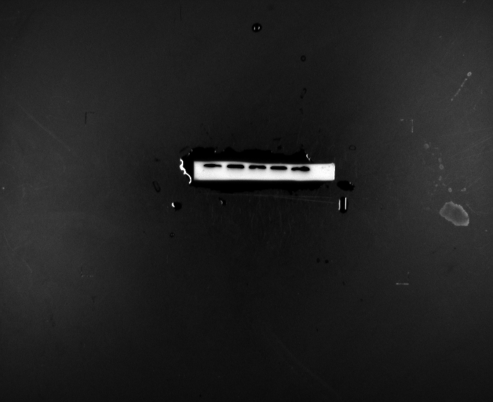** | **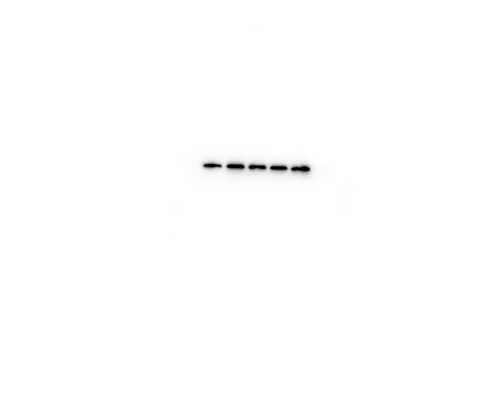** |
| **Fig 2A** | | **UPK3A** | | **GAPDH** | |
| **SNU-216** | | **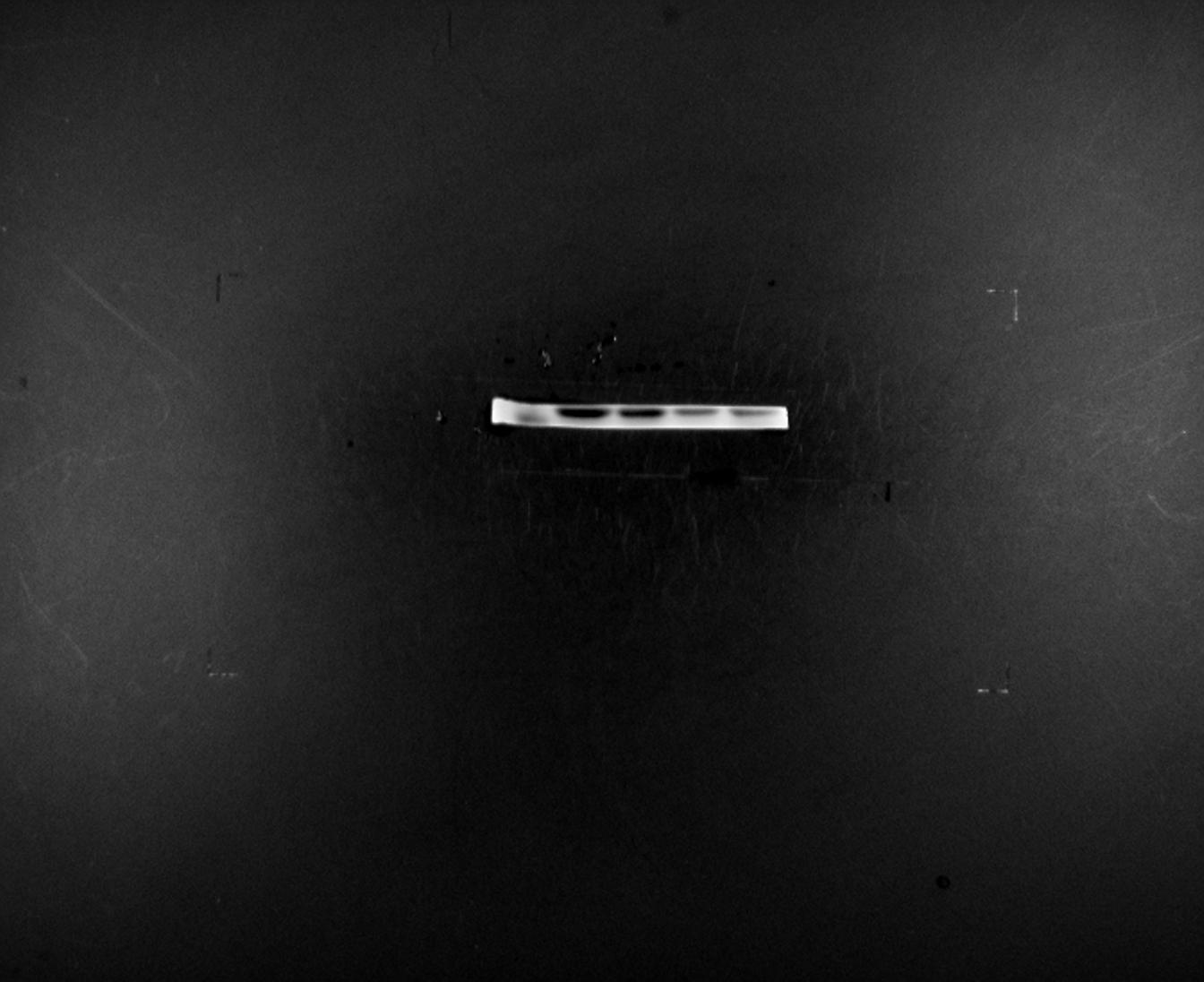** | **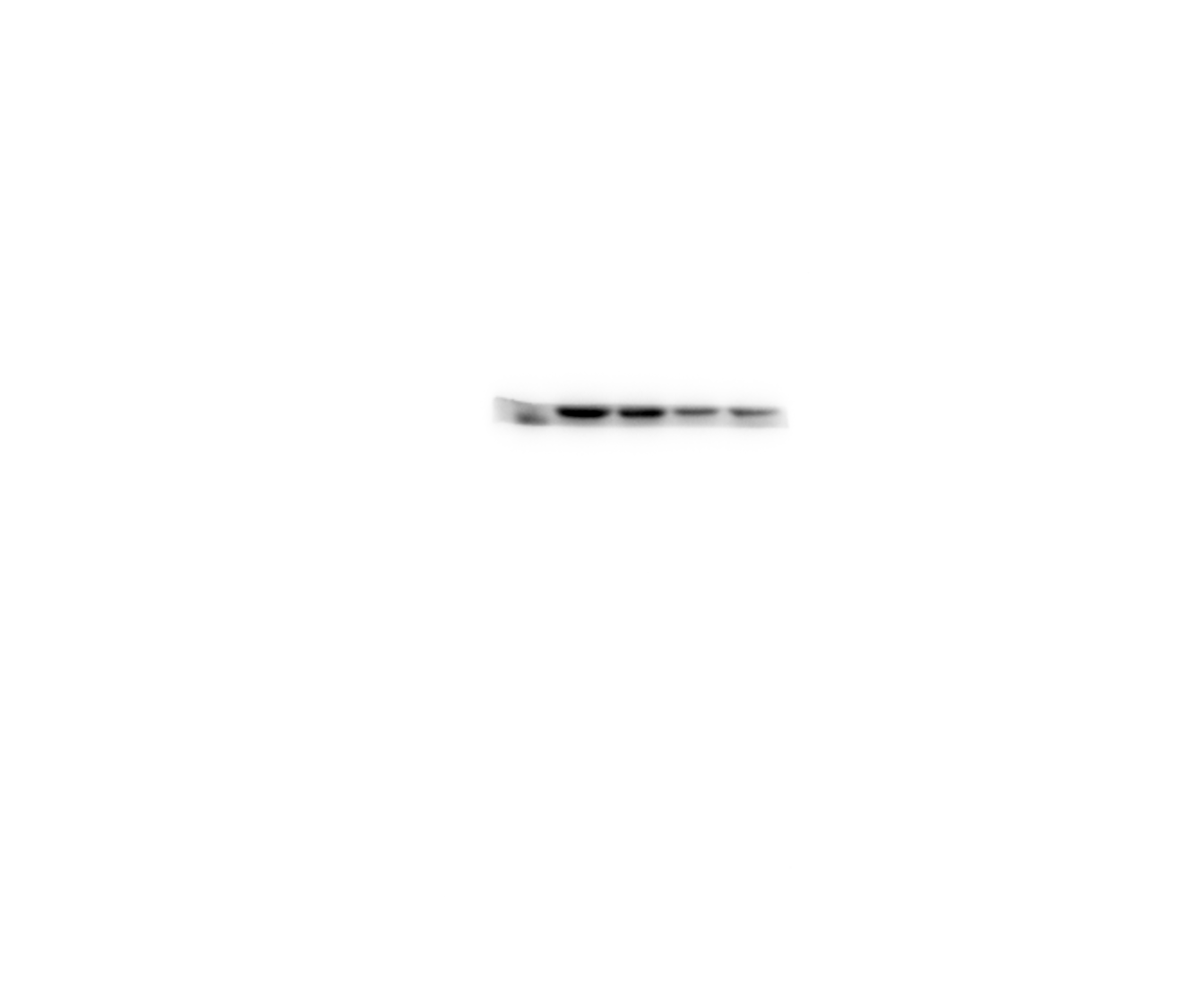** | **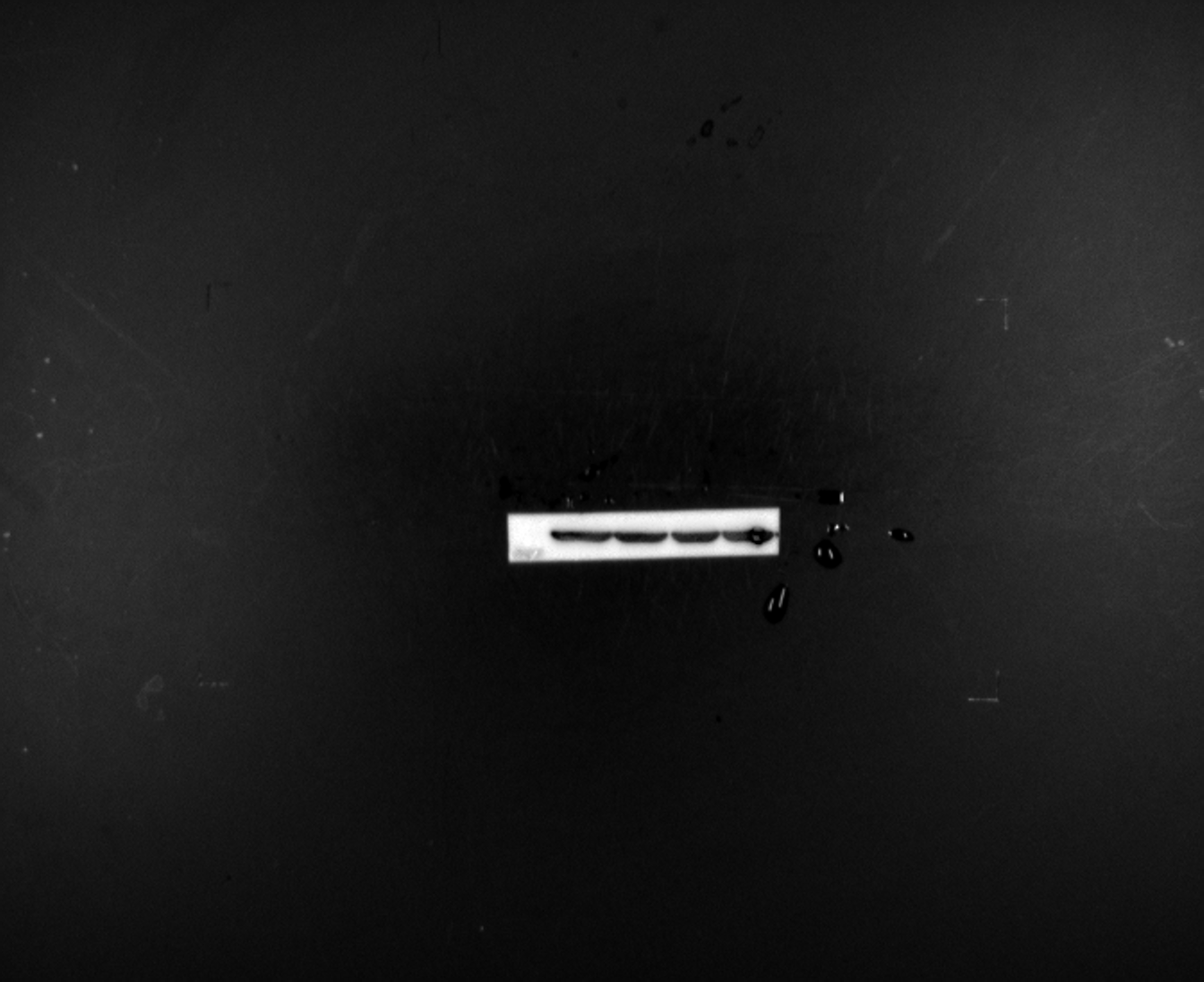** | **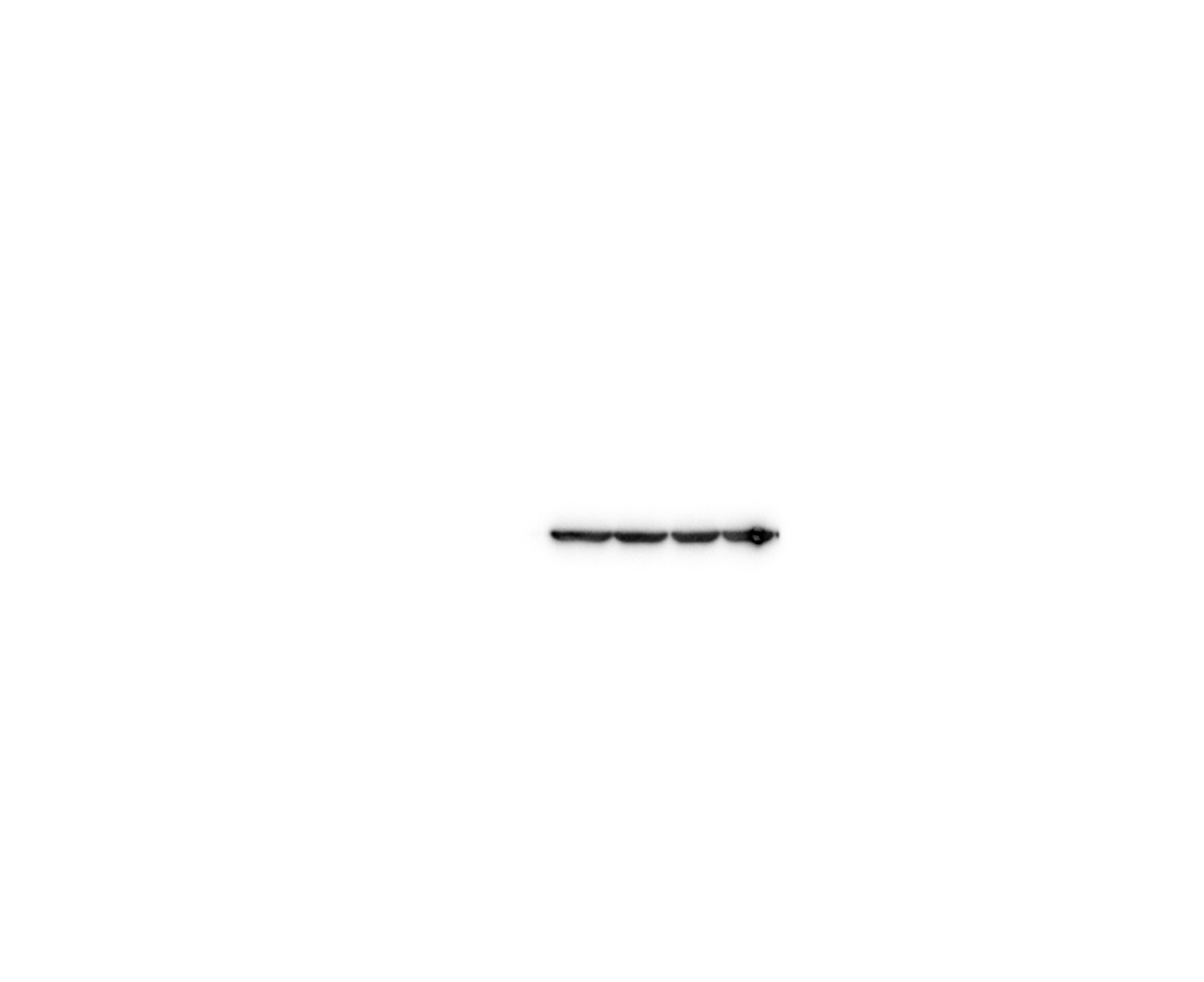** |
| **HGC-27** | | **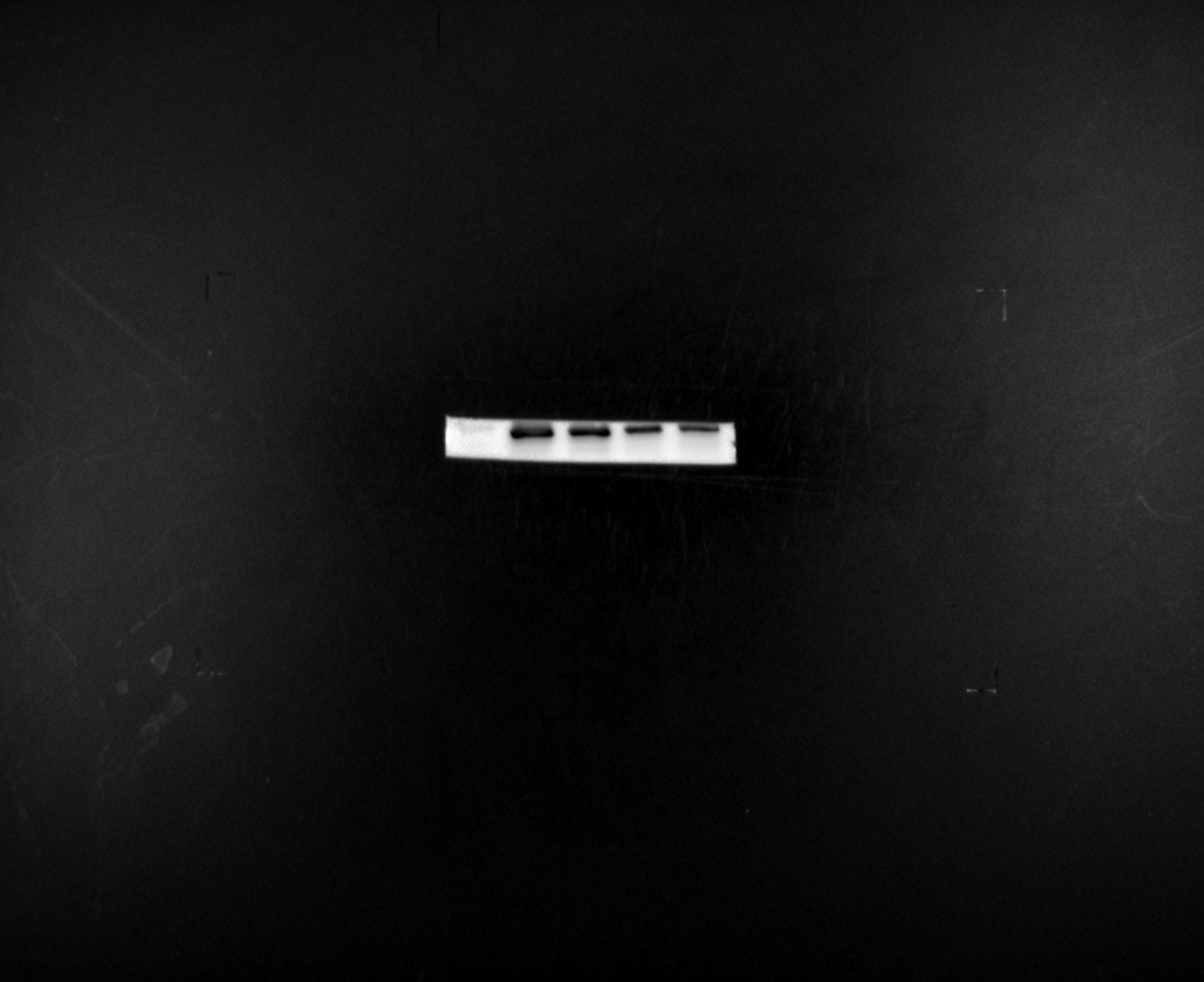** | **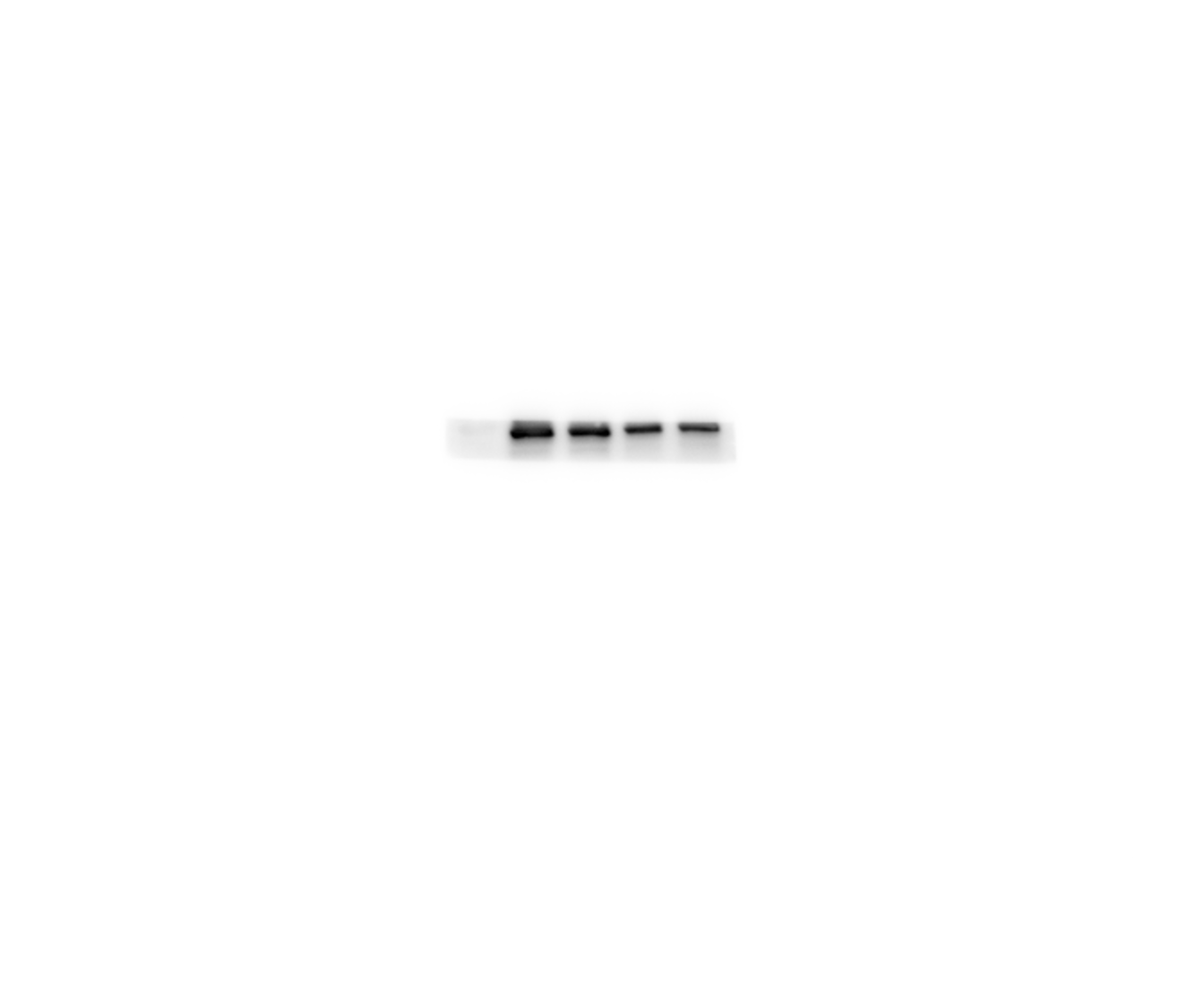** | **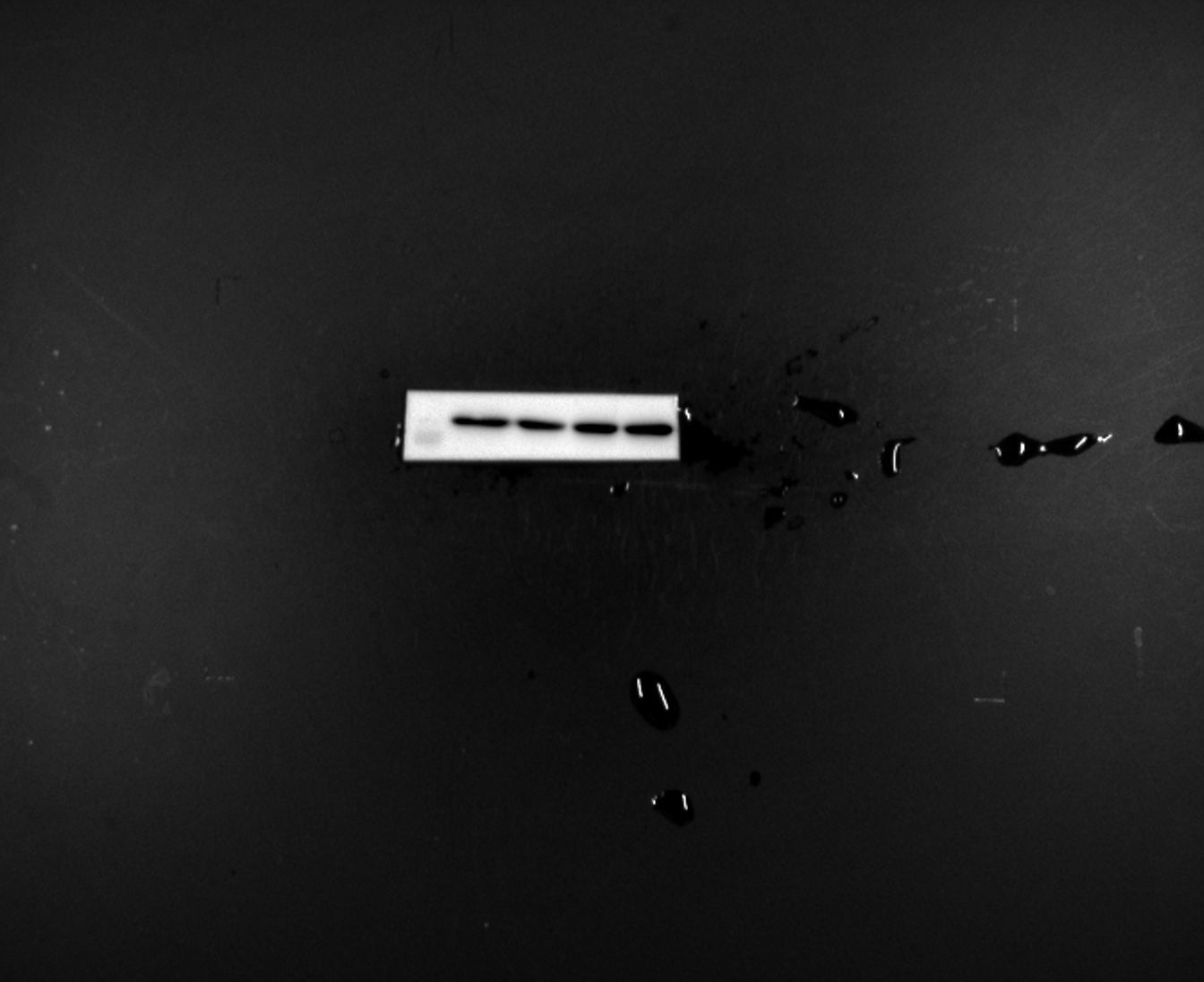** | **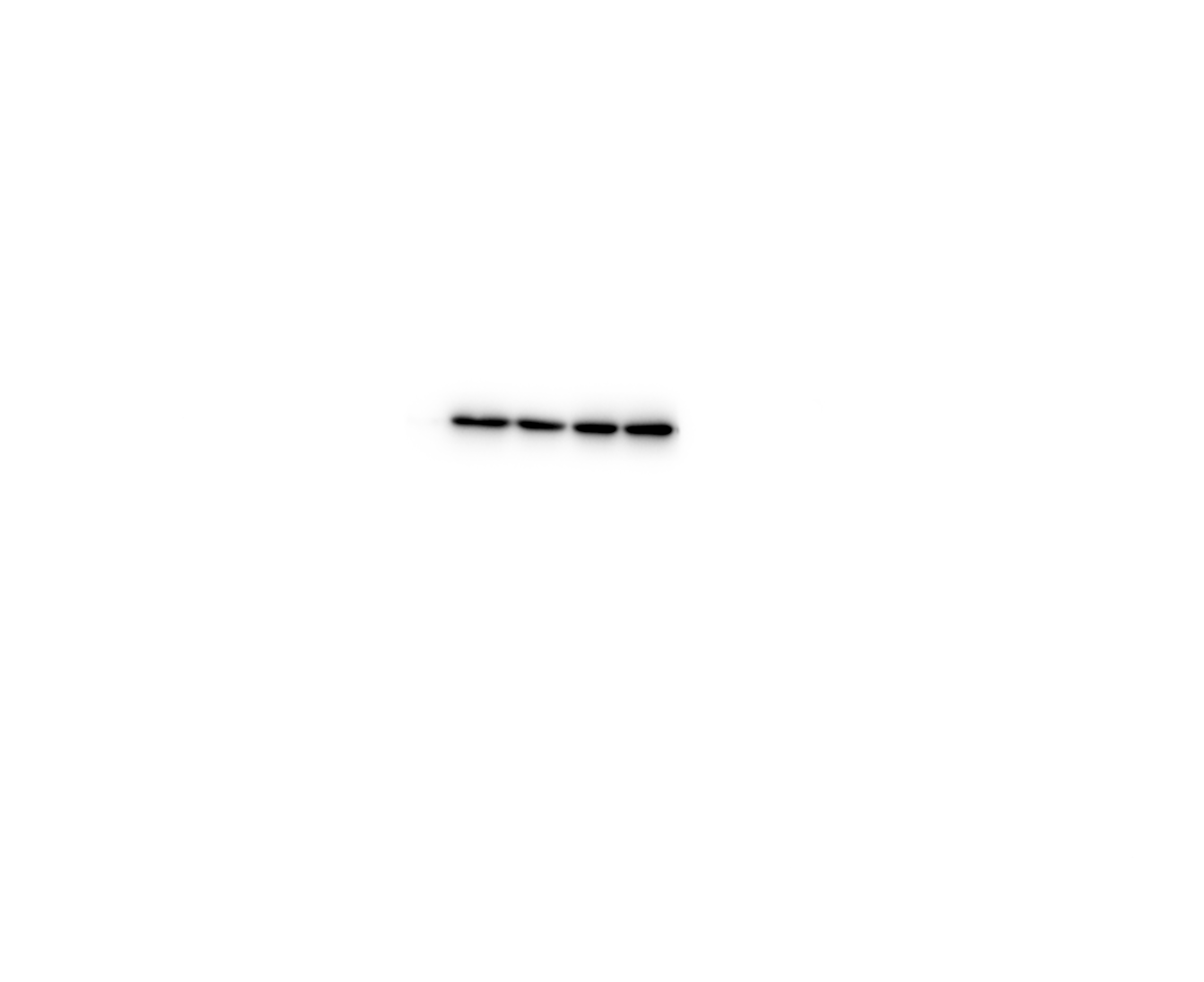** |
| **Fig 4B** | **p-p53** | | | **KLF4** | |
| **SNU-216** | | **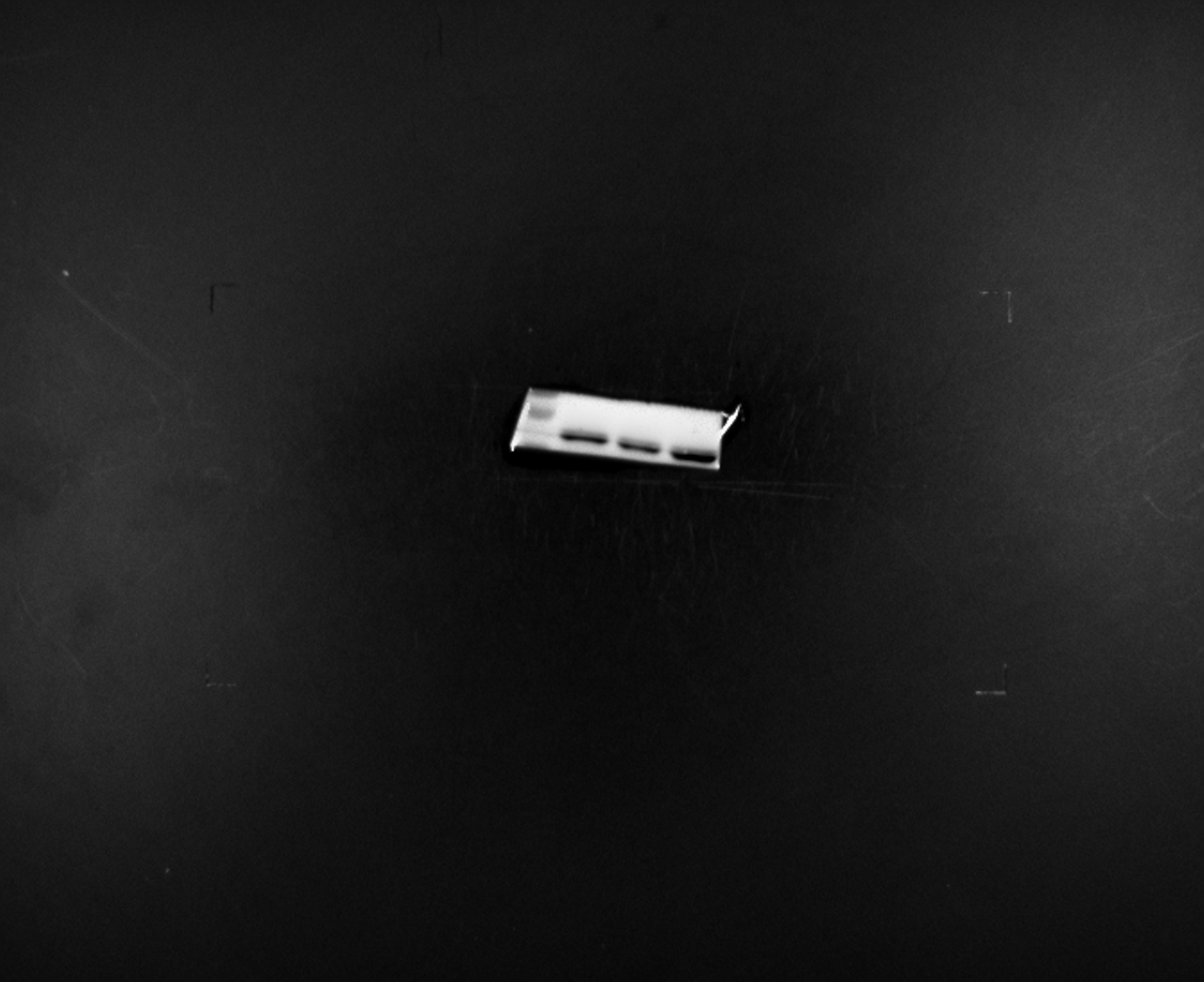** | **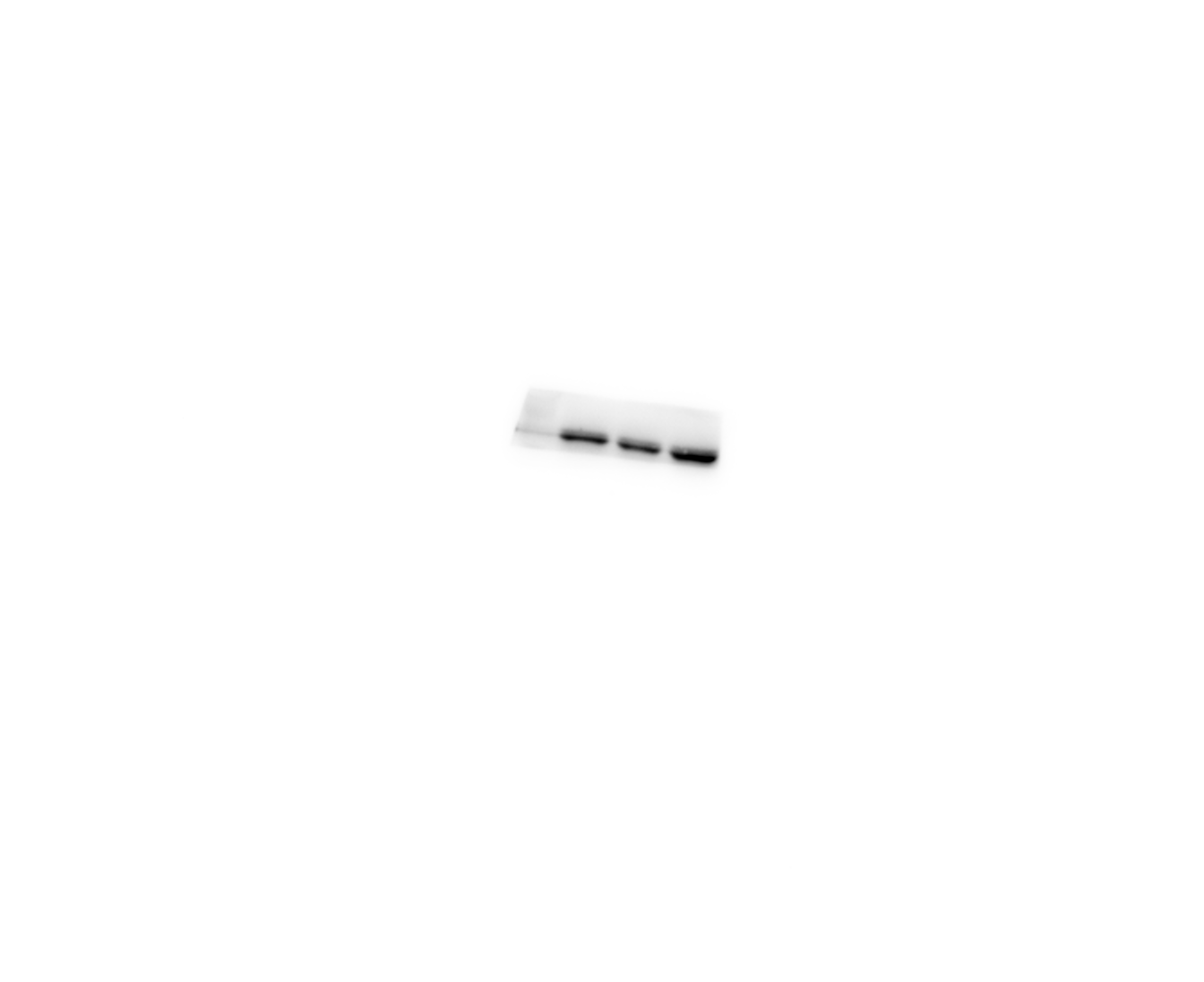** | **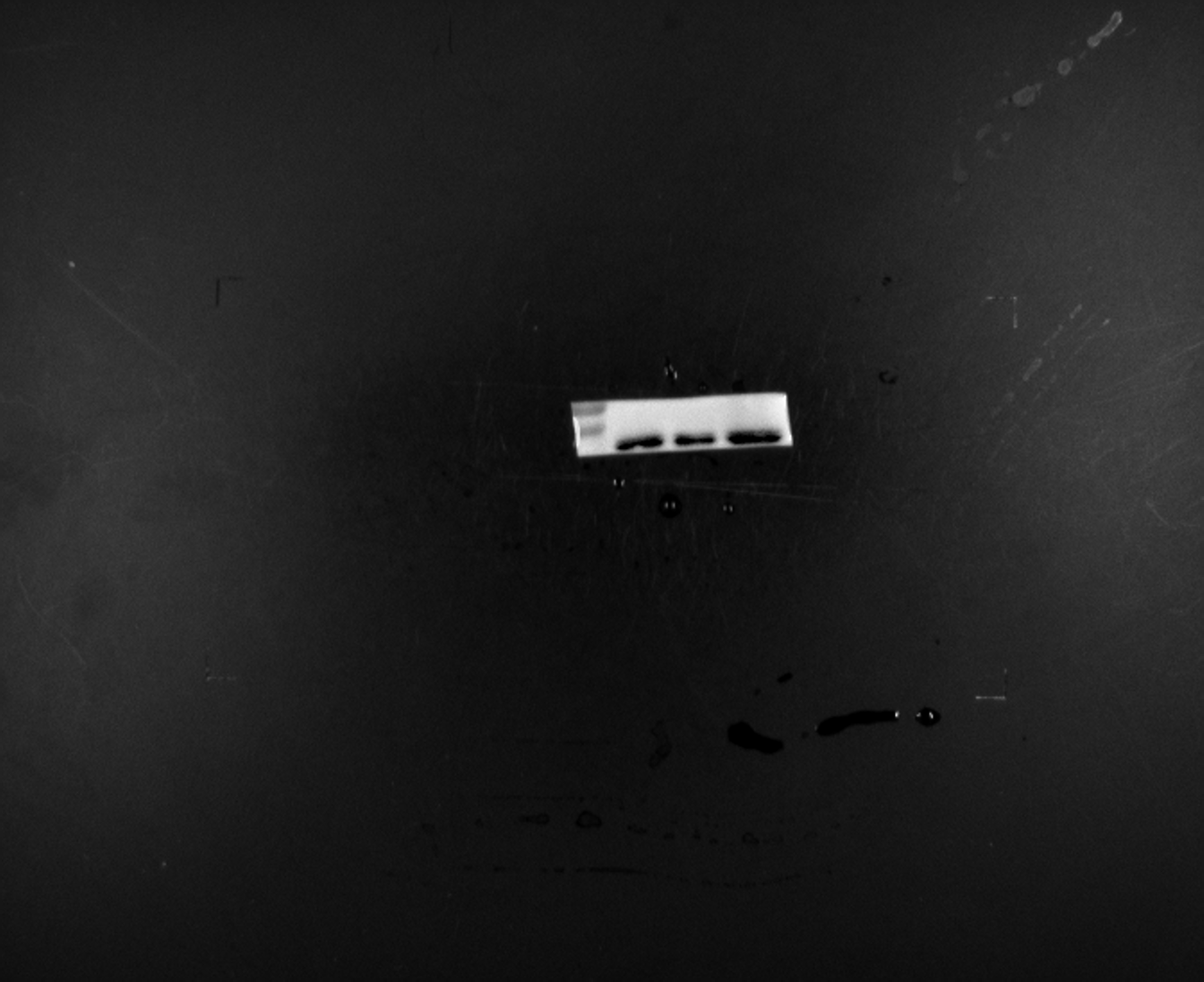** | **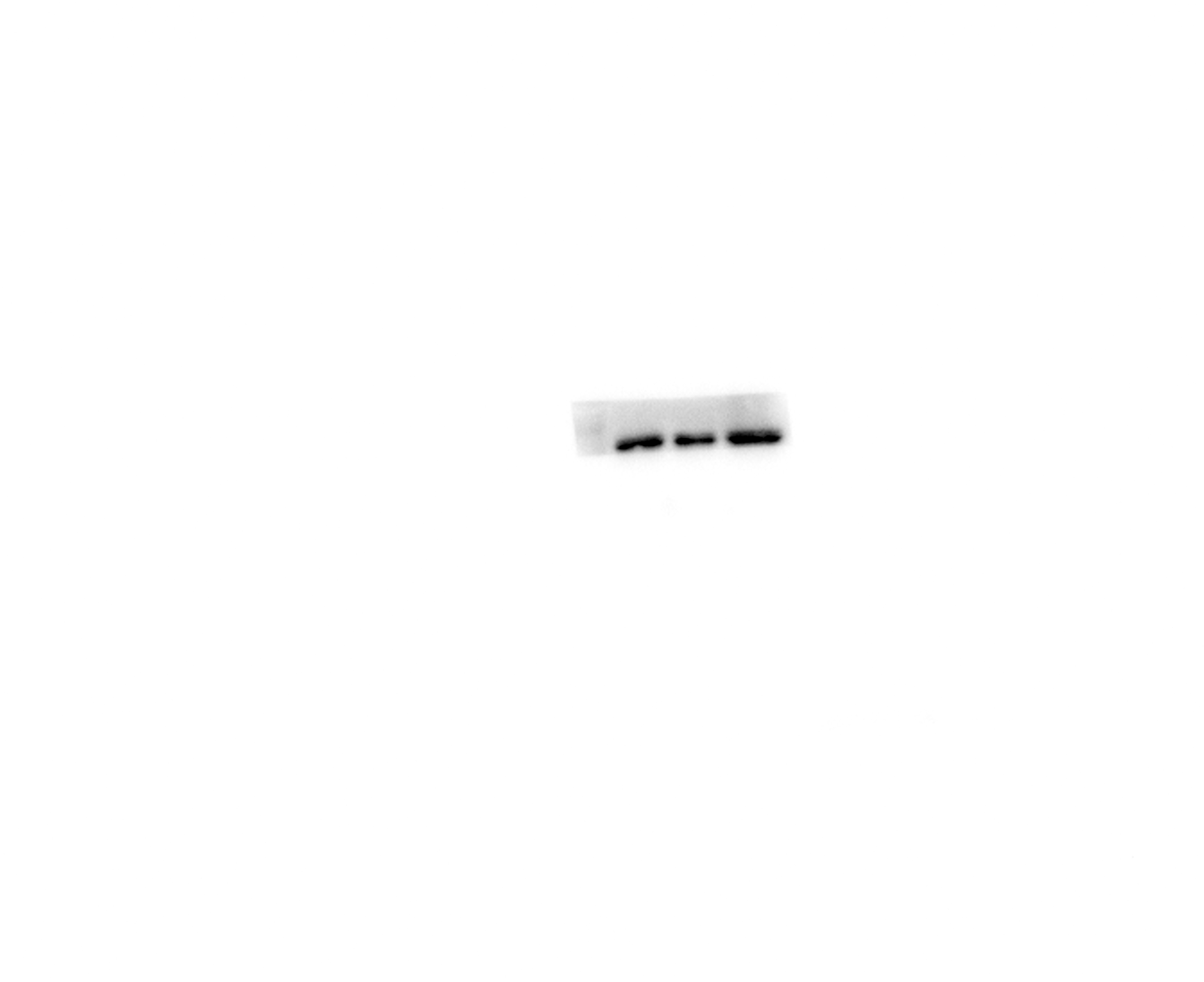** |
|  |  | **ZMAT3** | | **MDM2** | |
|  |  | **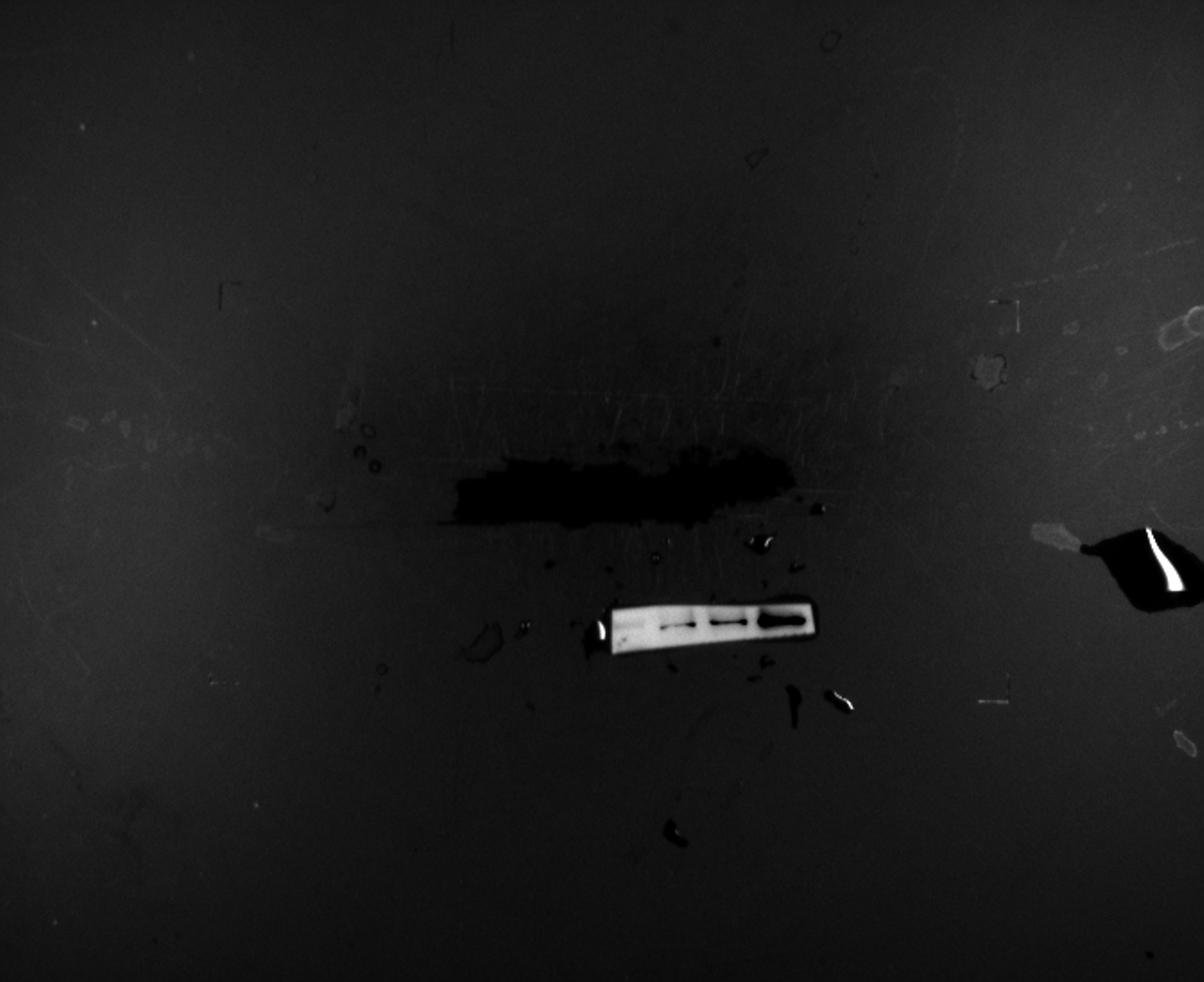** | **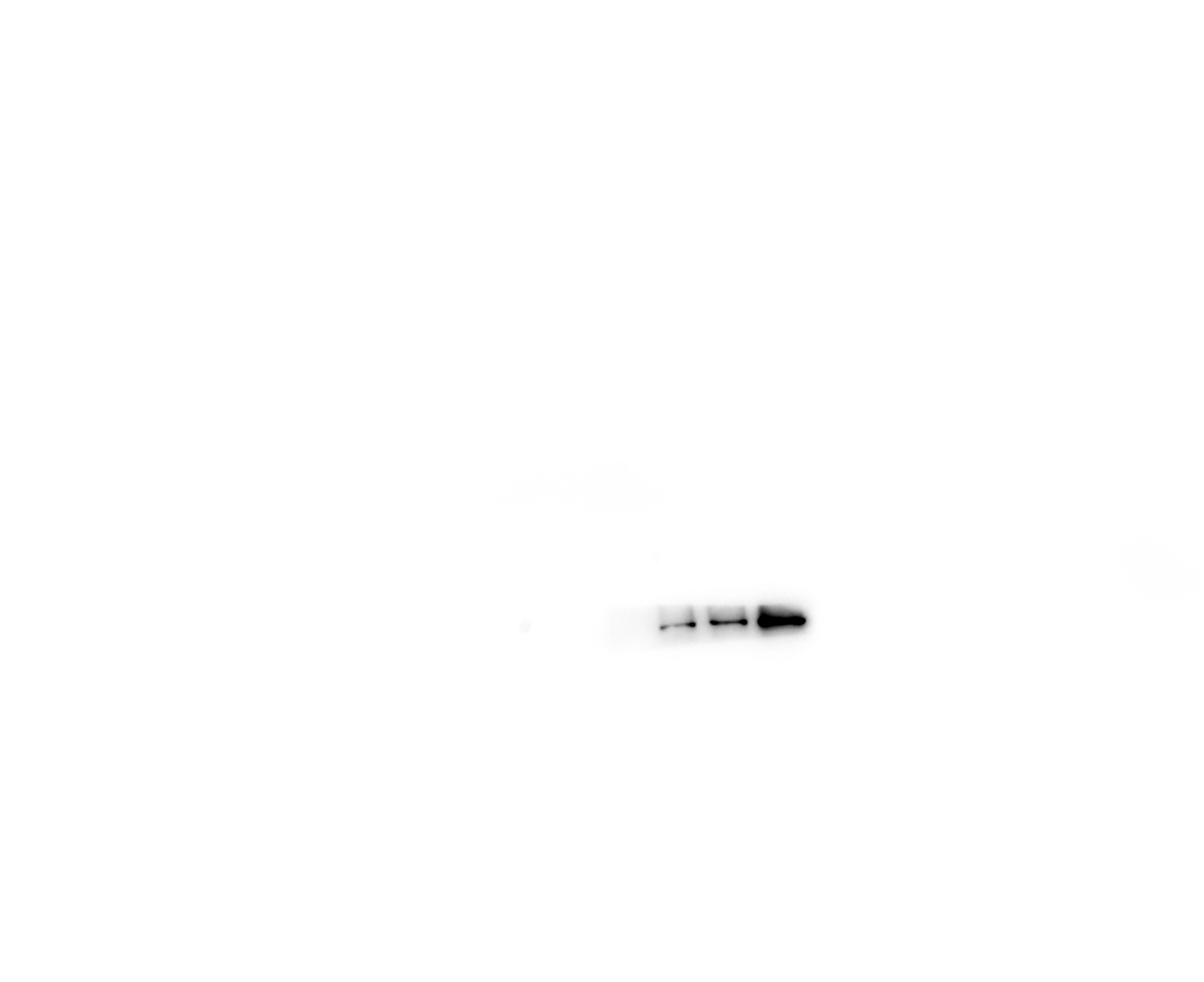** | **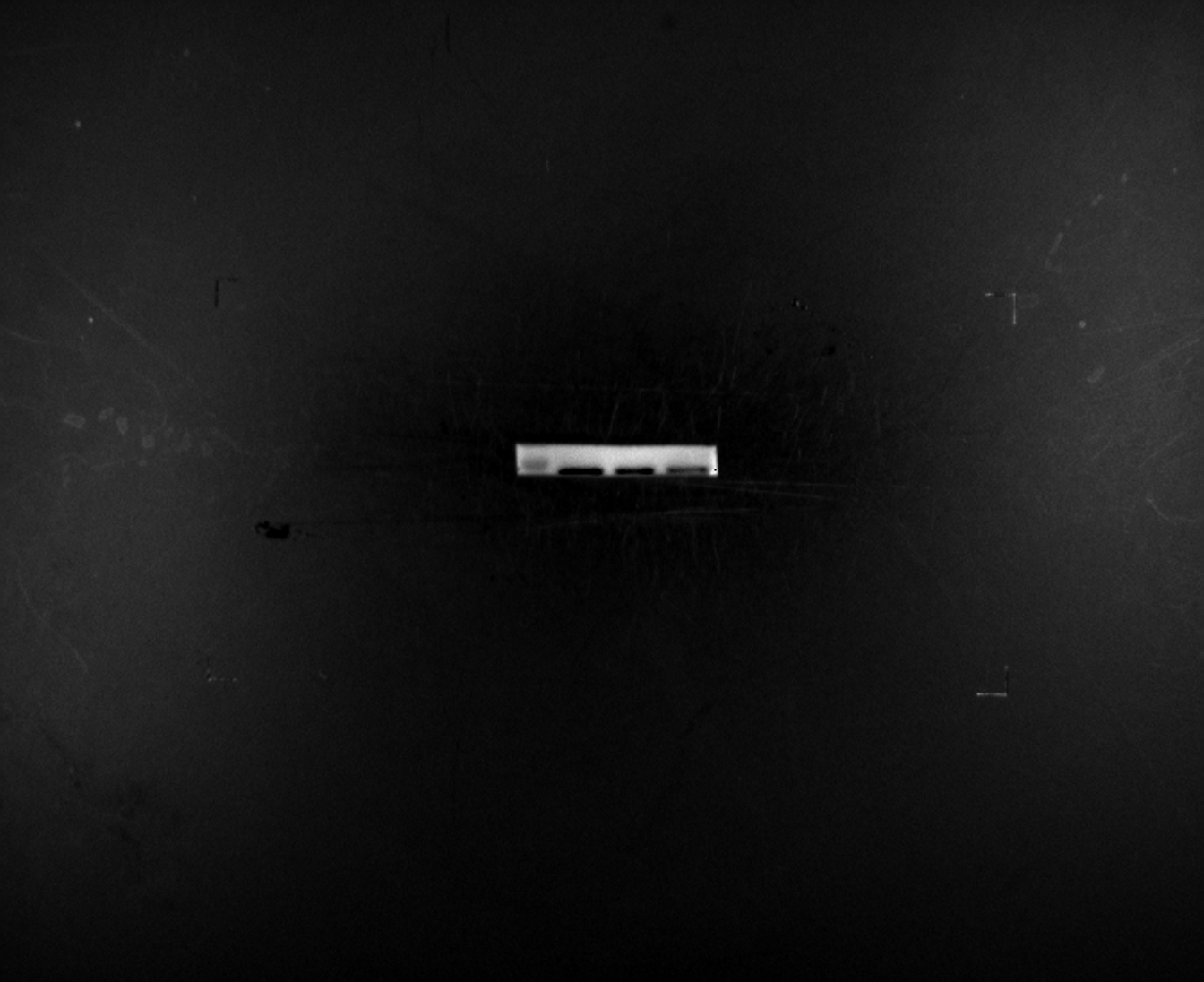** | **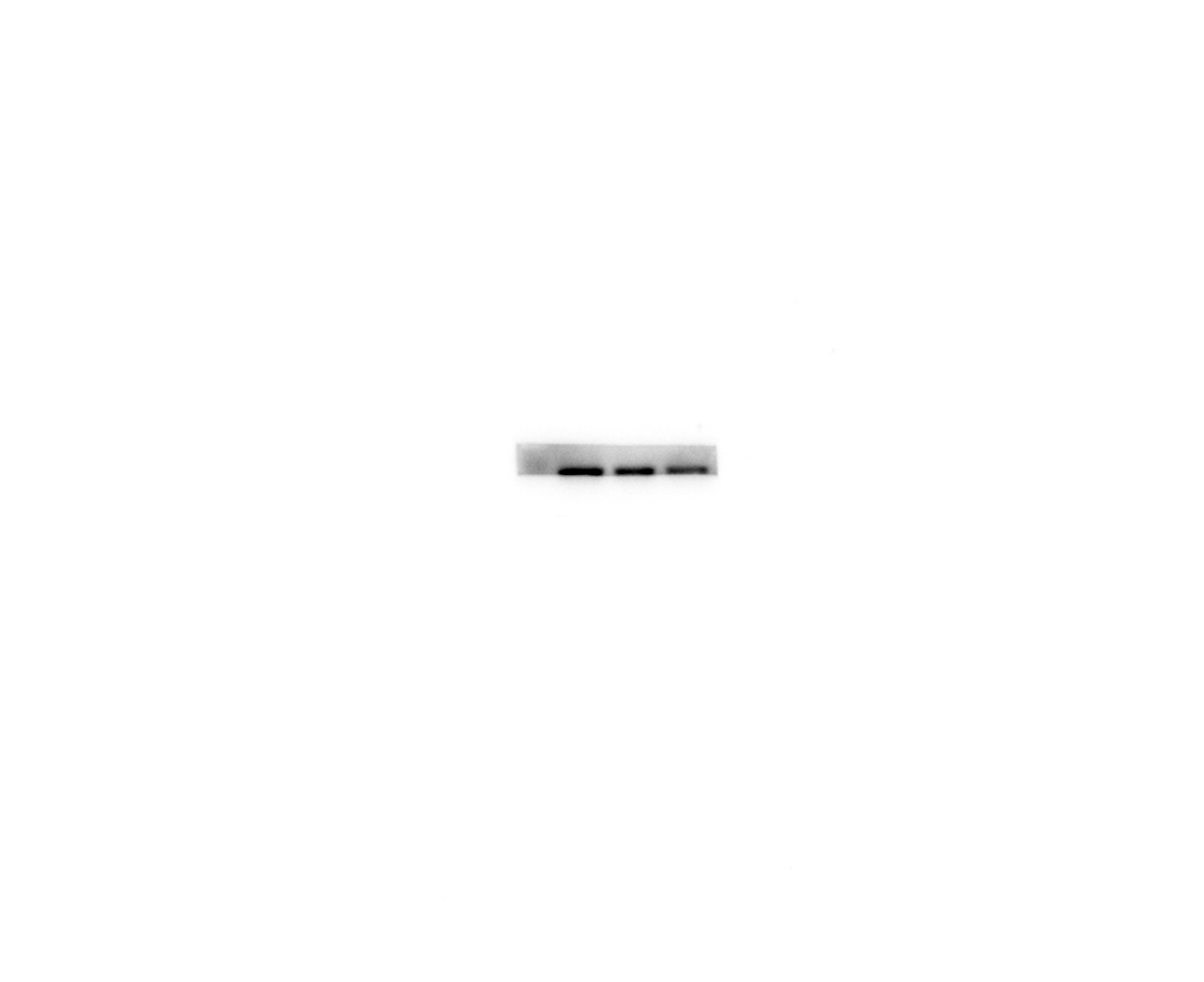** |
|  |  | **SP1** | | **GAPDH** | |
|  |  | **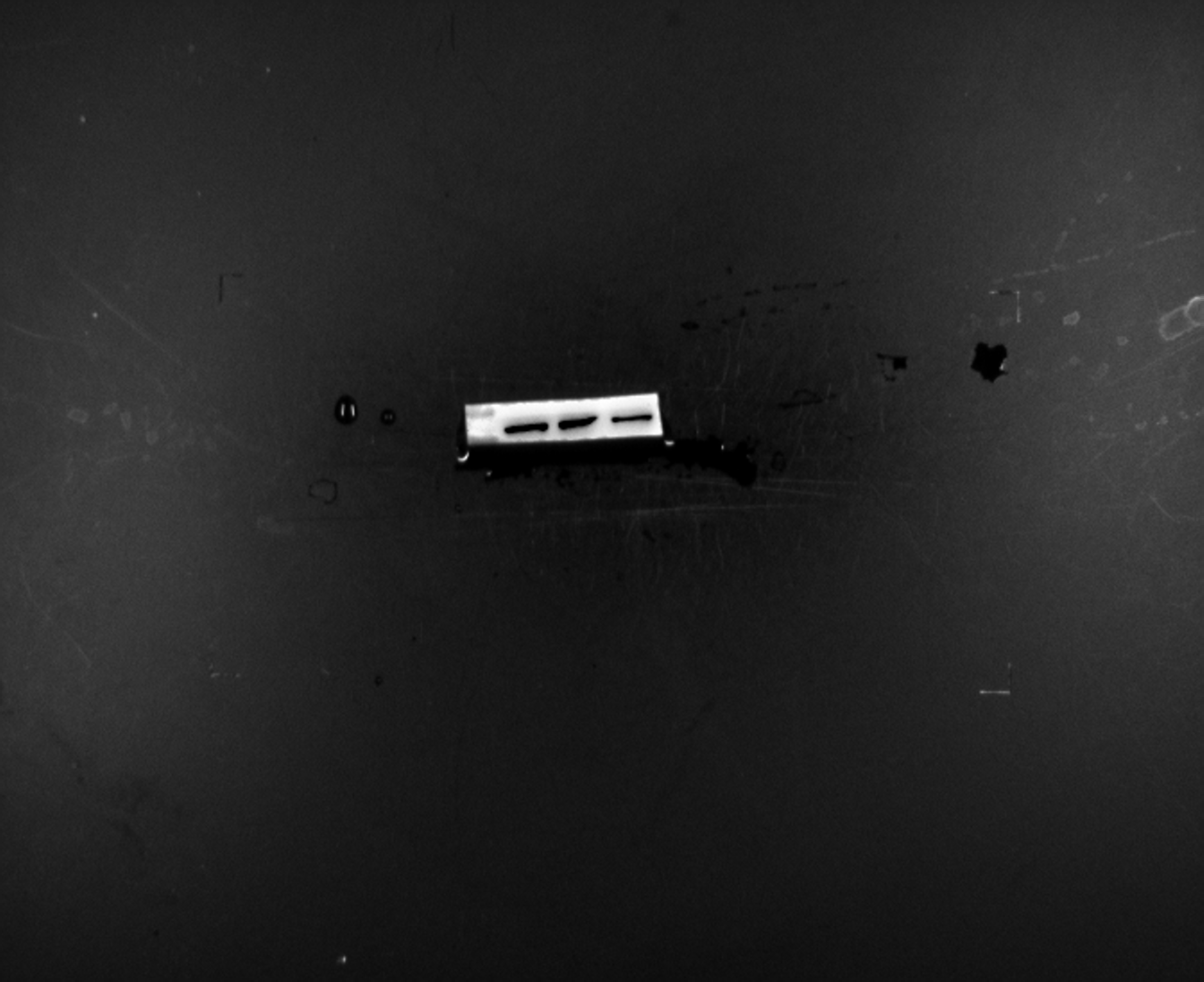** | **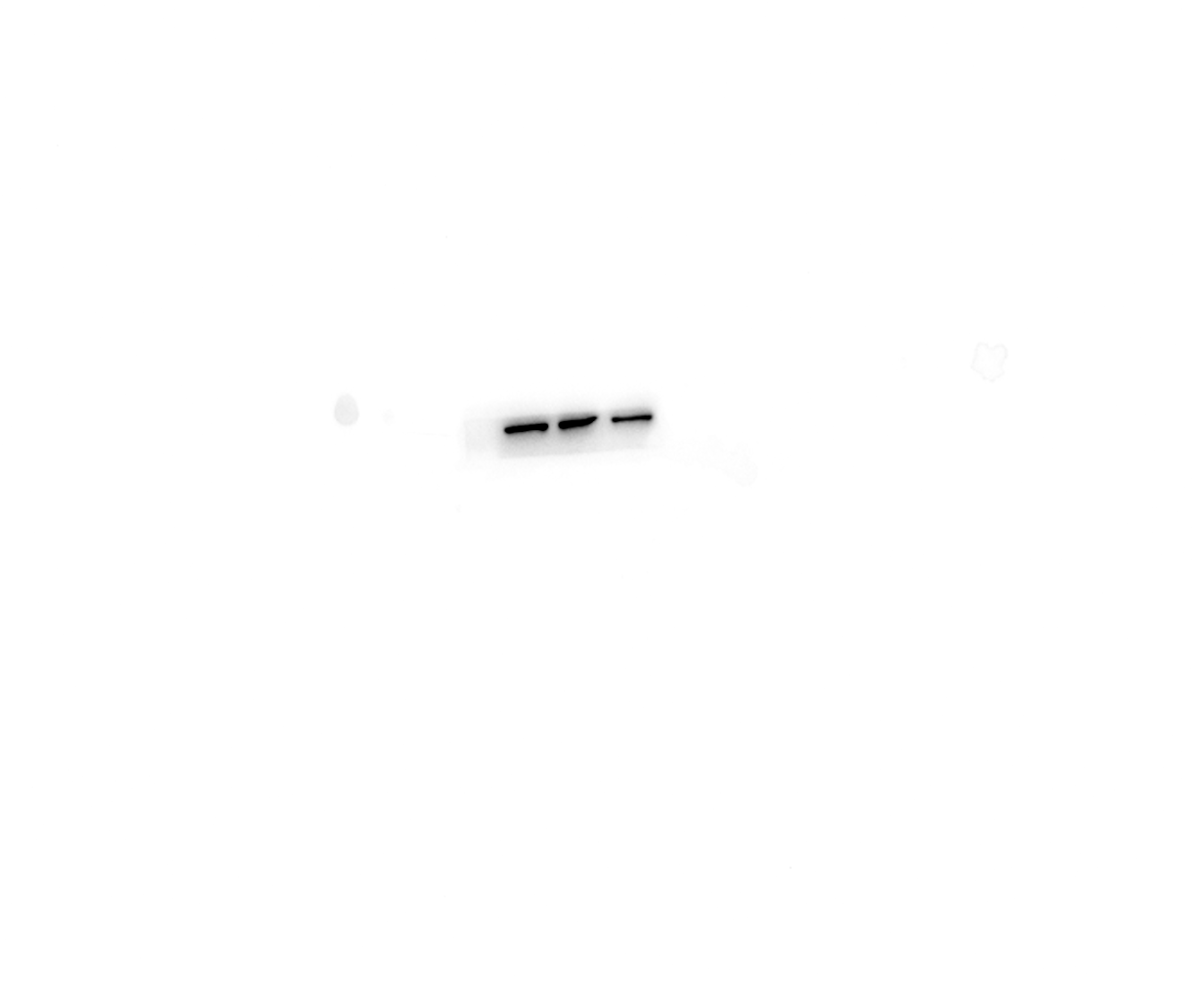** | **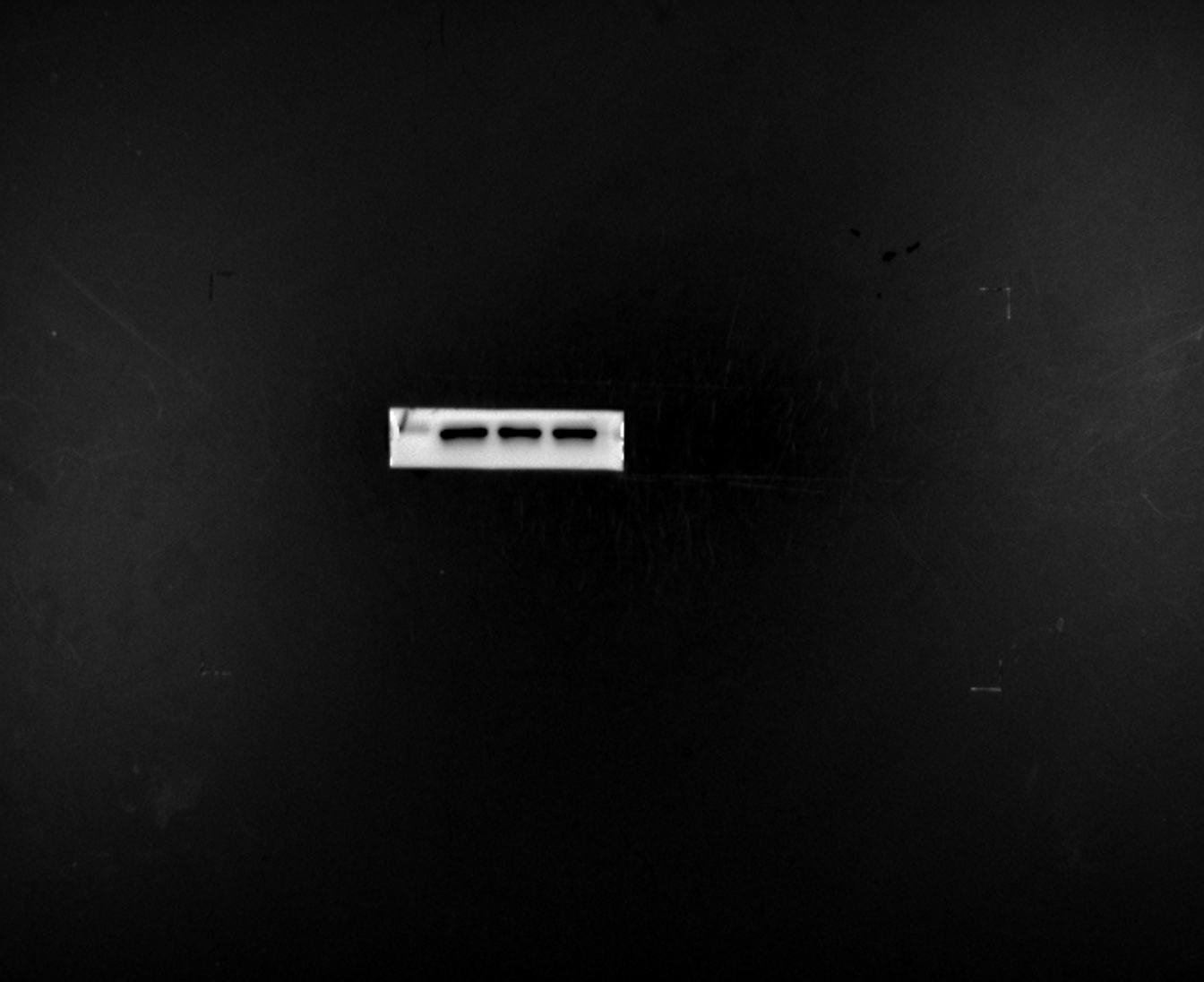** | **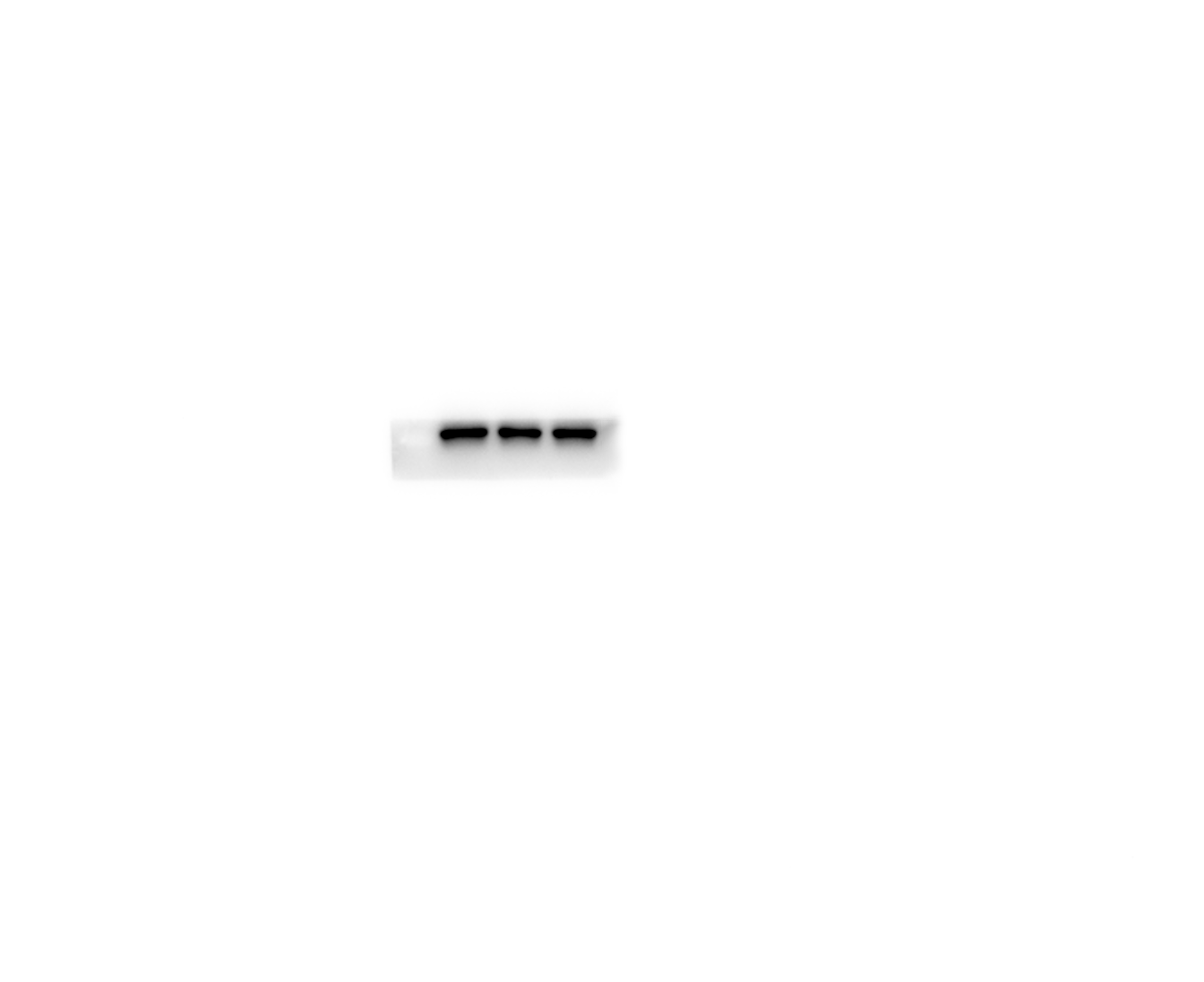** |
| **Fig 4C** | | **p-p53** | | **KLF4** | |
| **HGC-27** | | **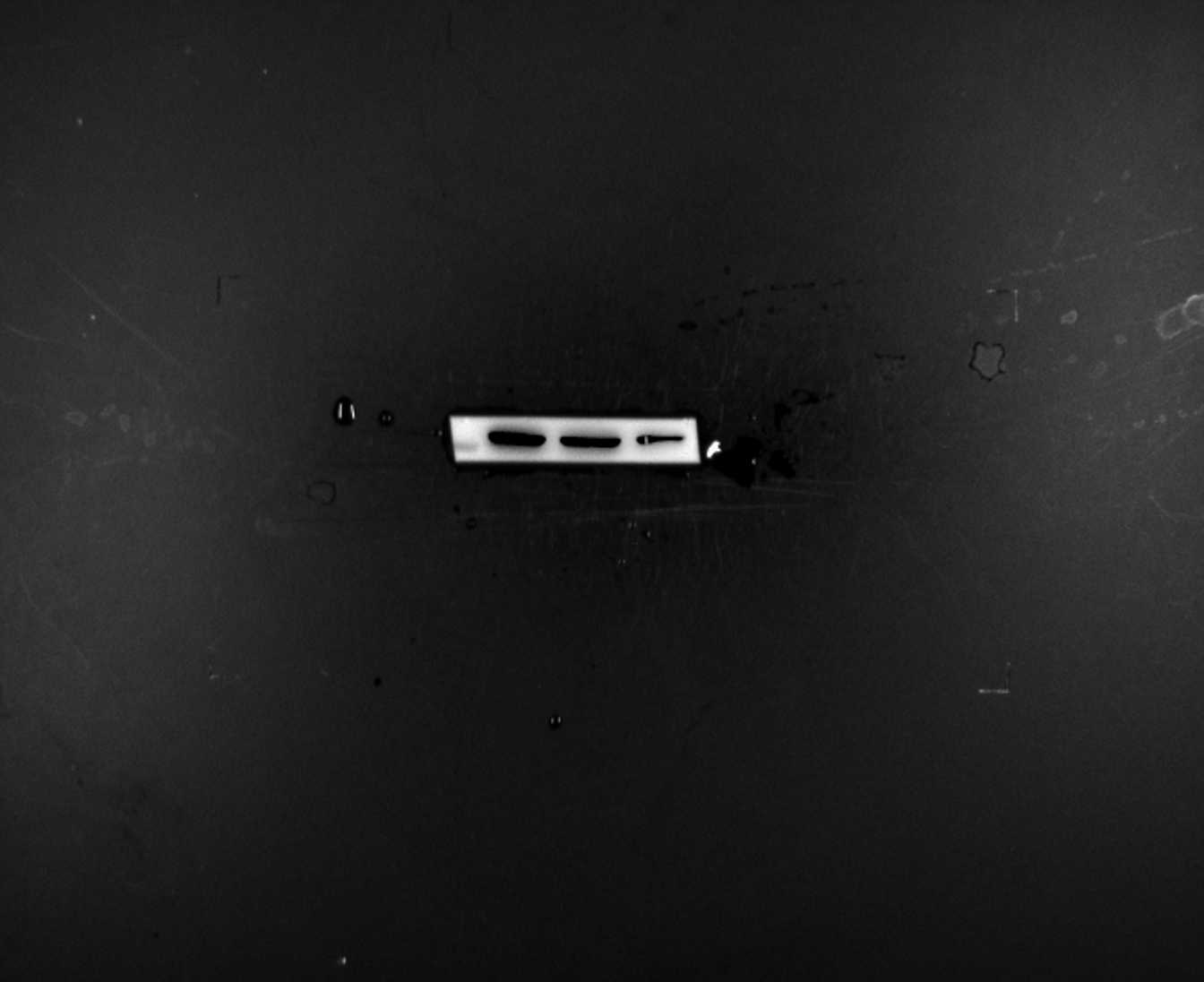** | **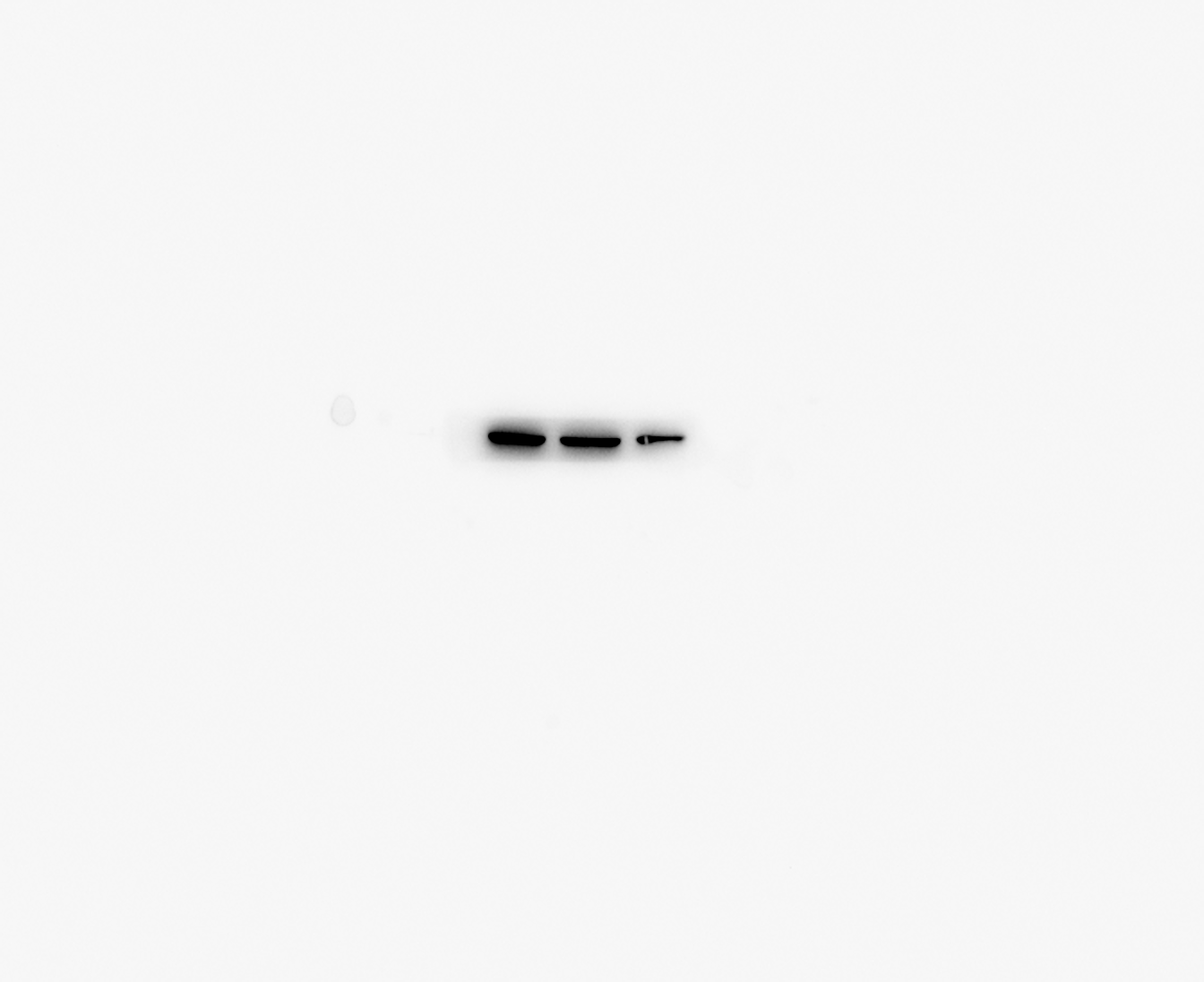** | **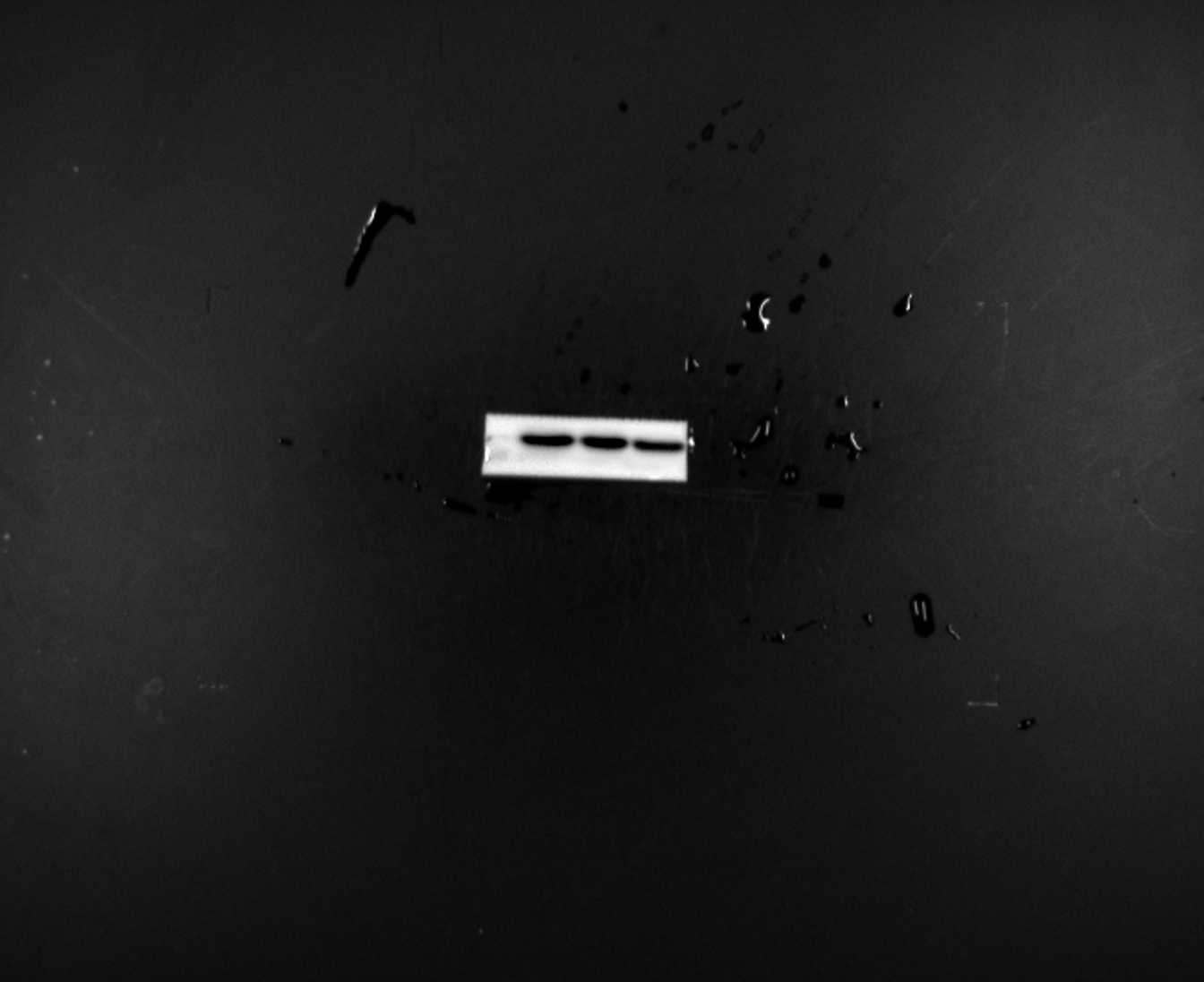** | **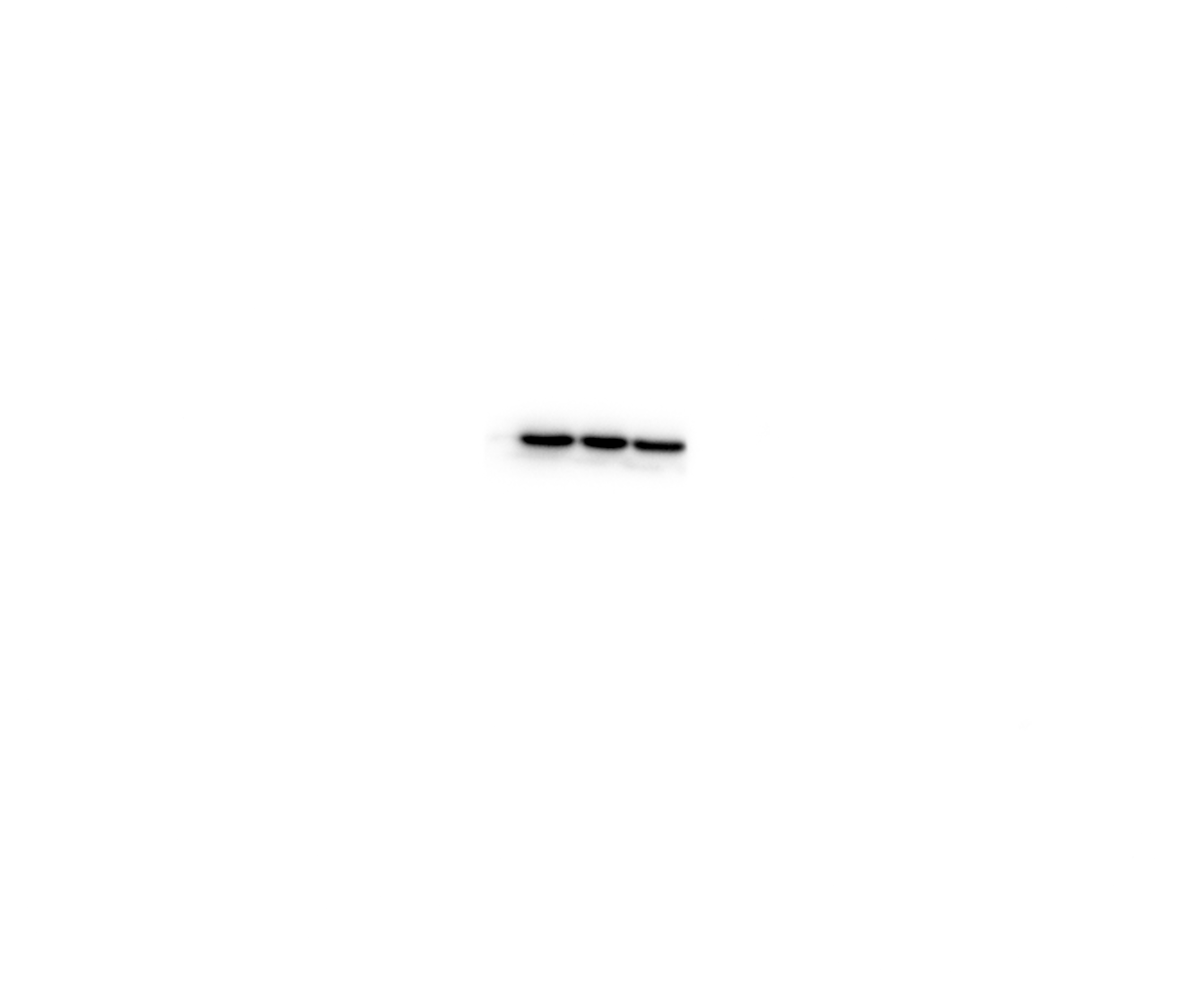** |
|  |  | **ZMAT3** | | **MDM2** | |
|  |  | **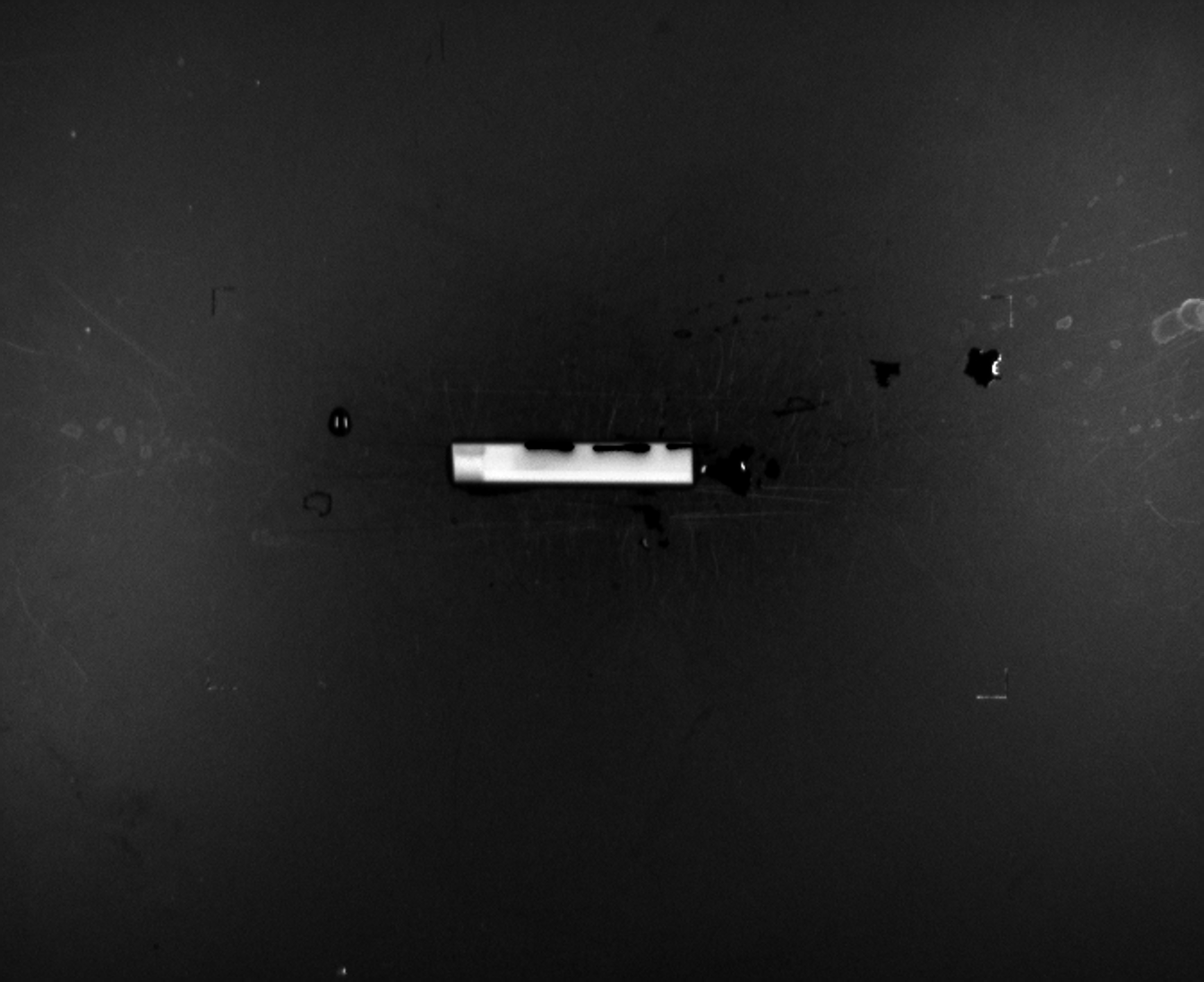** | **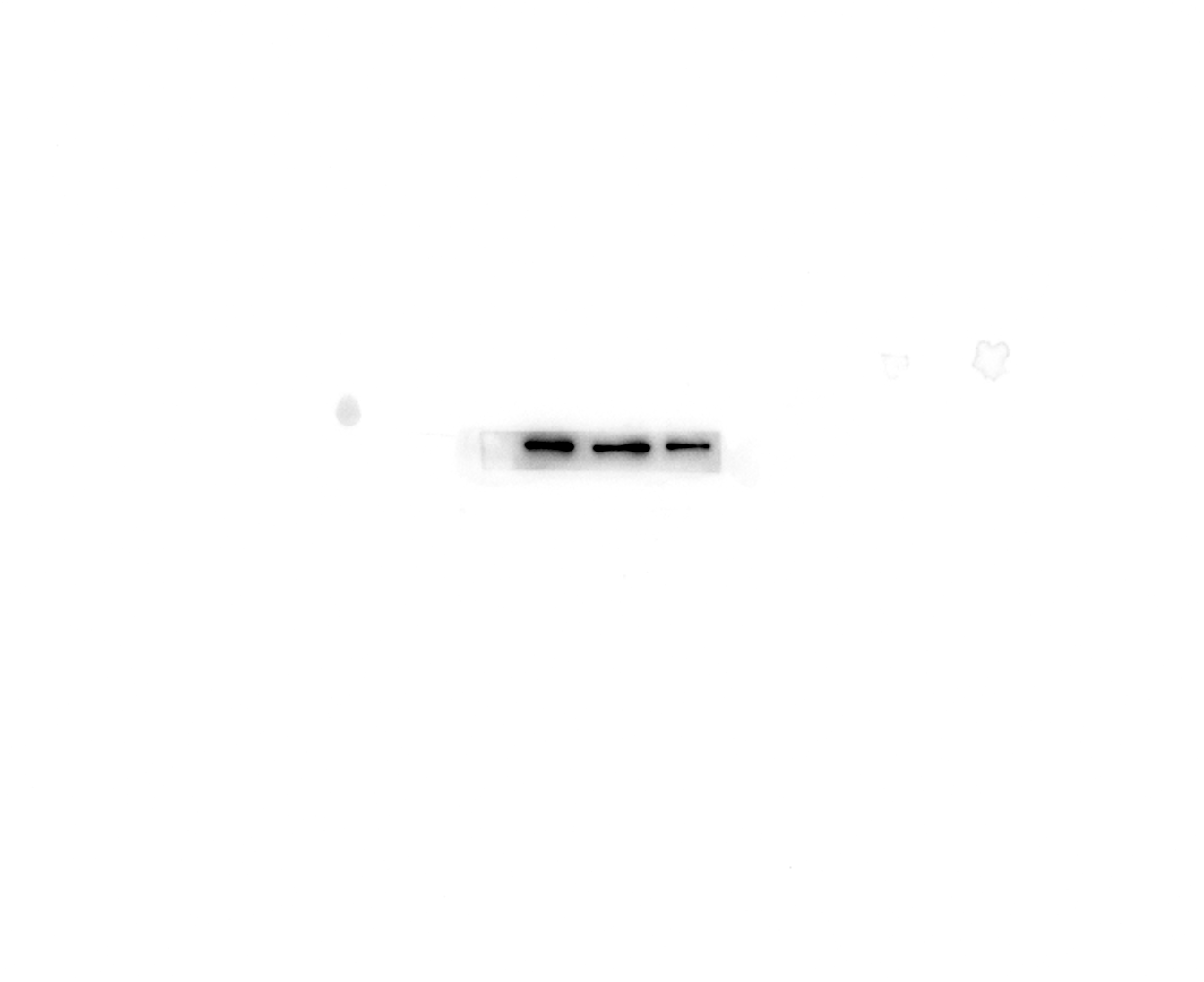** | **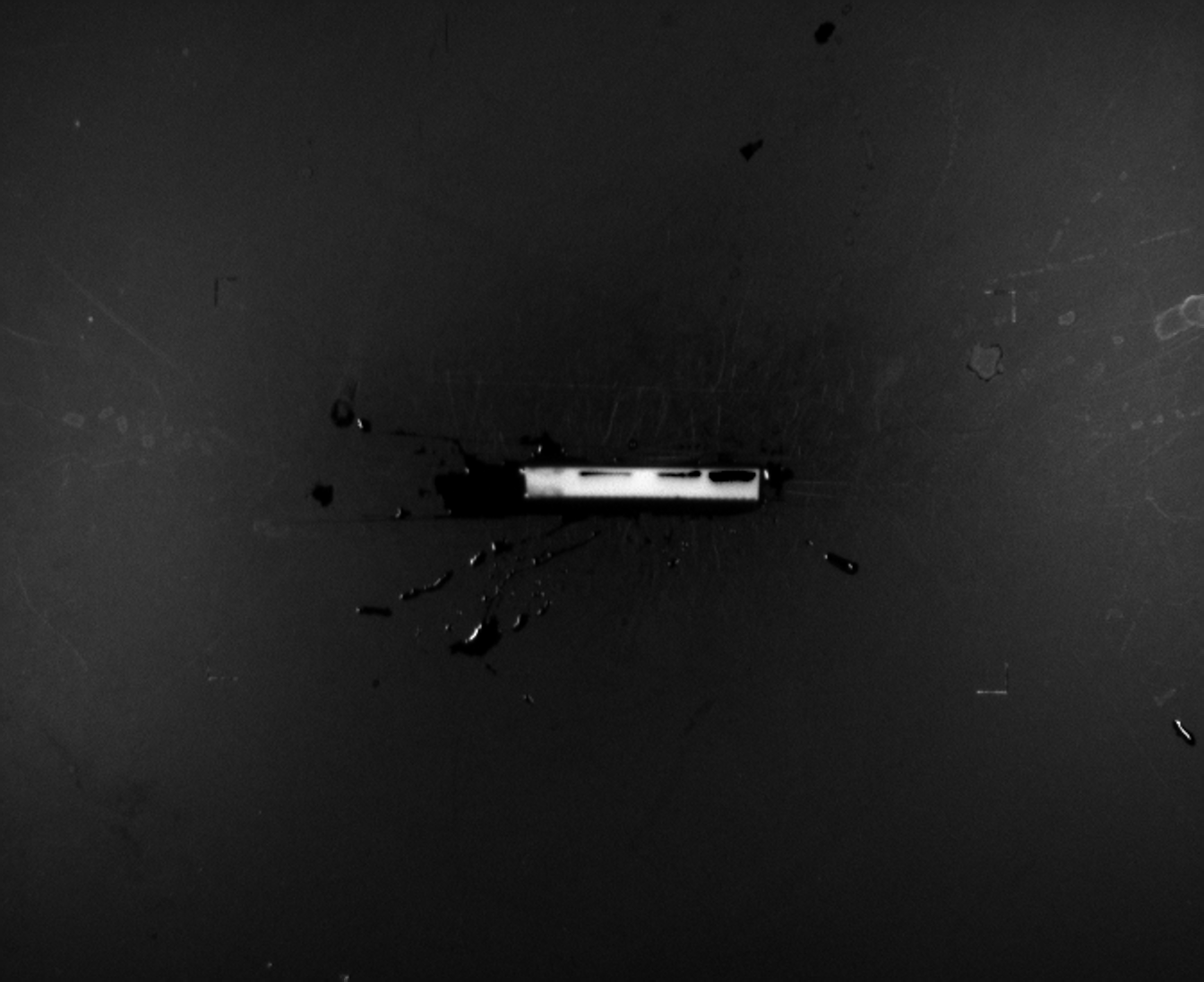** | **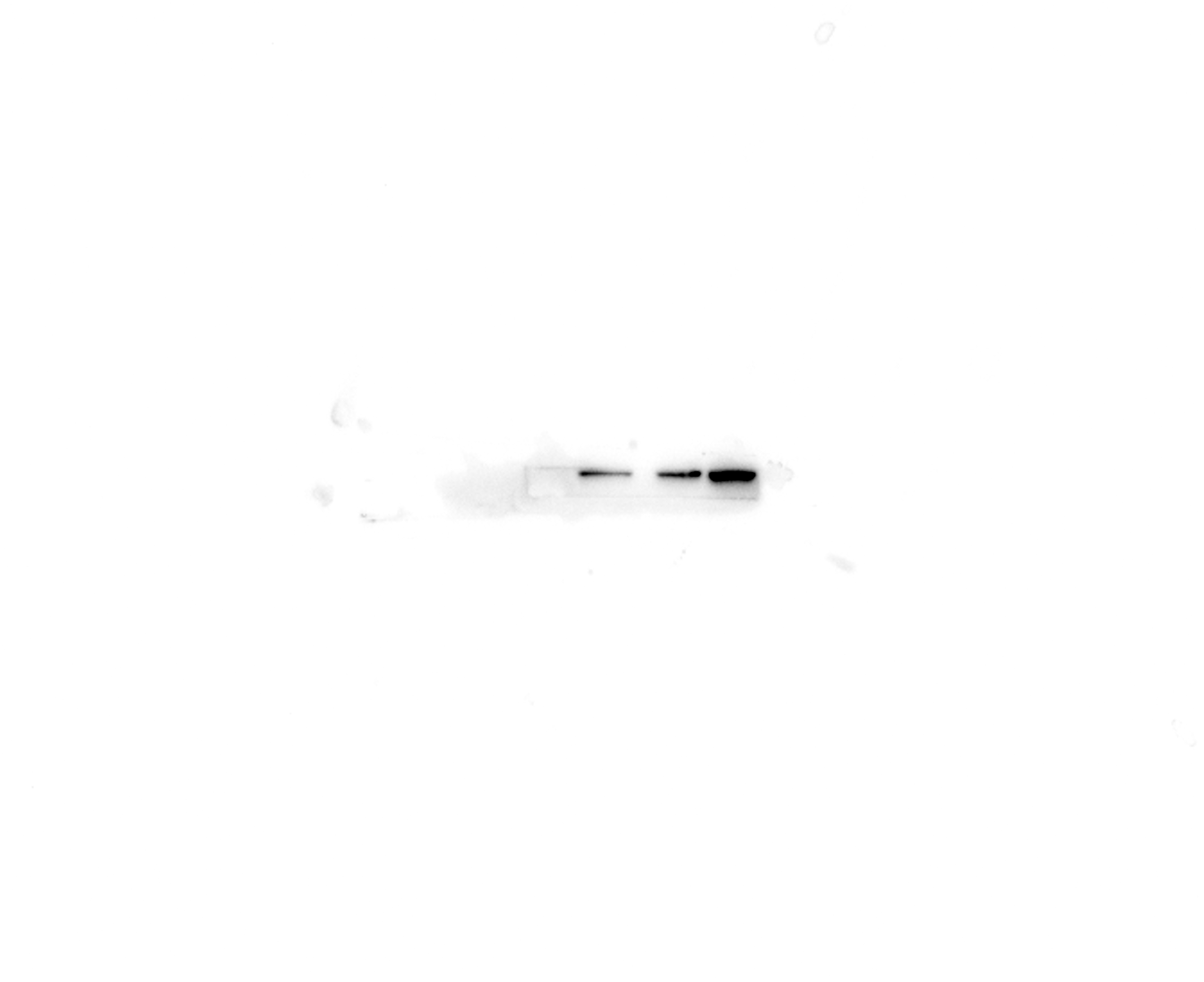** |
|  |  | **SP1** | | **GAPDH** | |
|  |  | **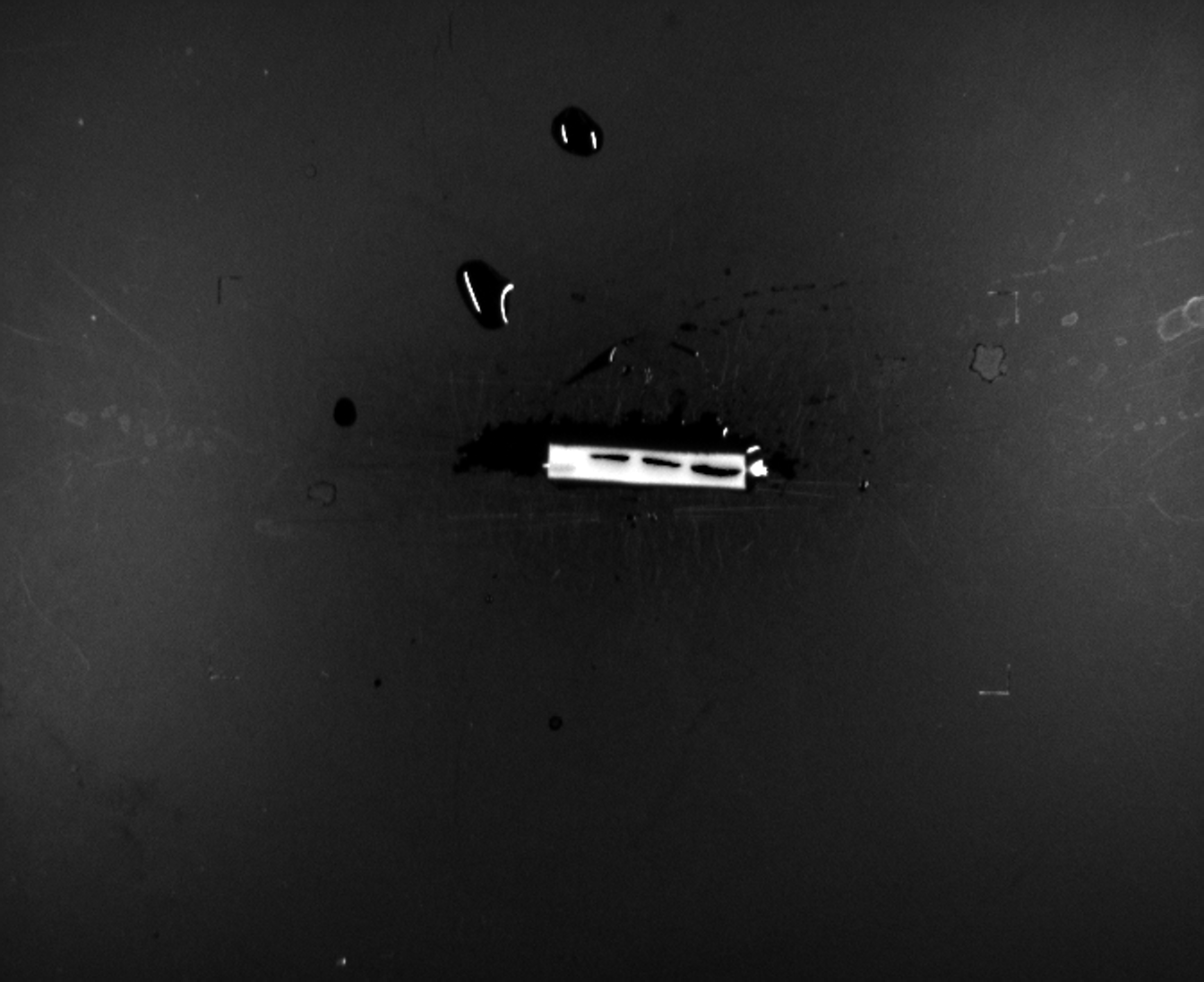** | **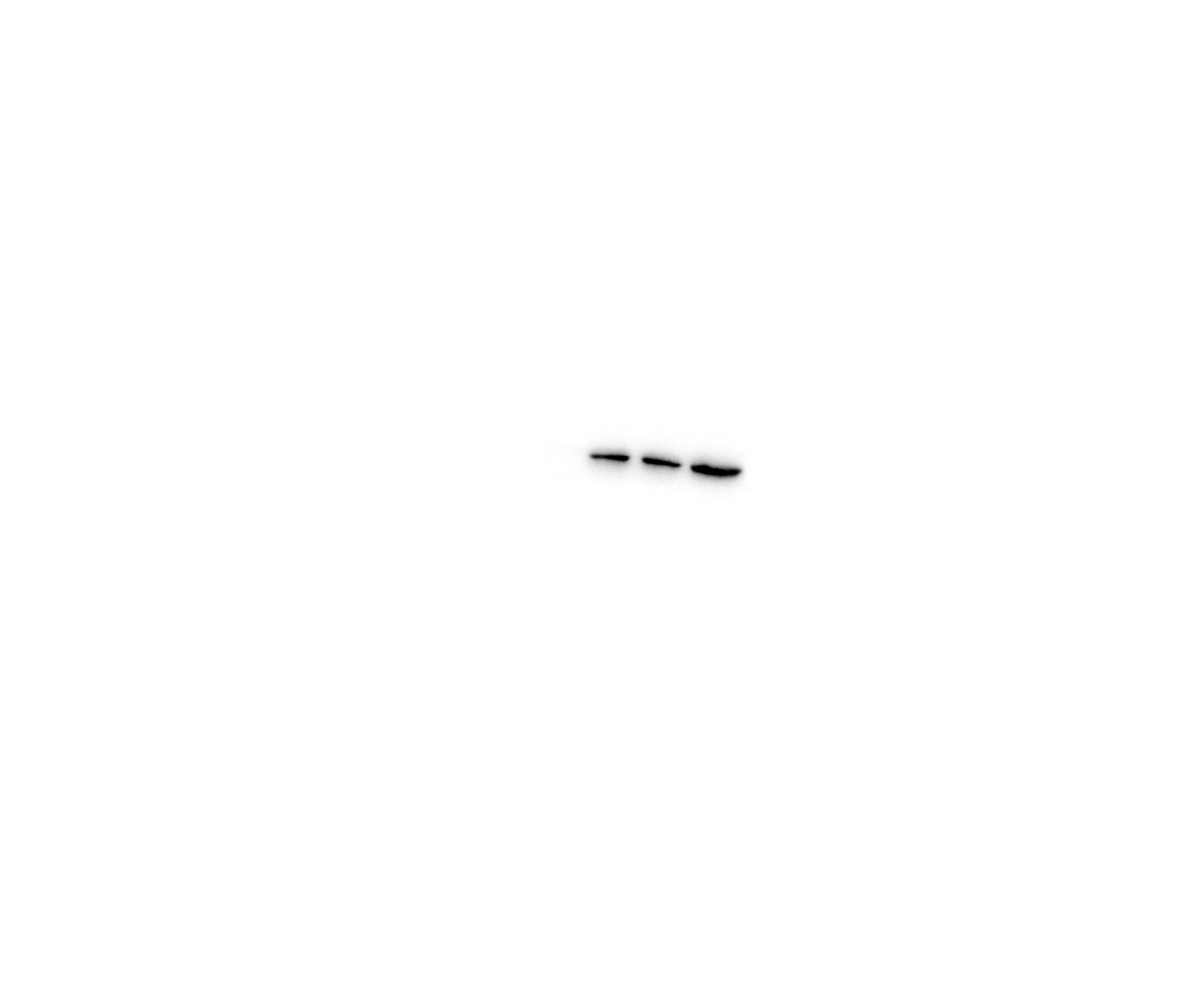** | **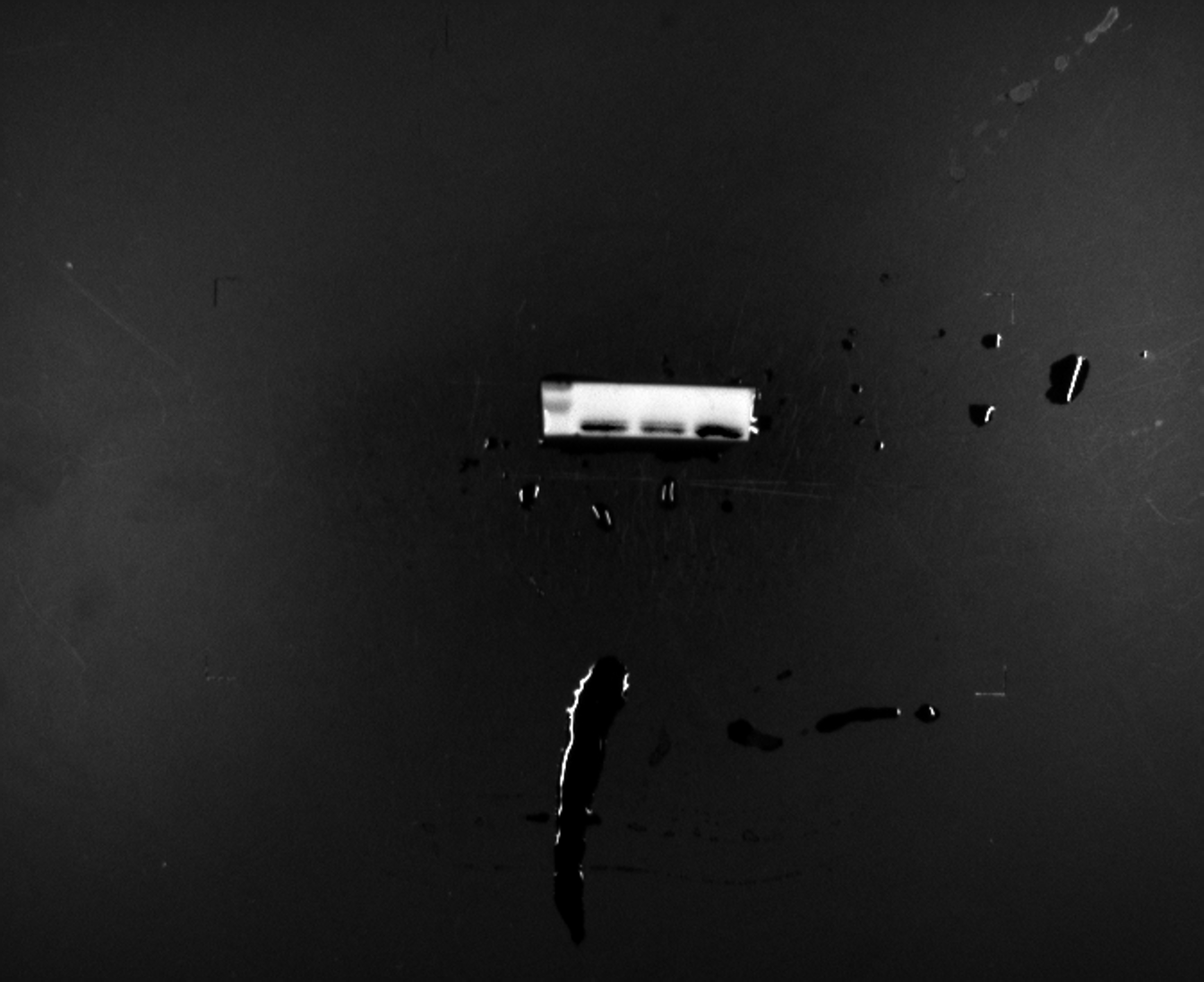** | **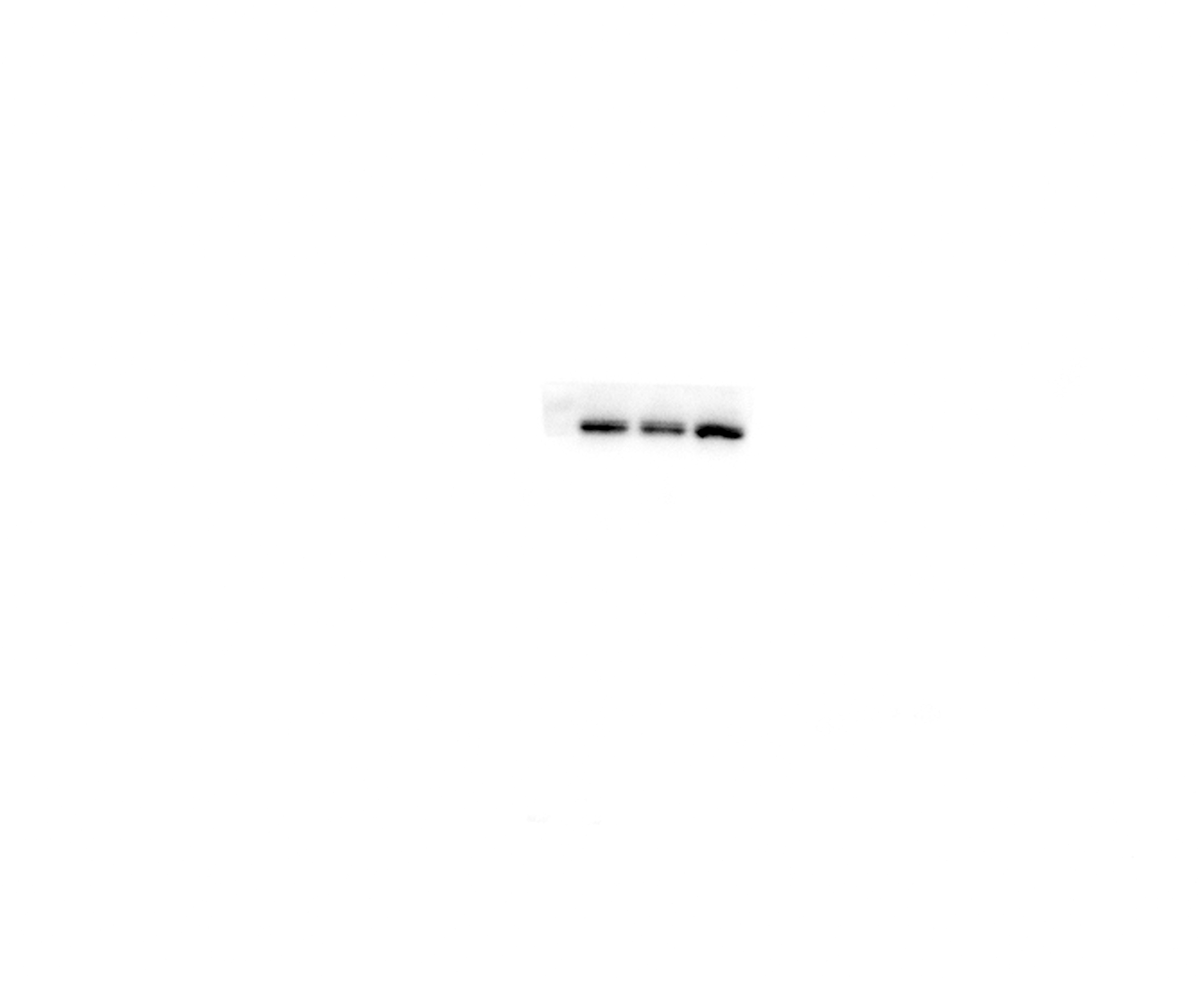** |
| **Fig 5A** | | **UPK3A** | | **GAPDH** | |
| **SNU-216** | | **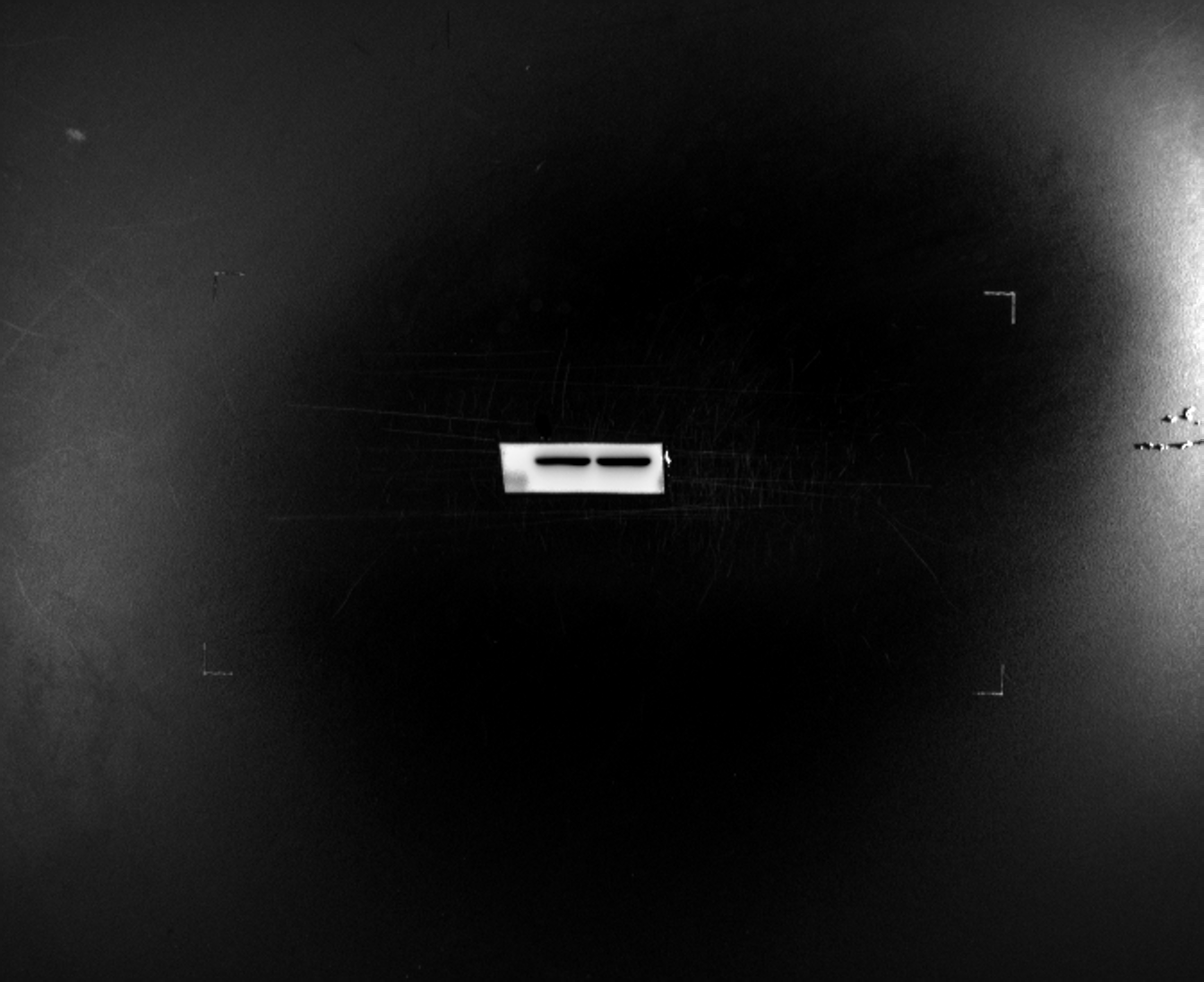** | **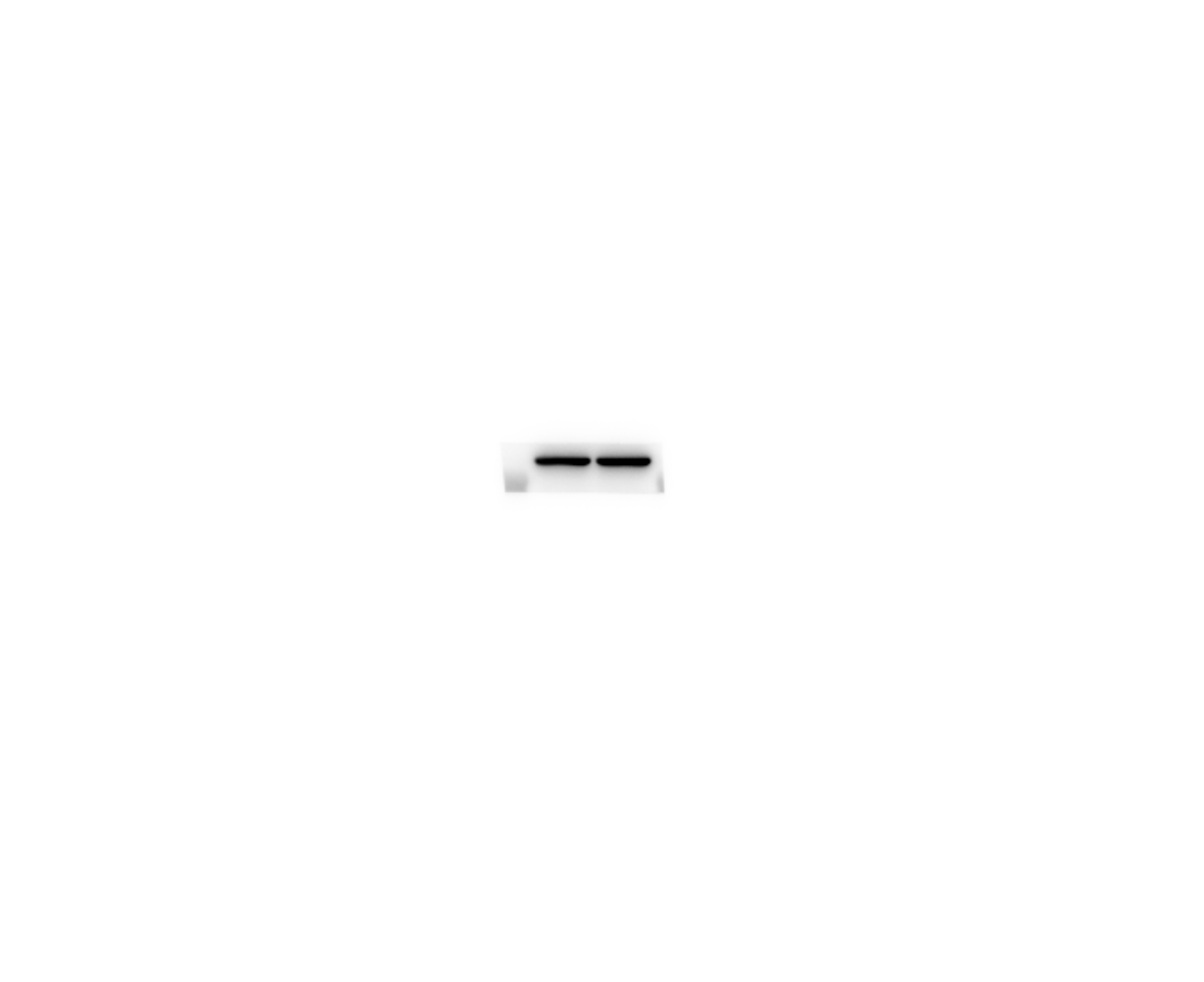** | **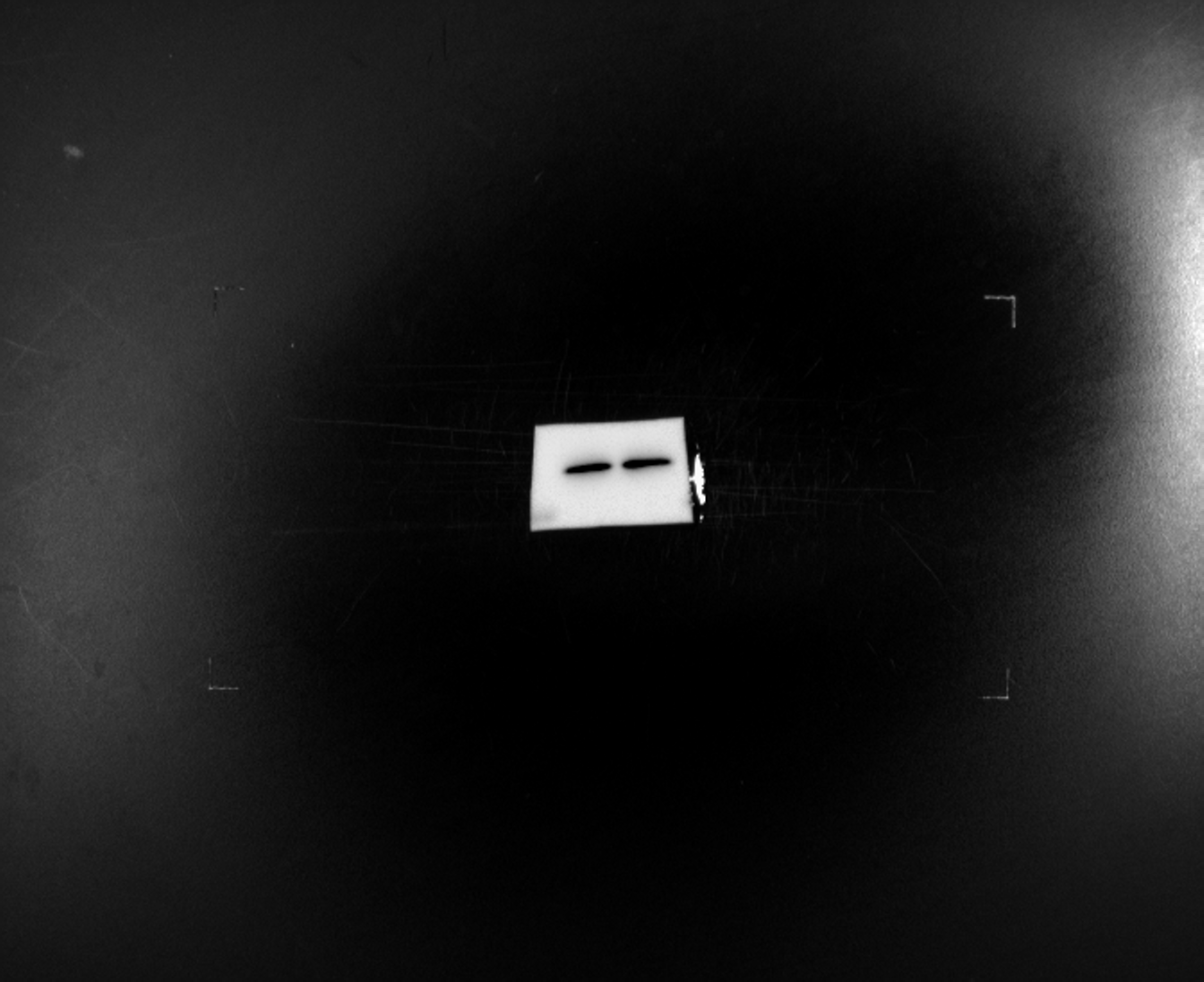** | **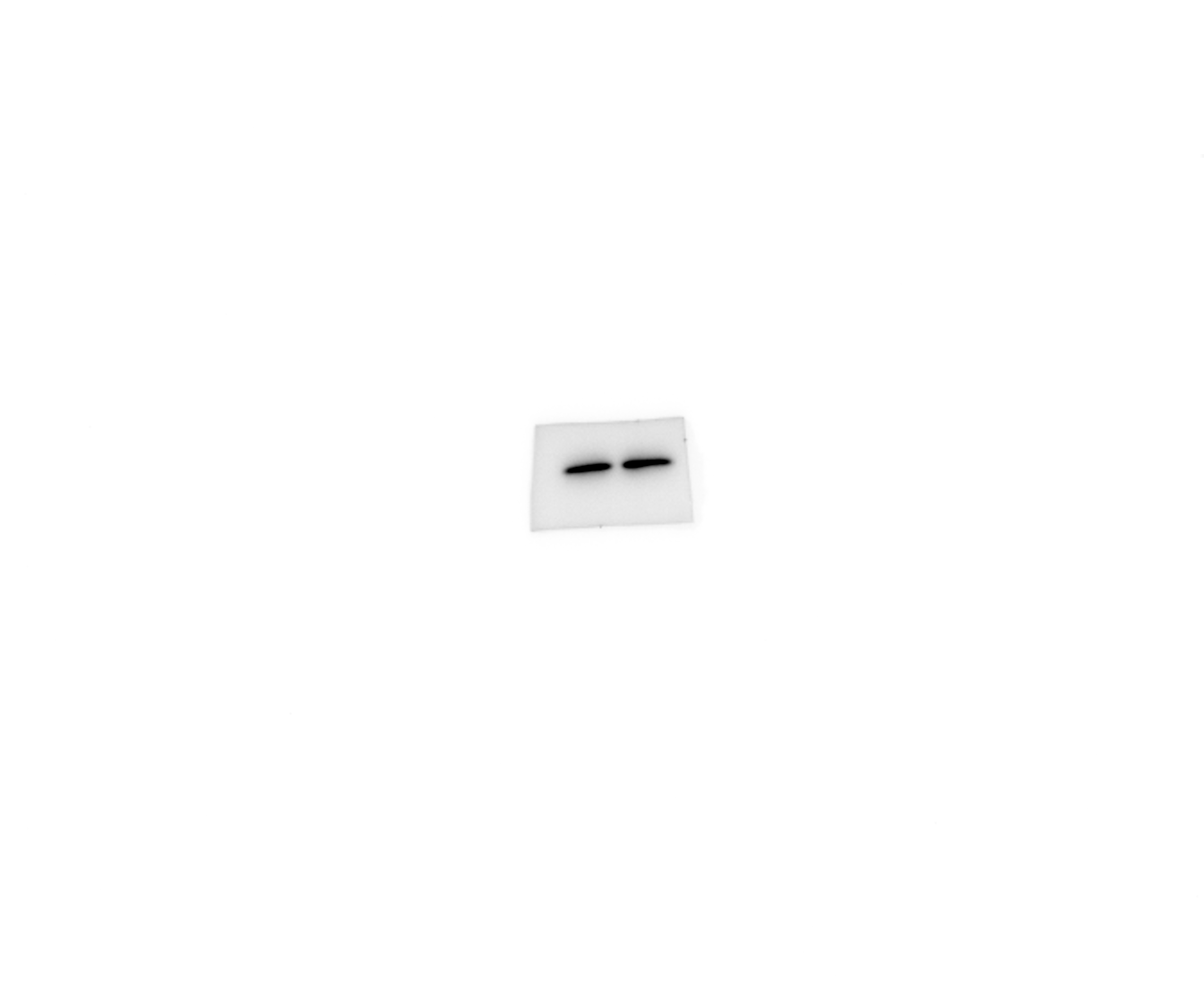** |
| **HGC-27** | | **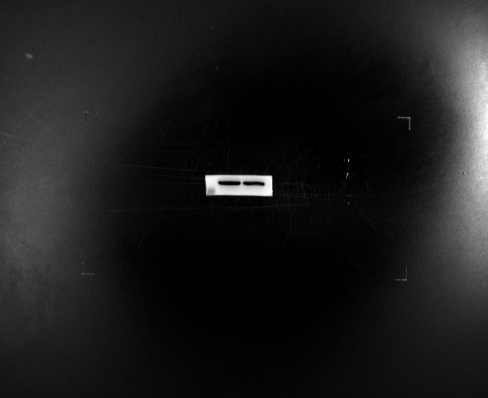** | **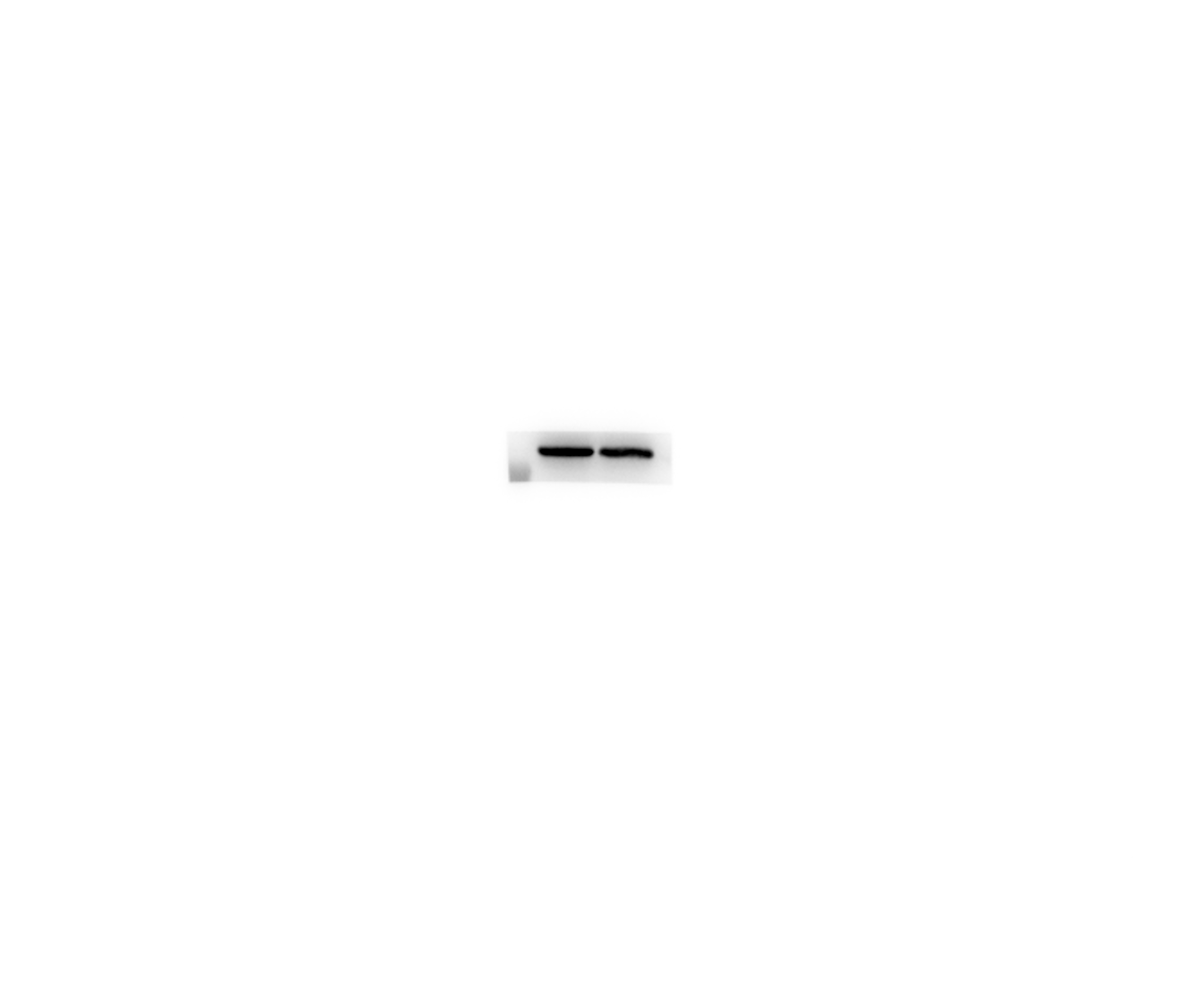** | **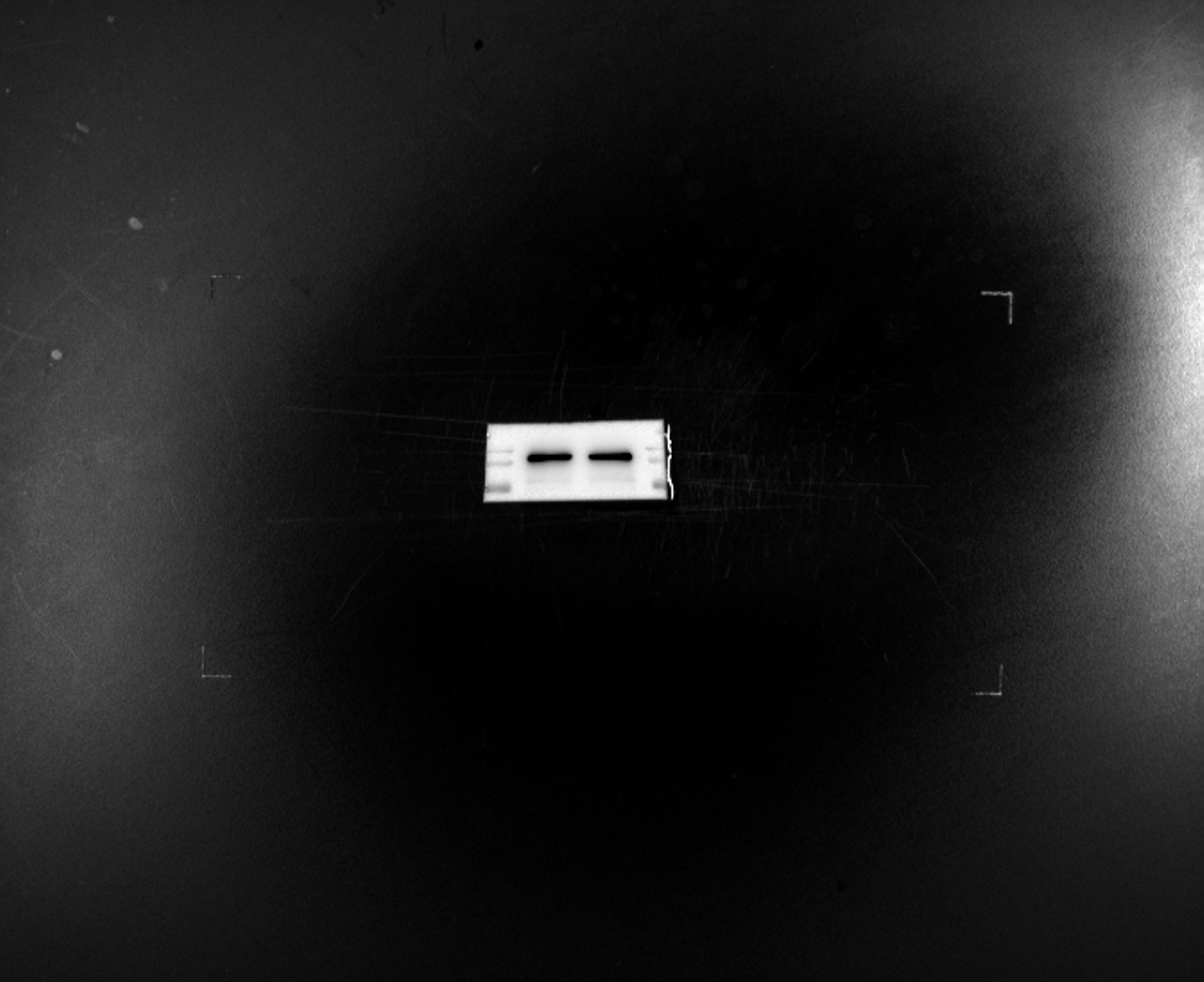** | **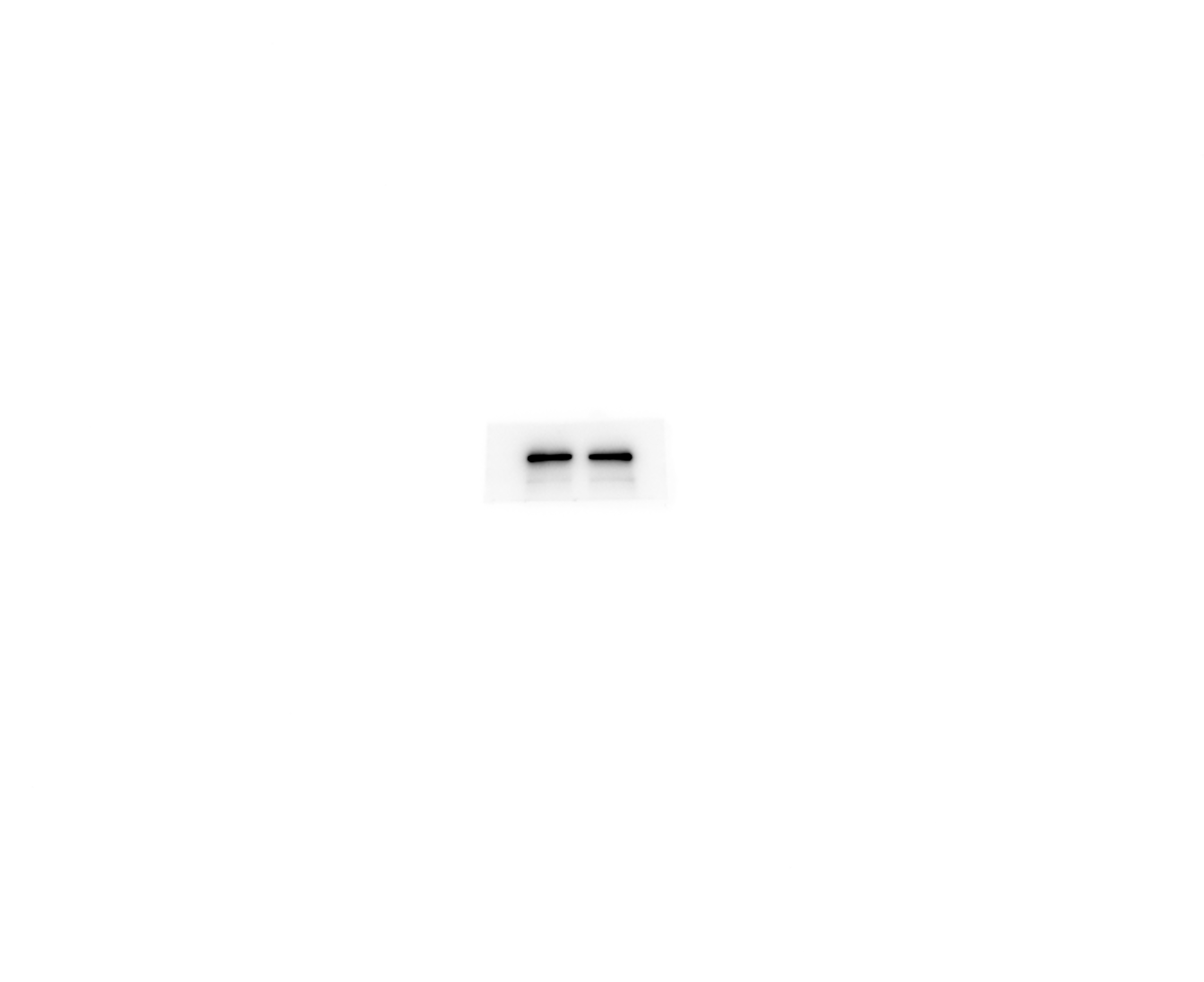** |
| **Fig 5B** | | **p-p53** | | **KLF4** | |
| **SNU-216** | | **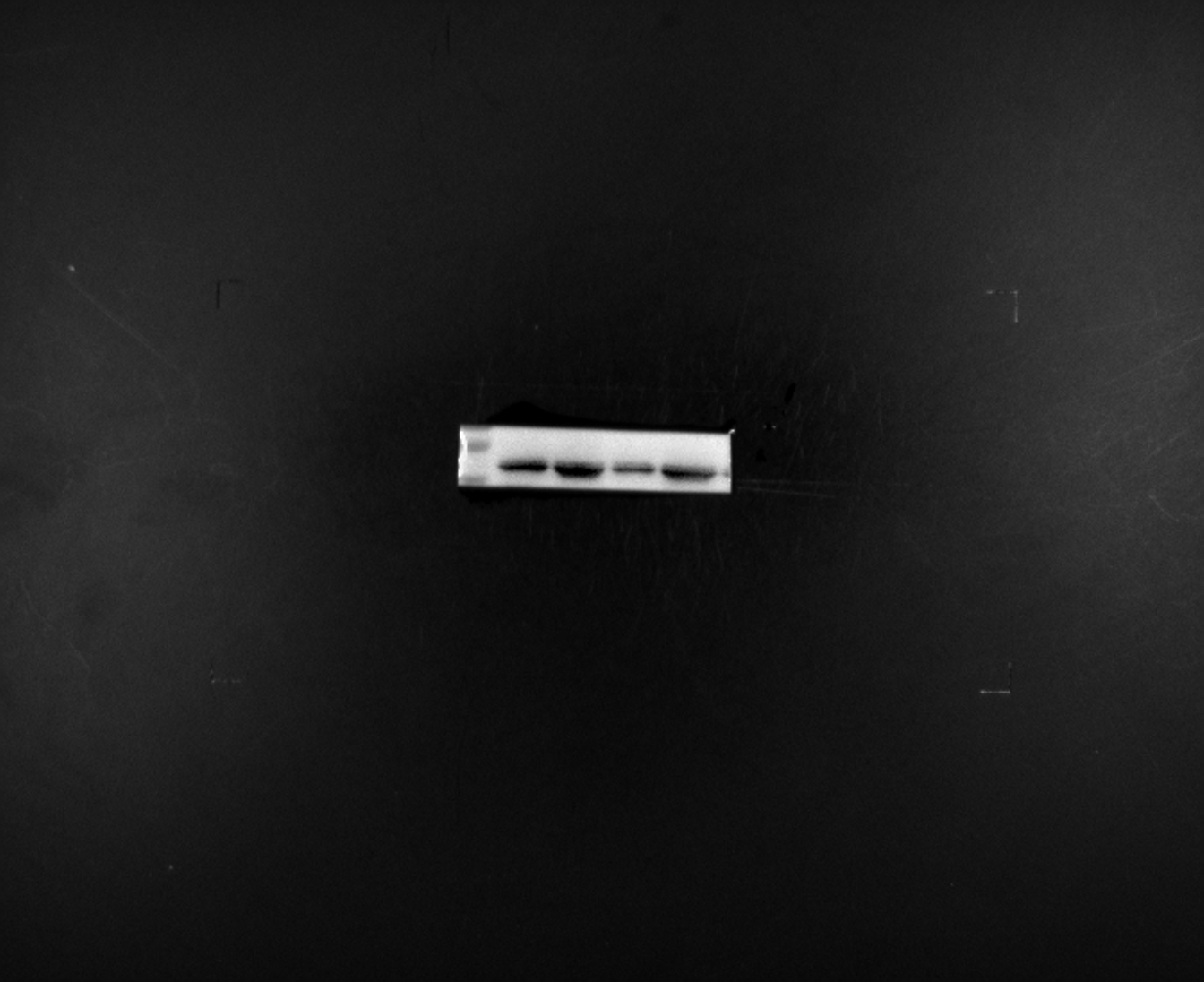** | **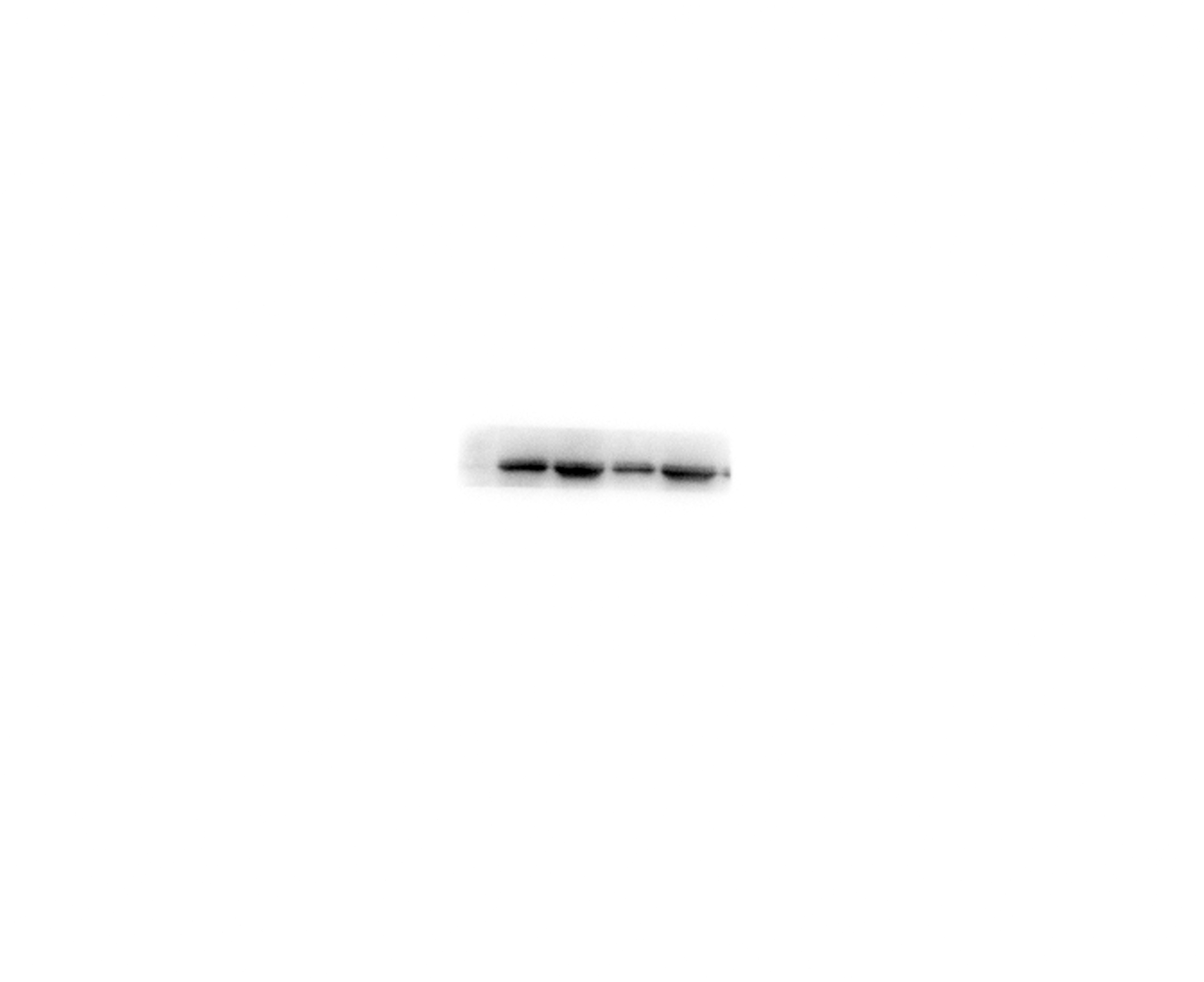** | **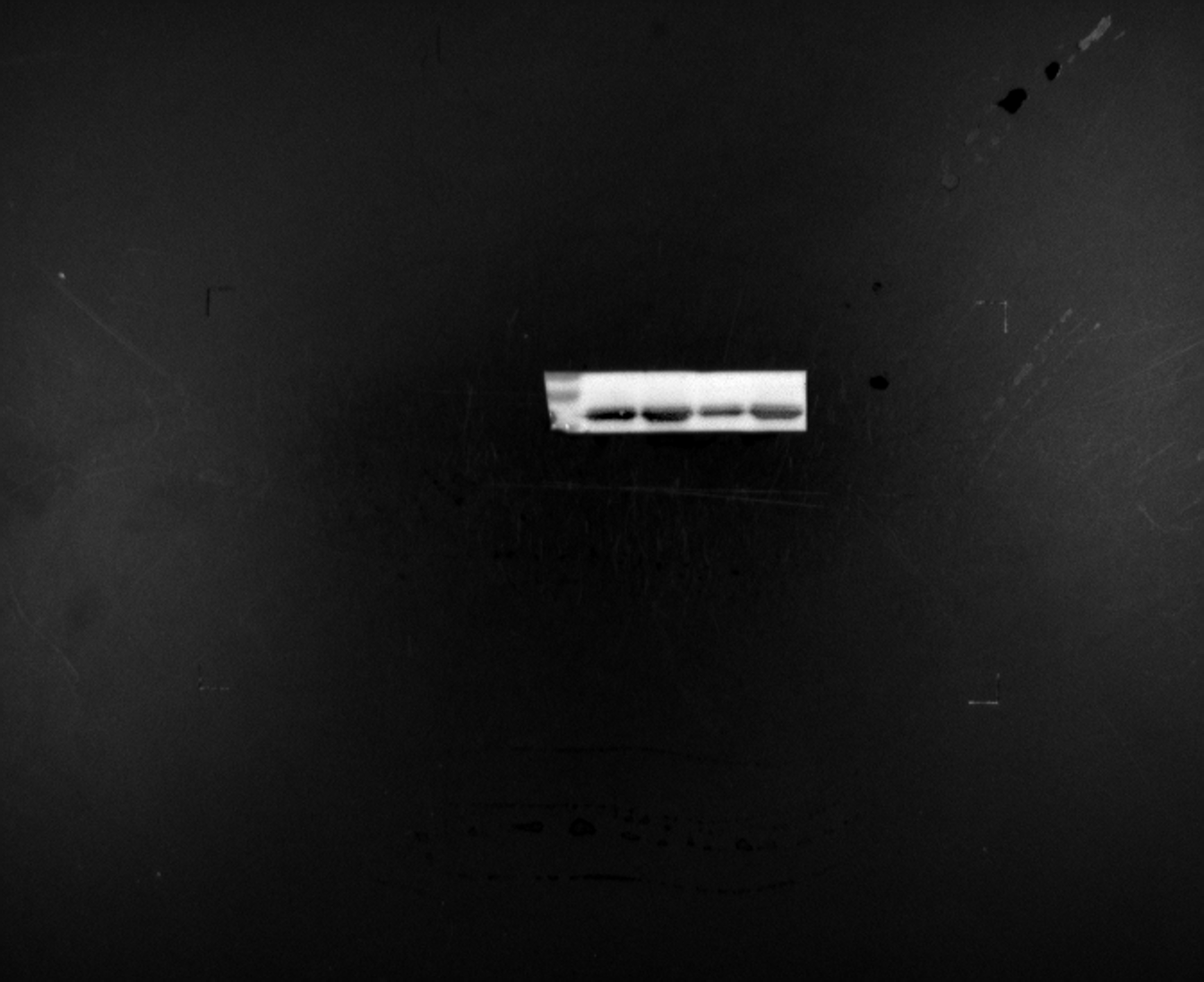** | **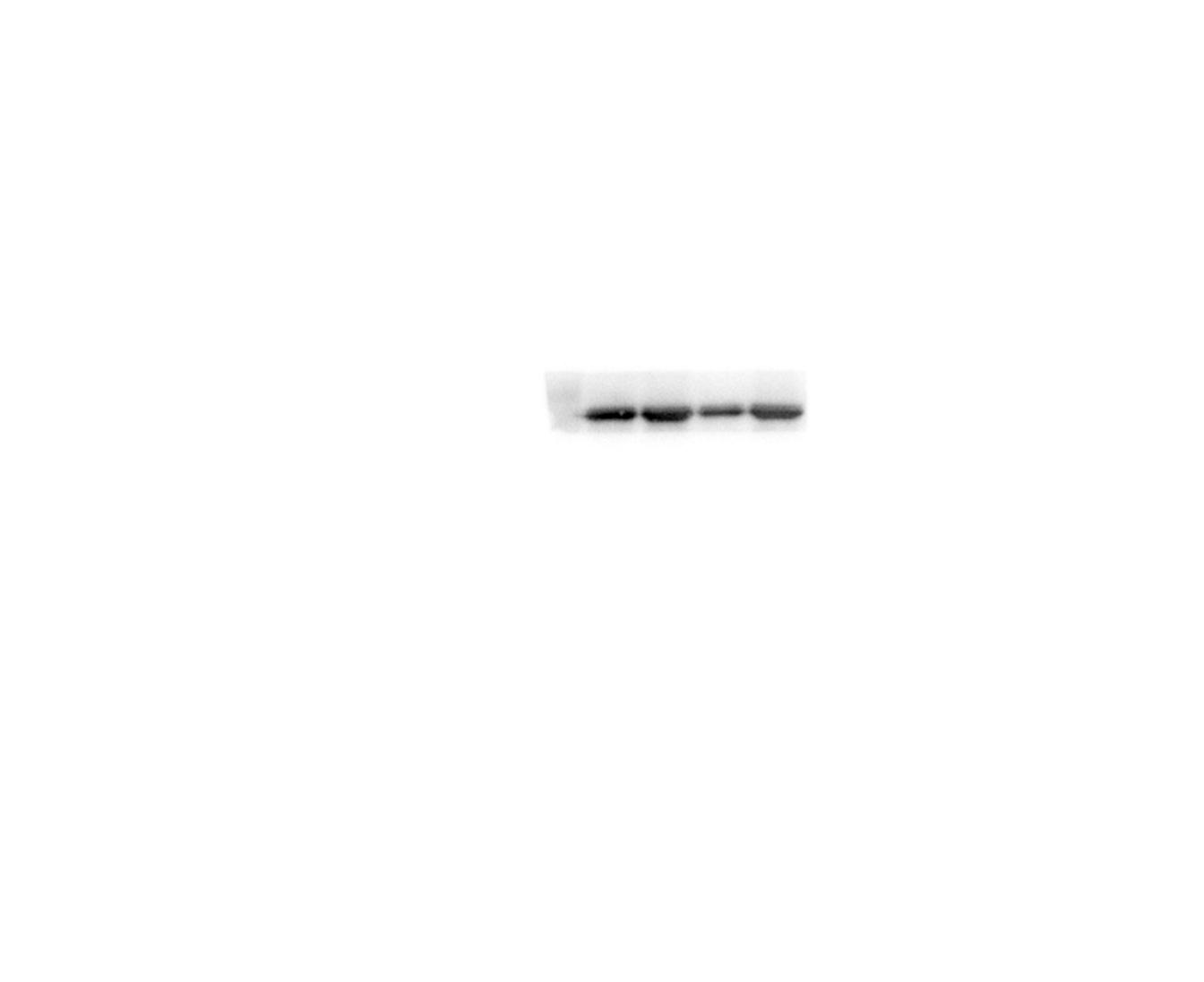** |
|  |  | **ZMAT3** | | **MDM2** | |
|  |  | **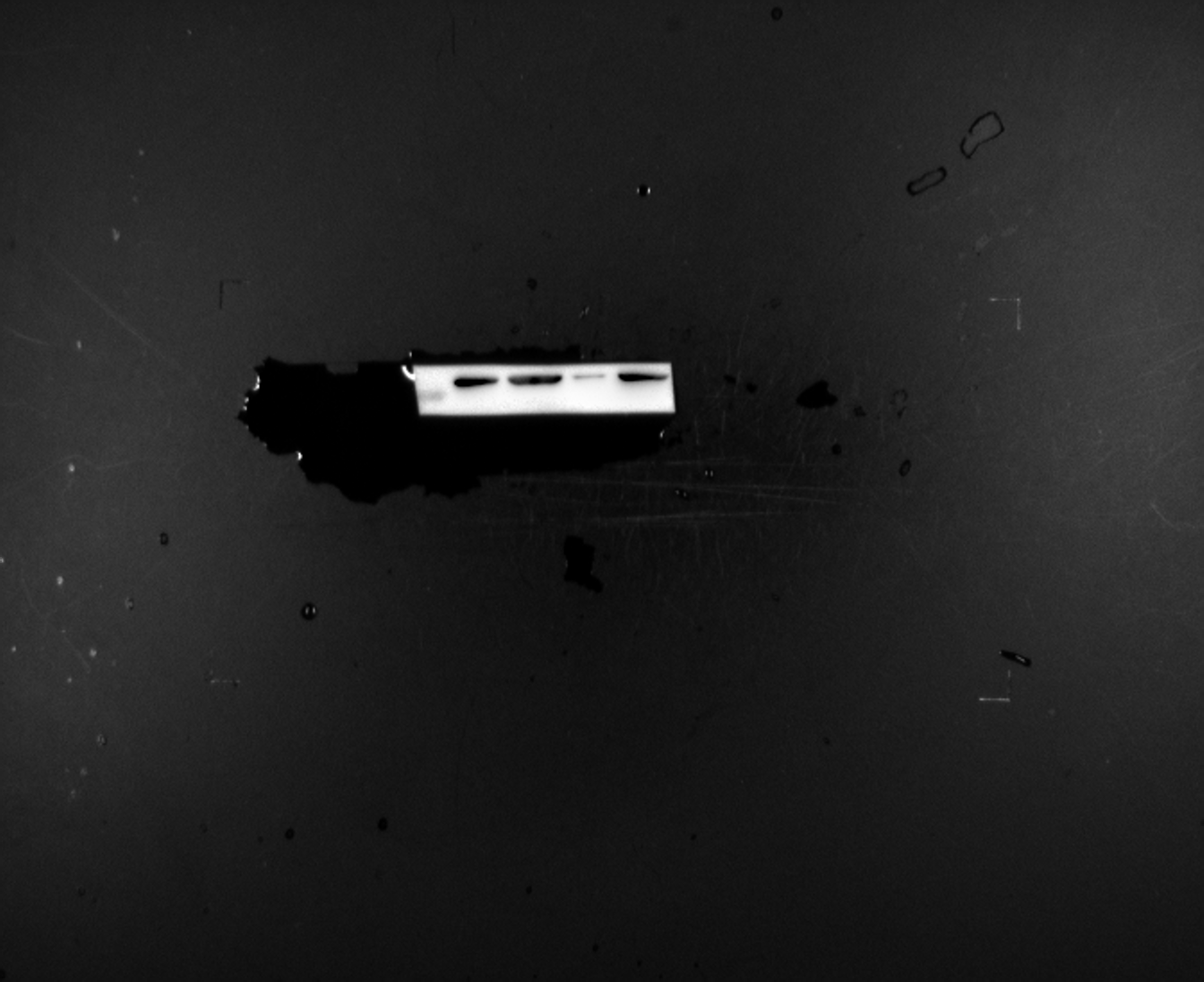** | **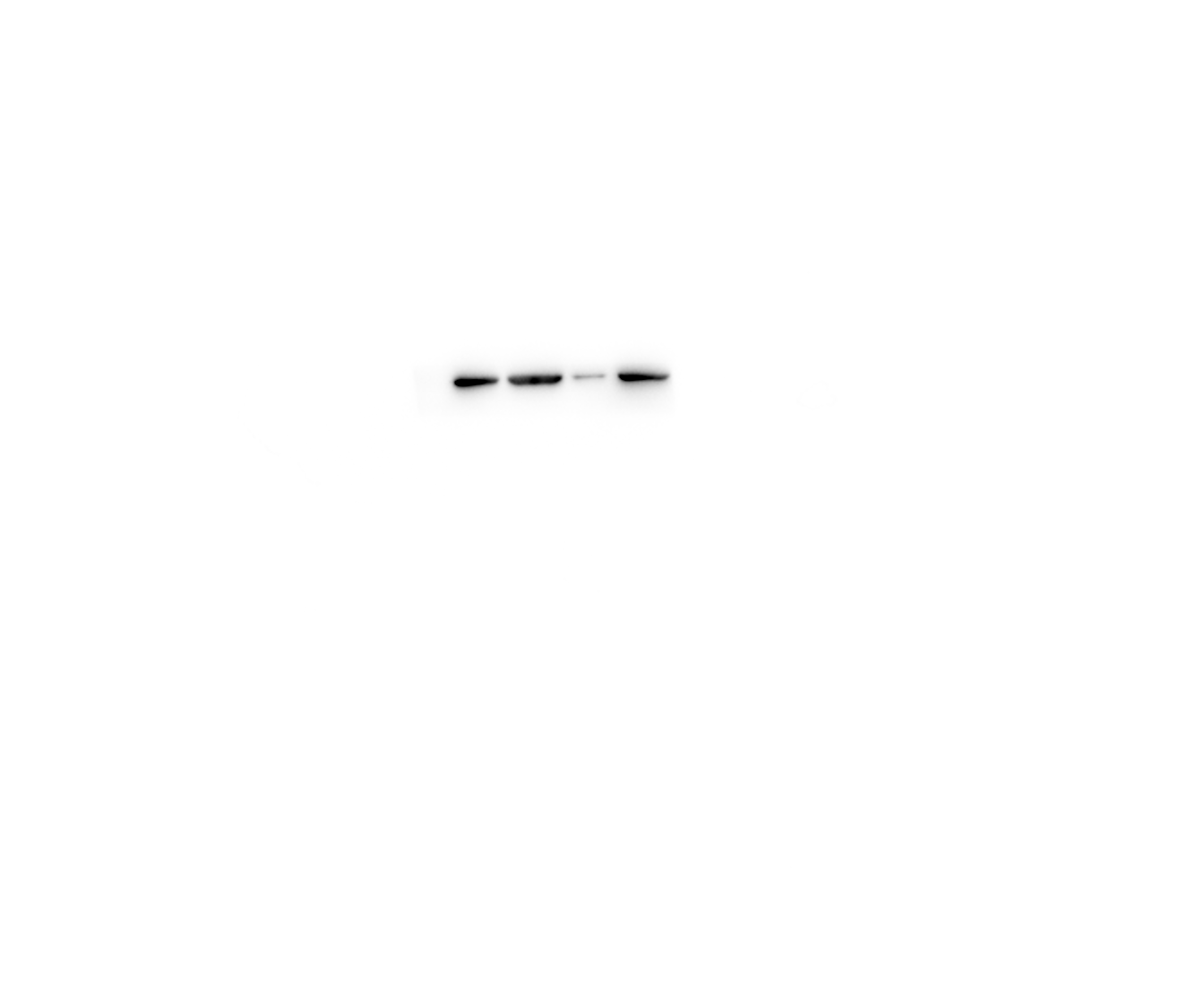** | **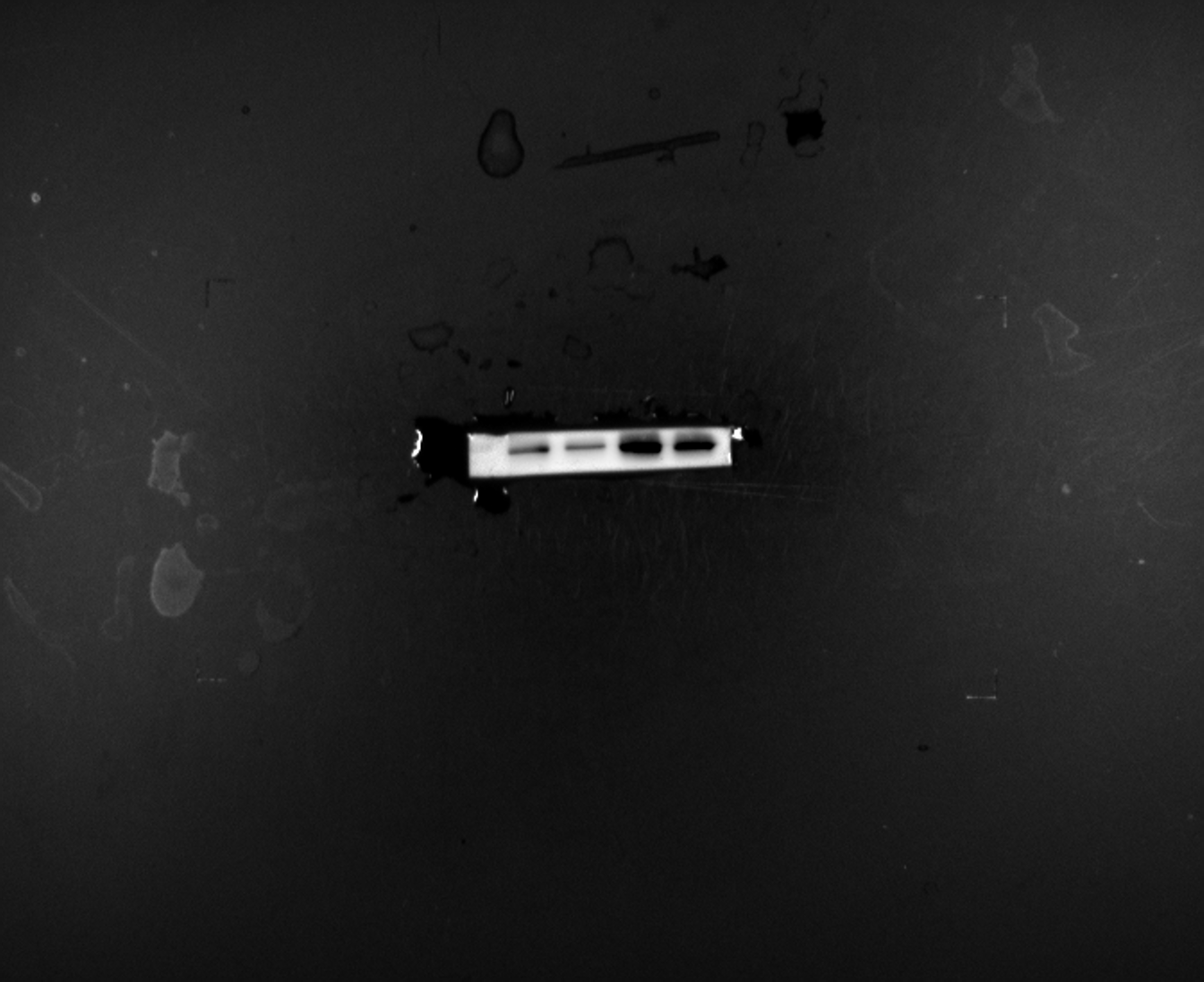** | **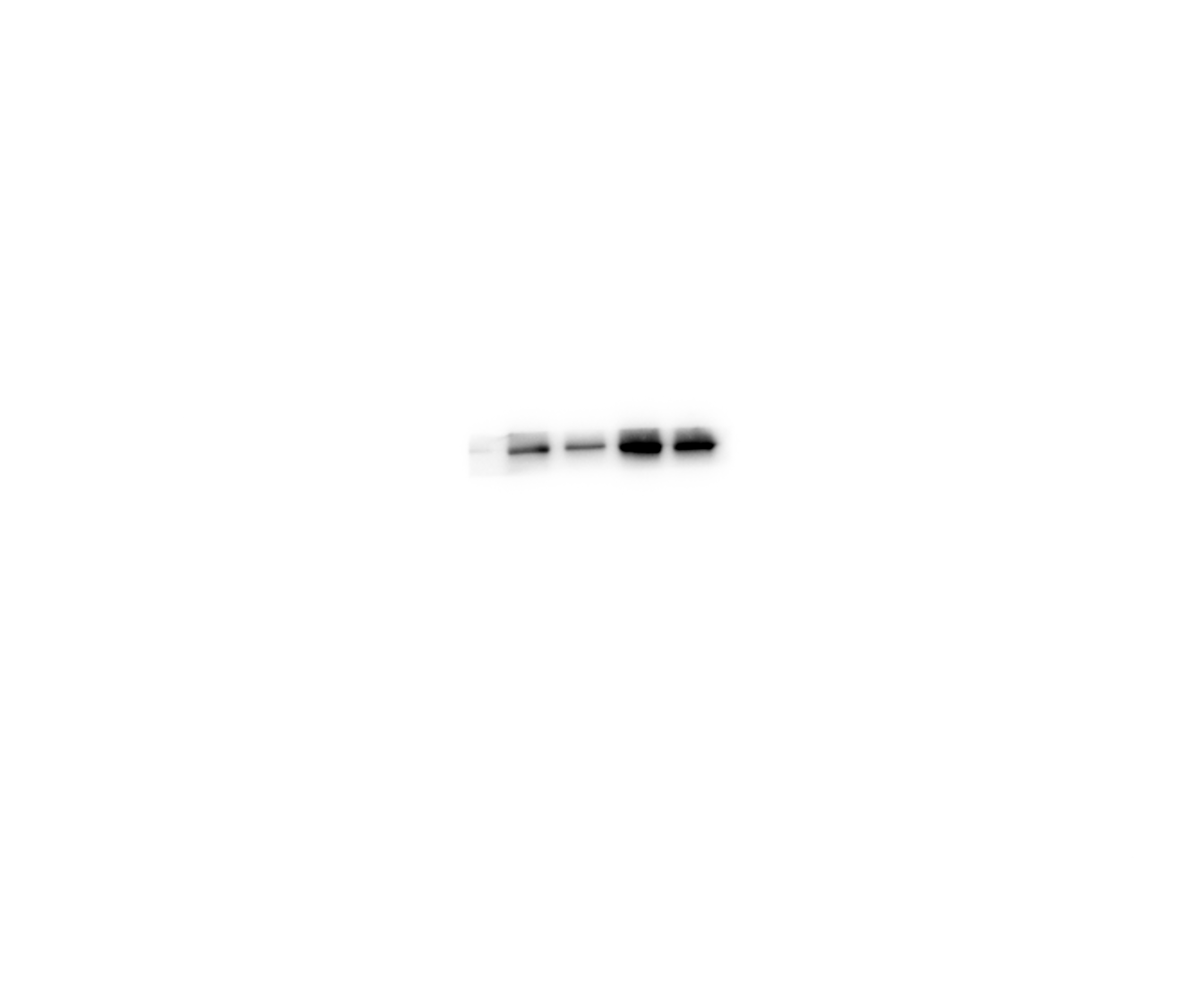** |
|  |  | **SP1** | | **GAPDH** | |
|  |  | **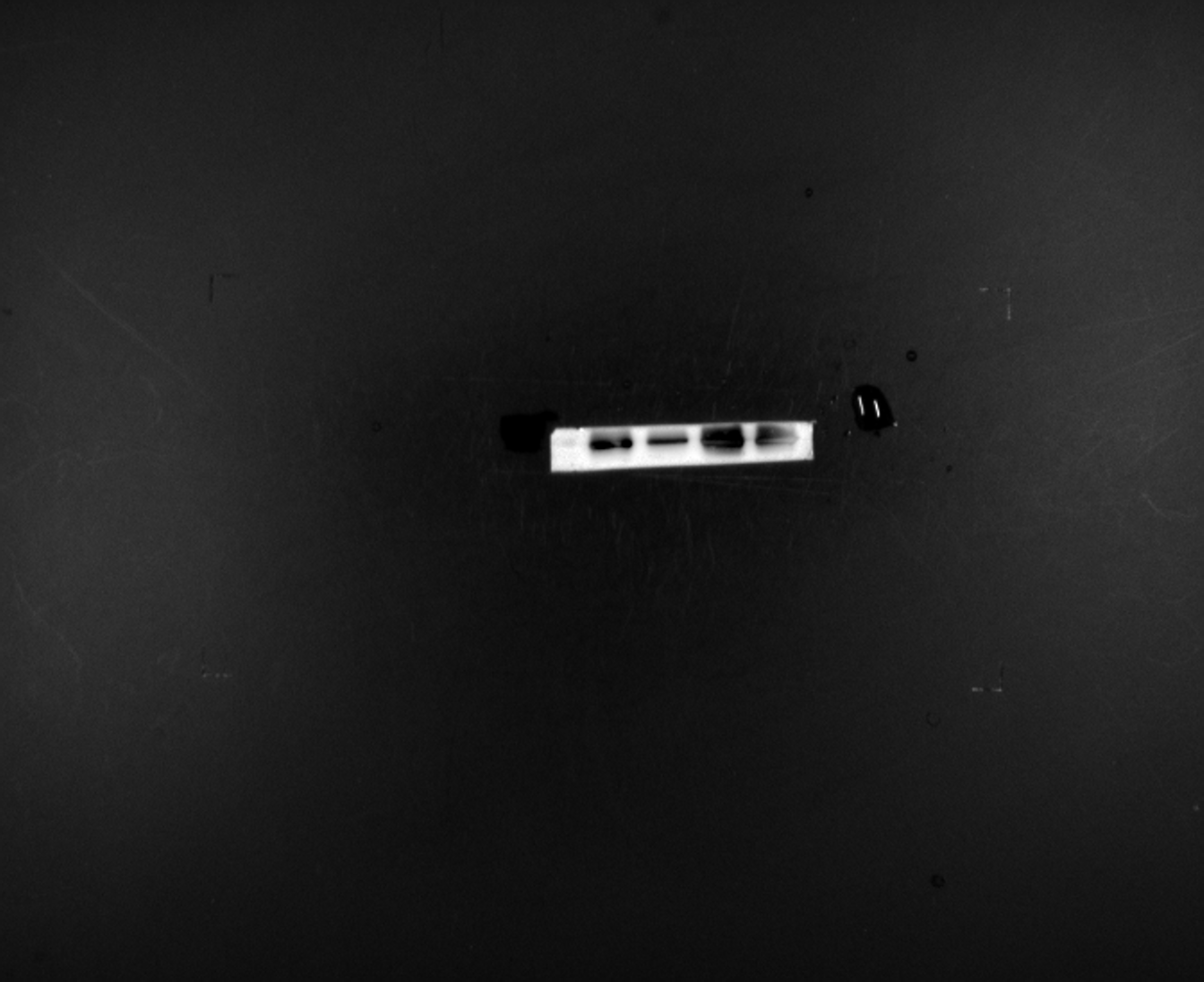** | **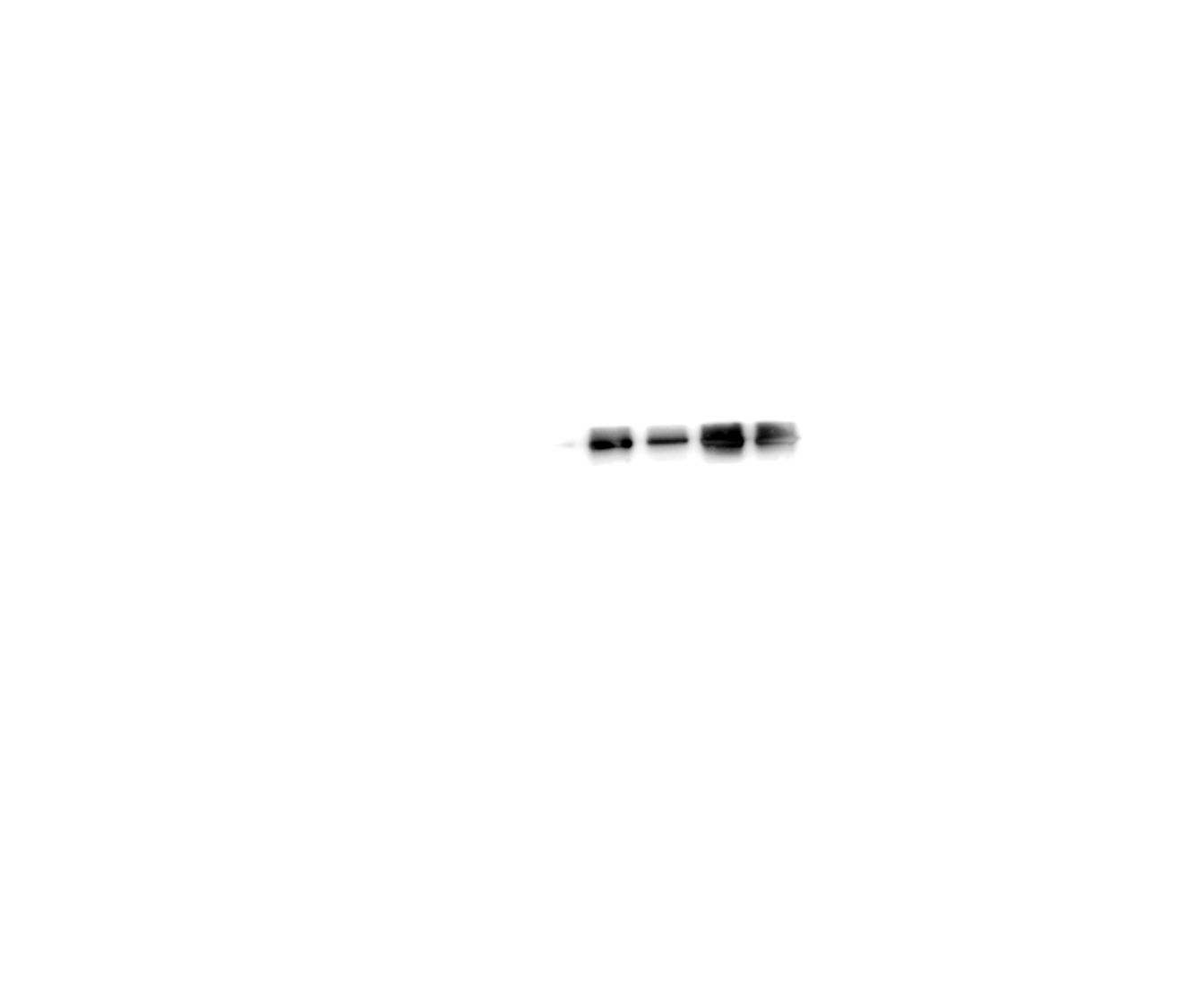** | **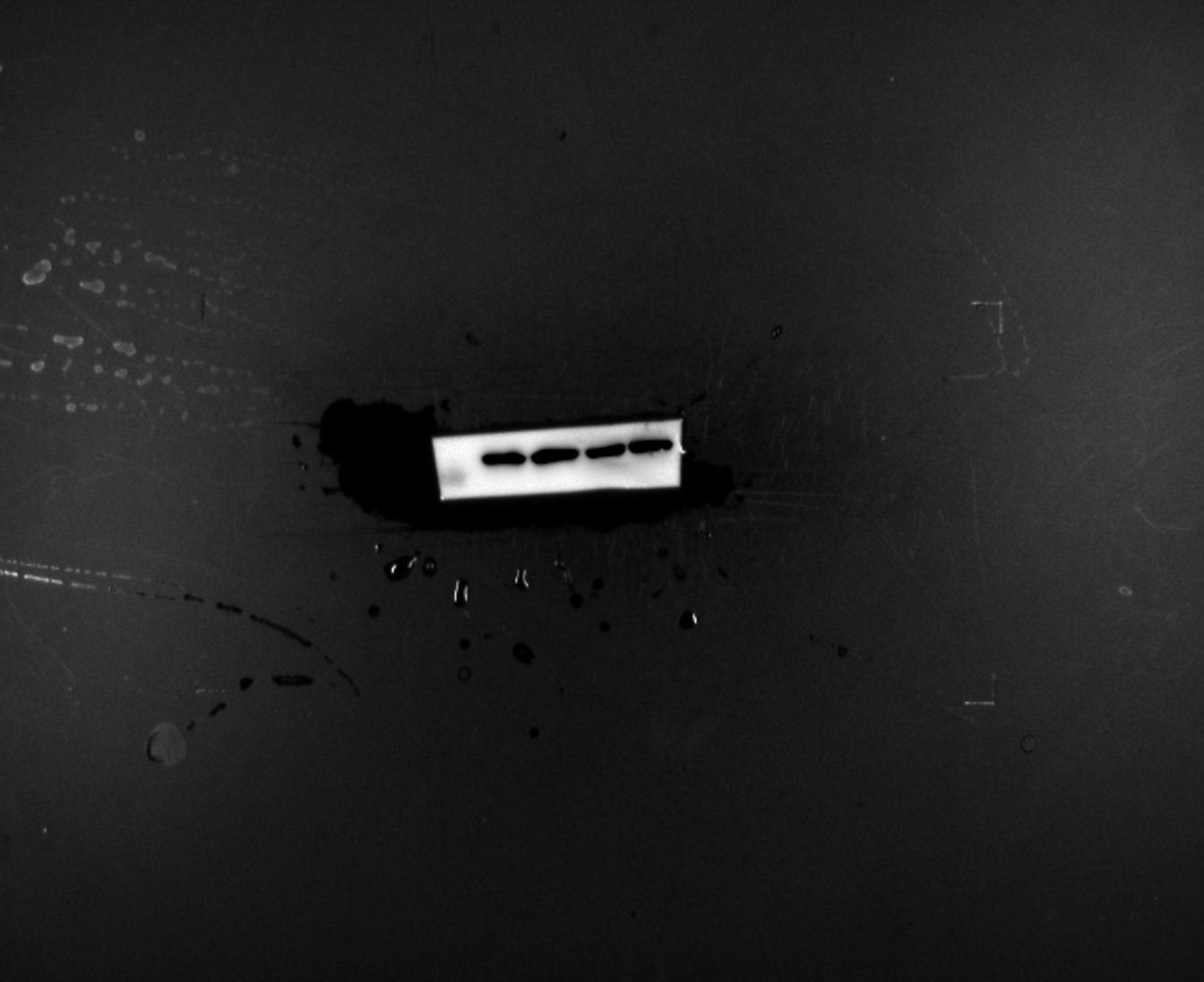** | **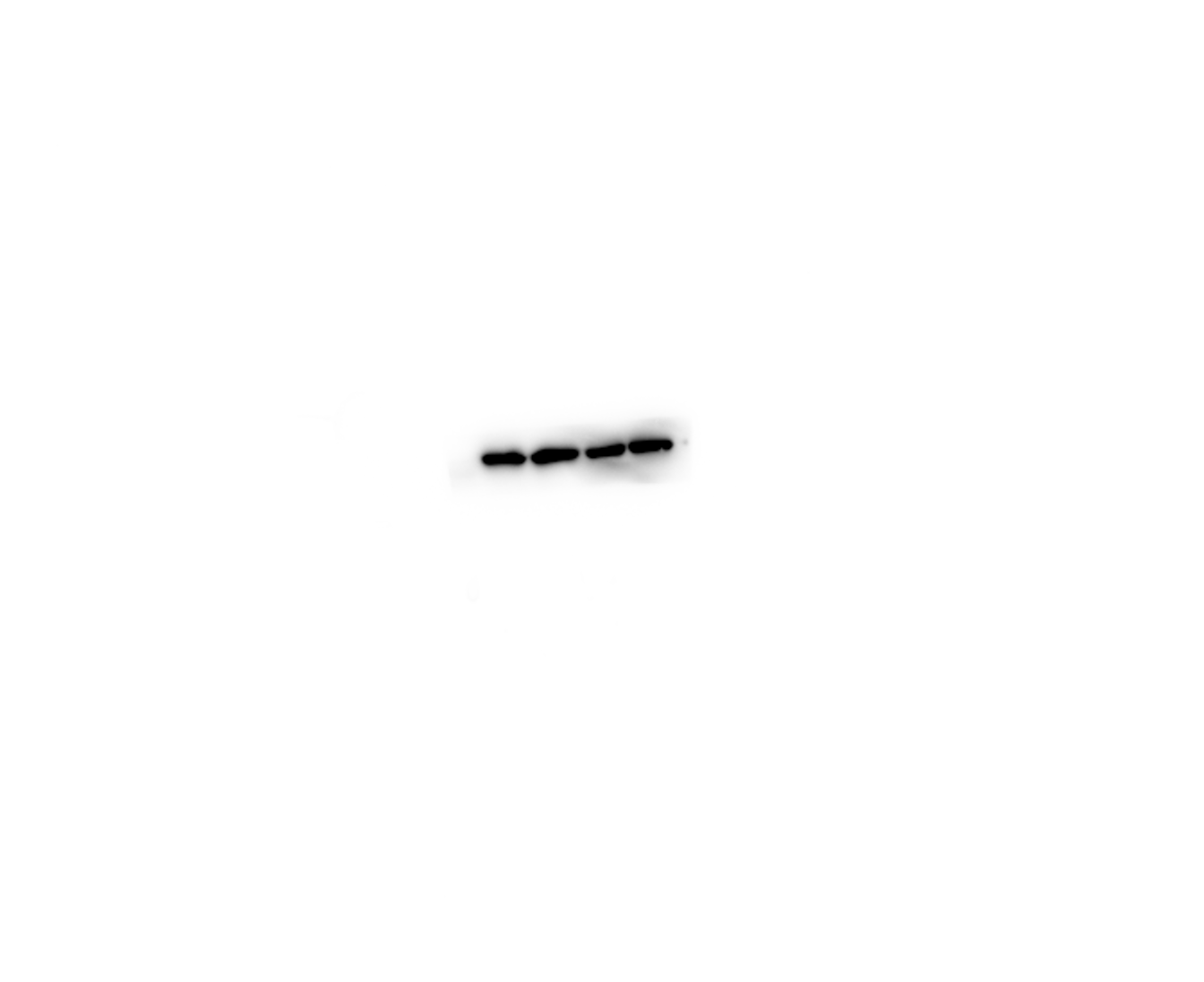** |
| **Fig 5B** | | **p-p53** | | **KLF4** | |
| **HGC-27** | | **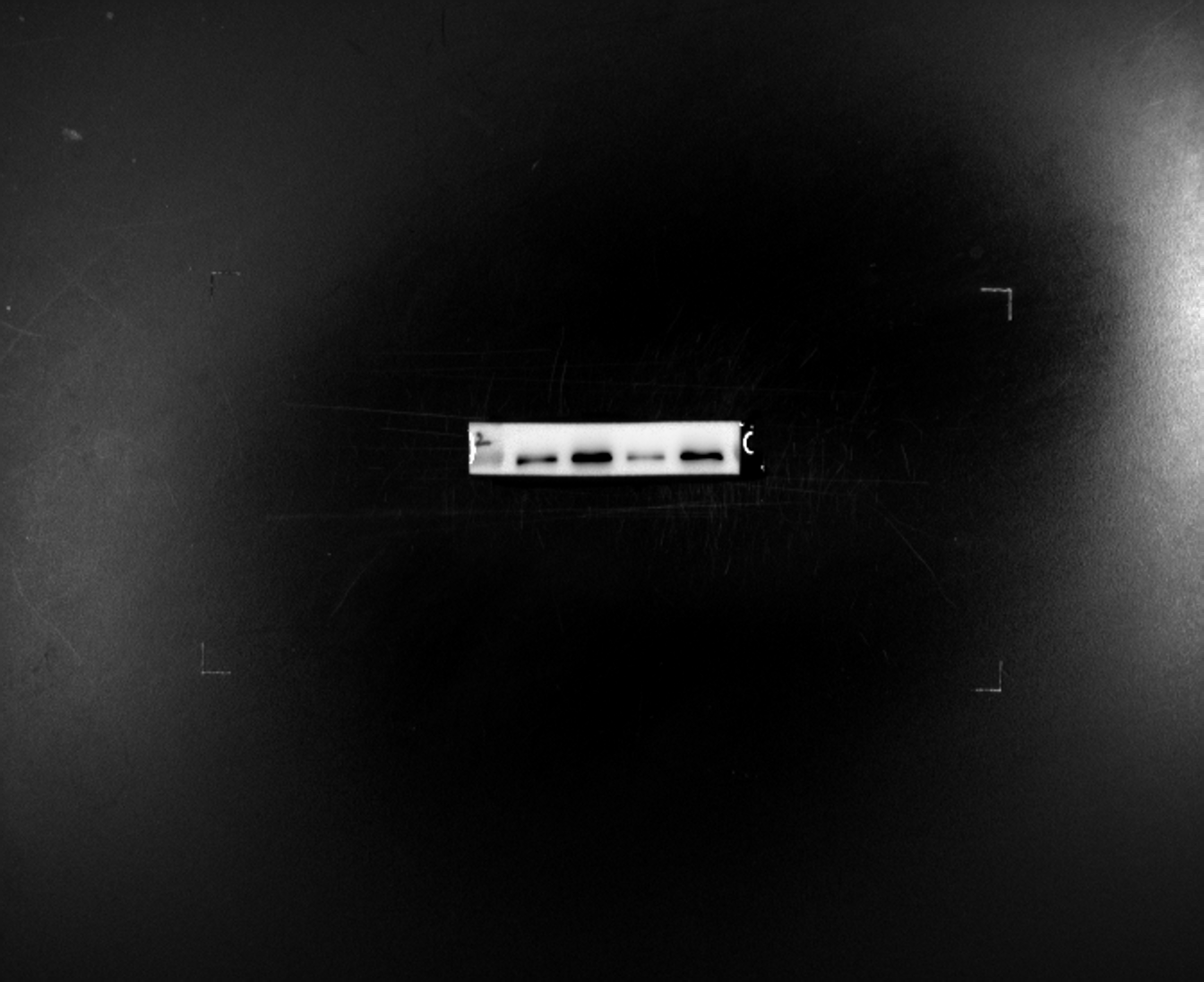** | **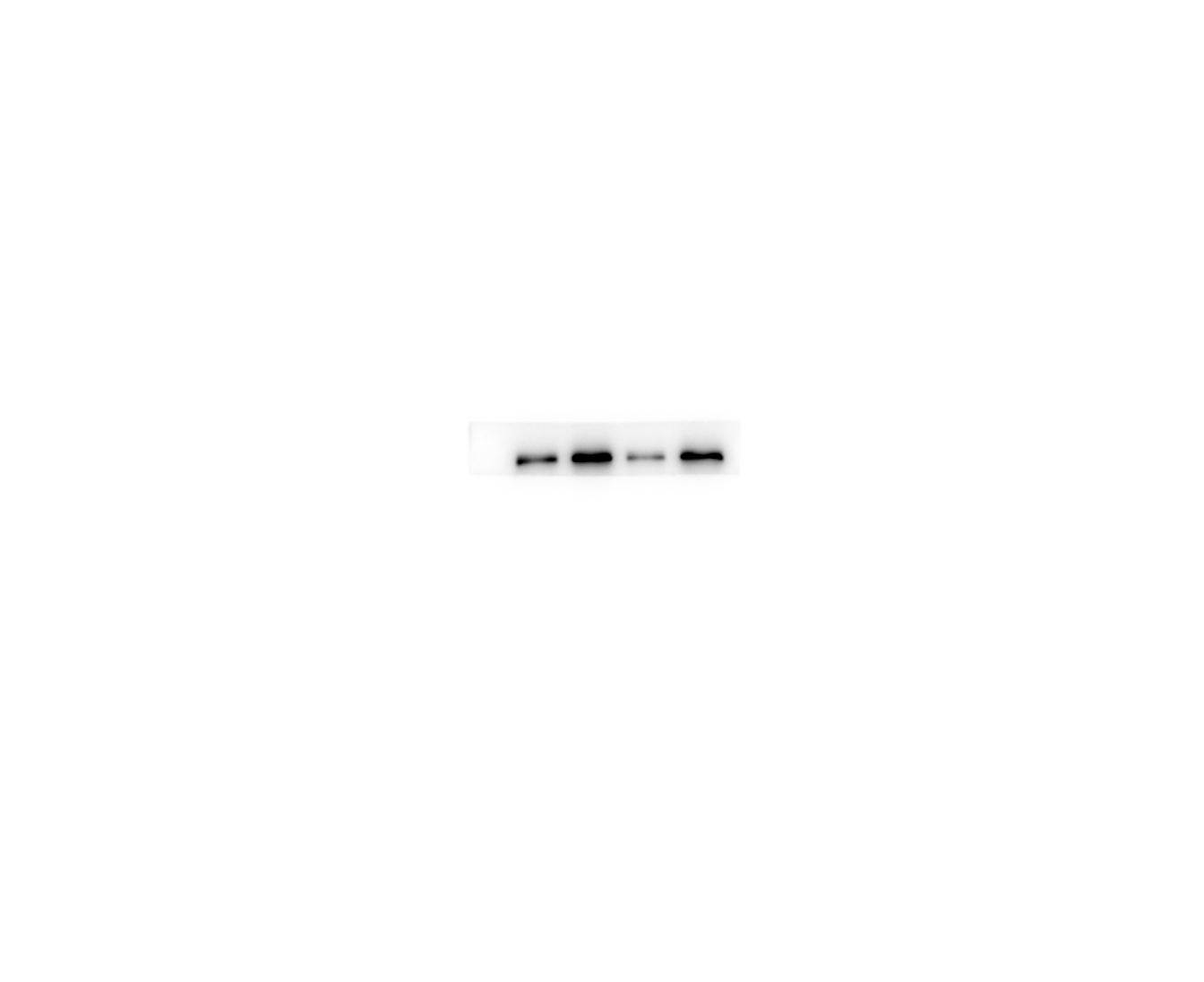** | **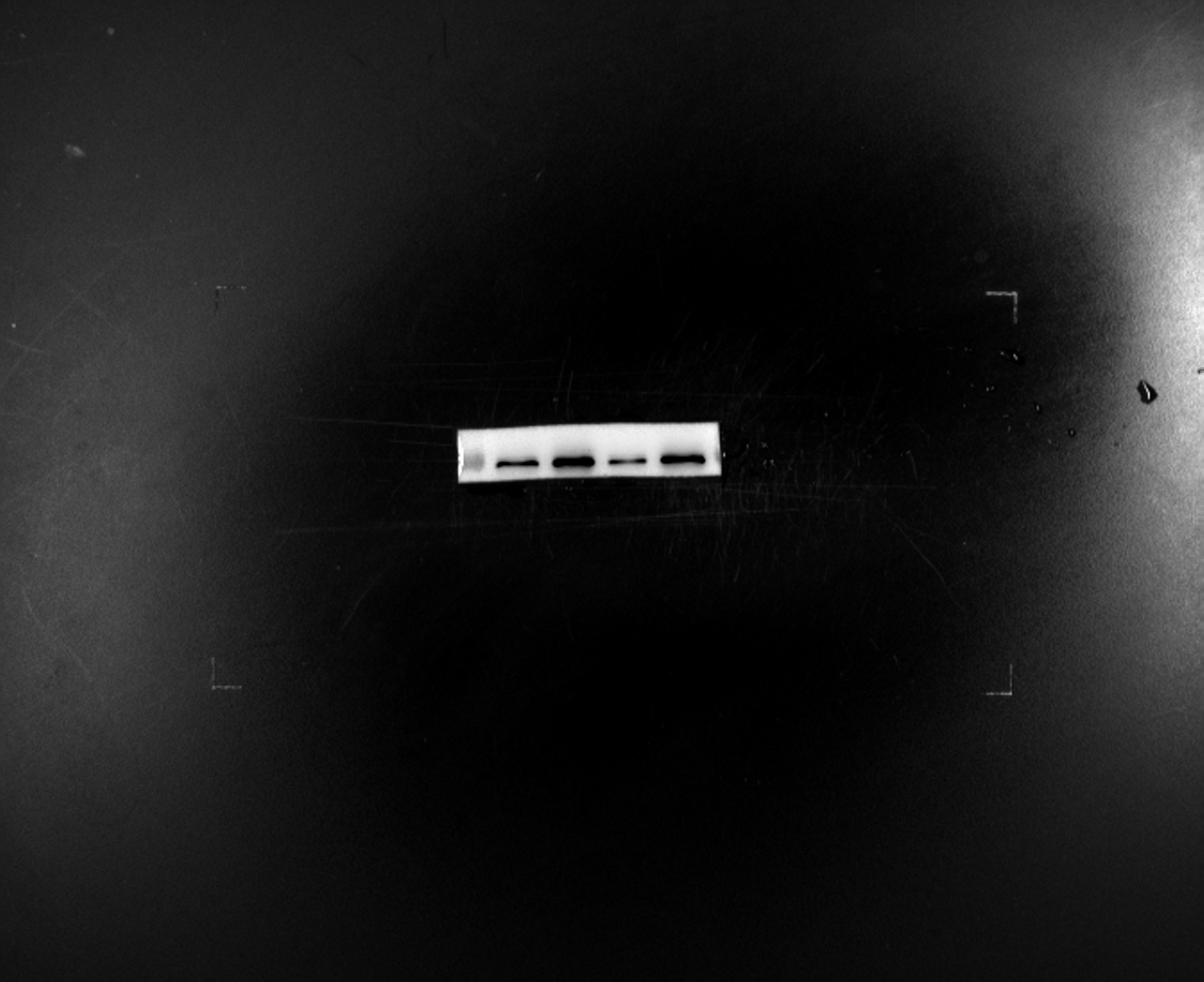** | **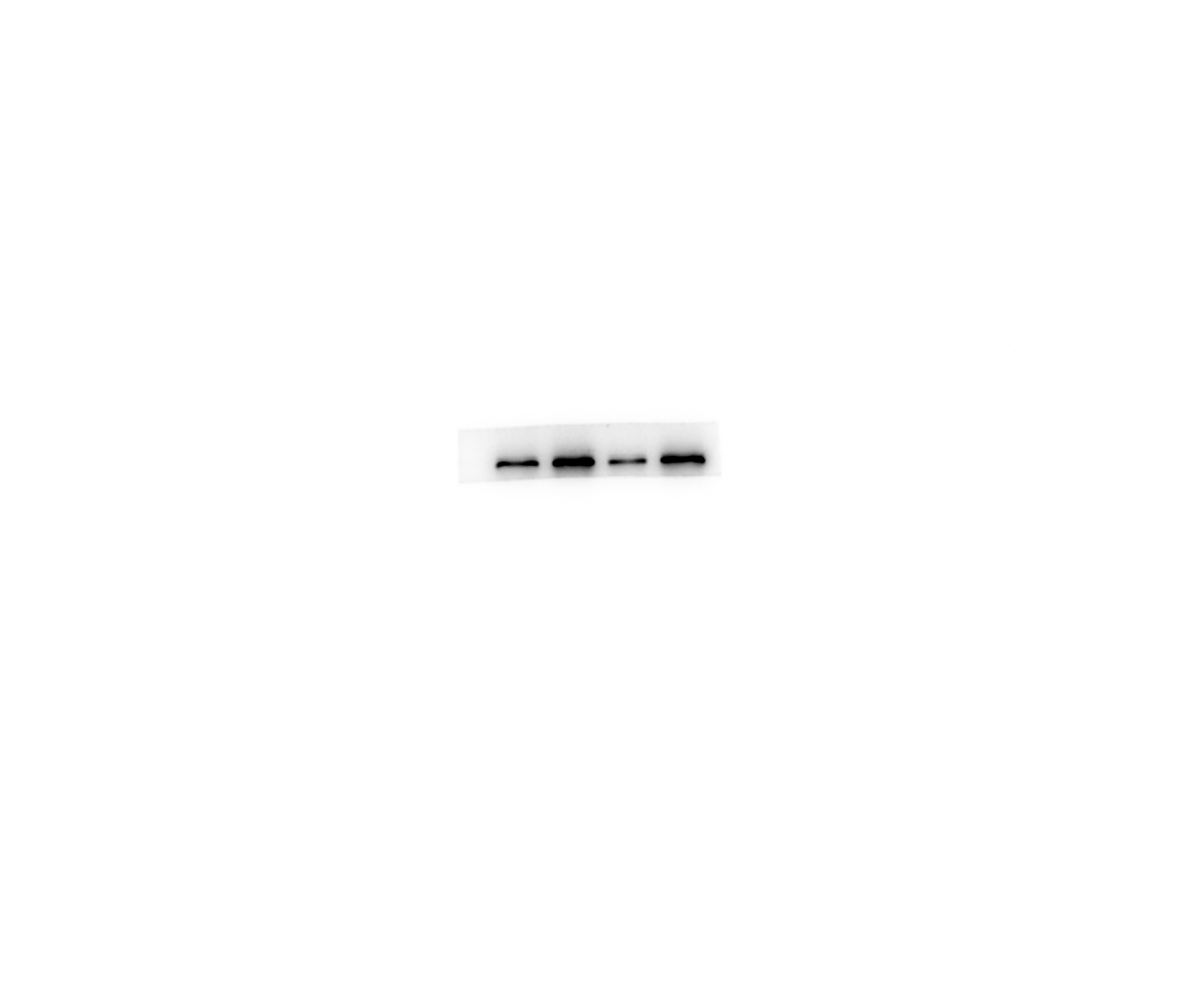** |
|  |  | **ZMAT3** | | **MDM2** | |
|  |  | **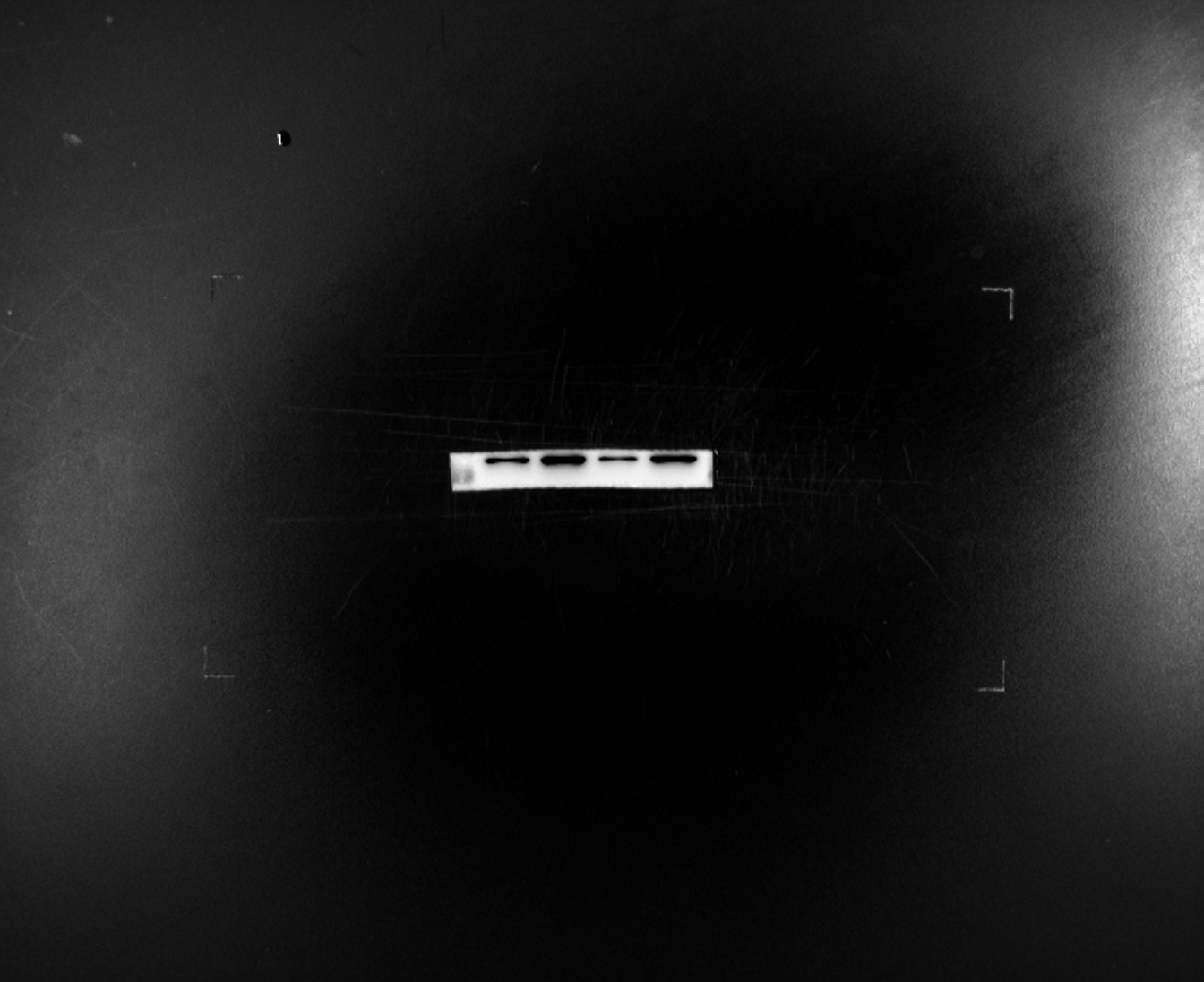** | **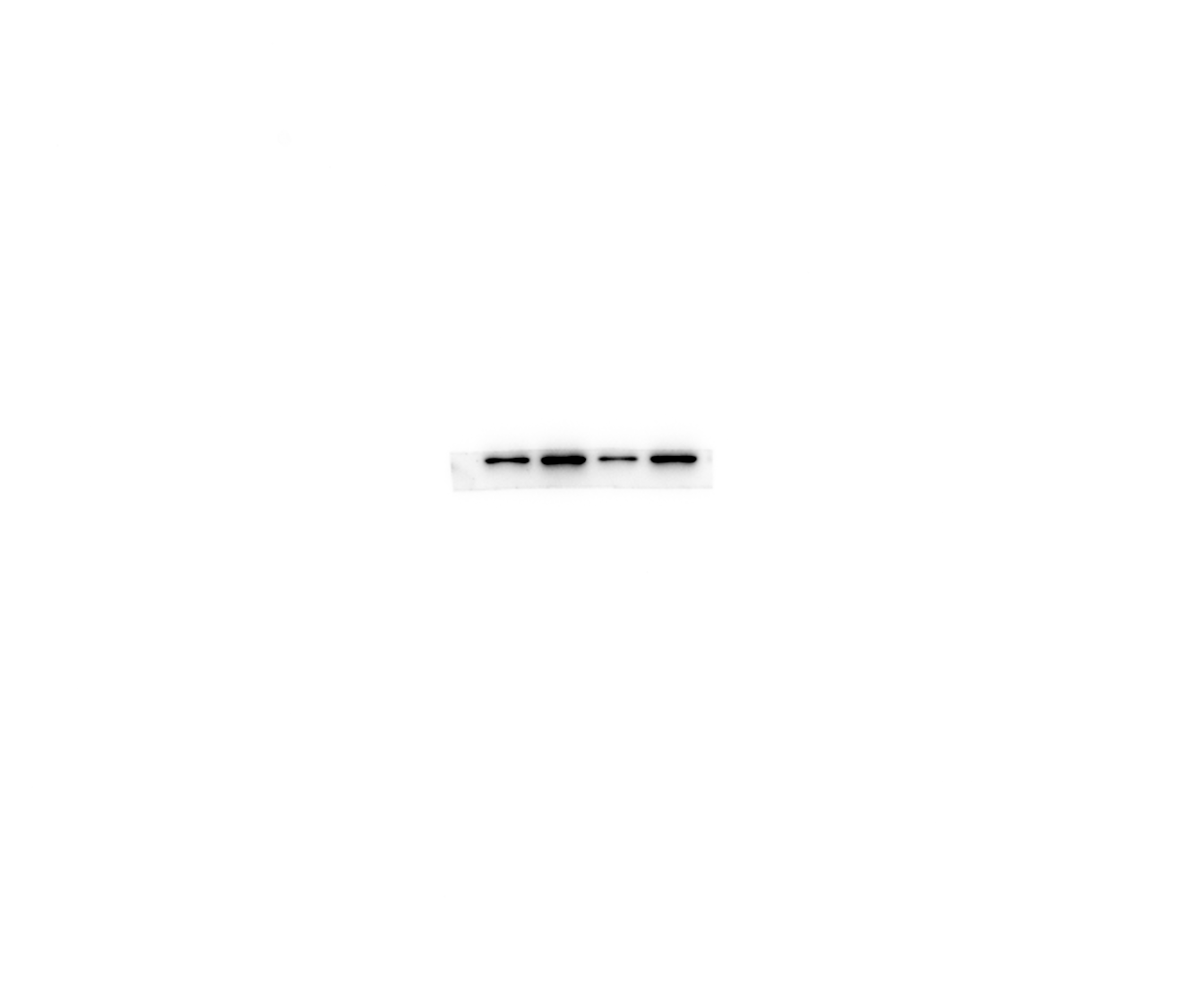** | **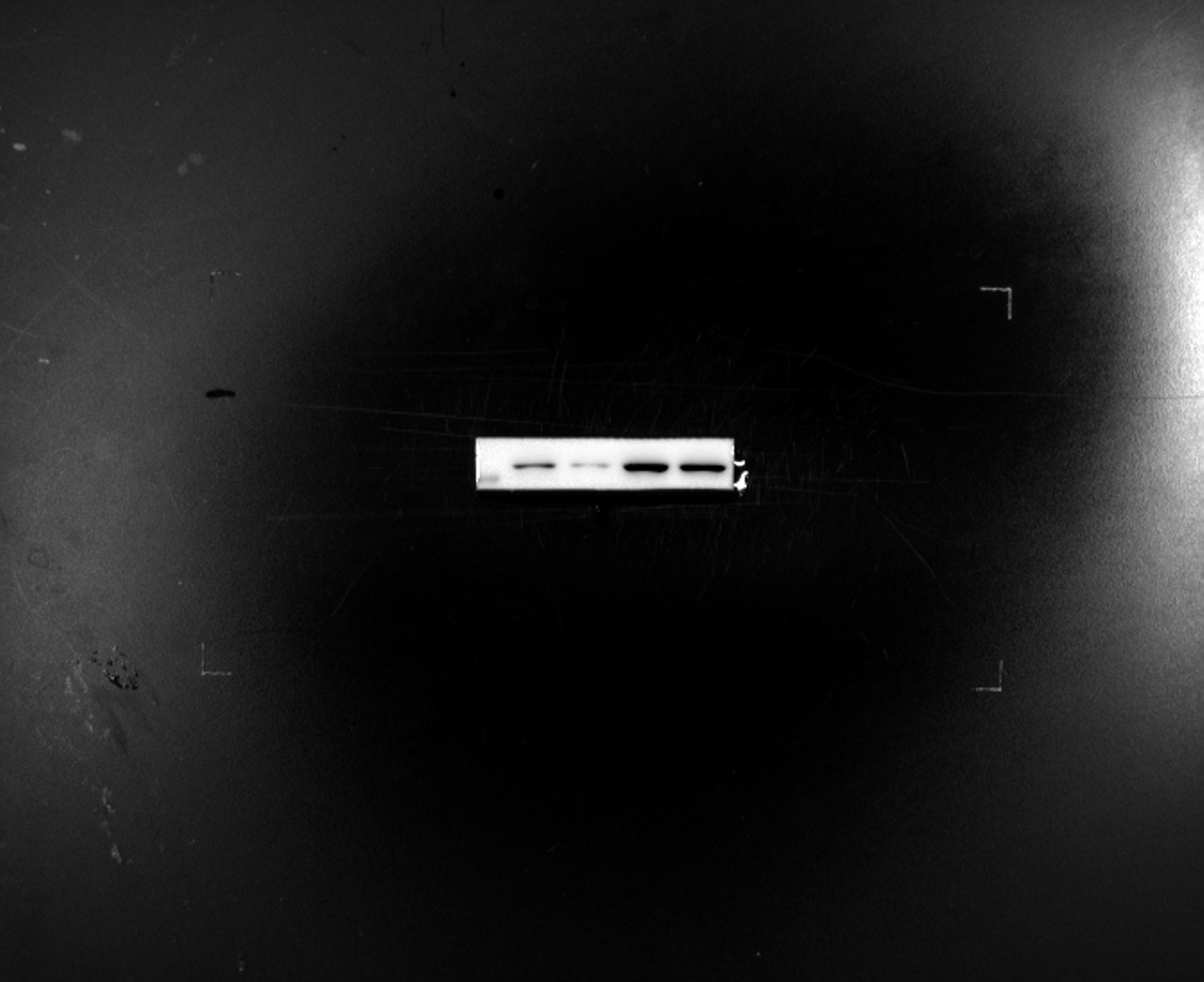** | **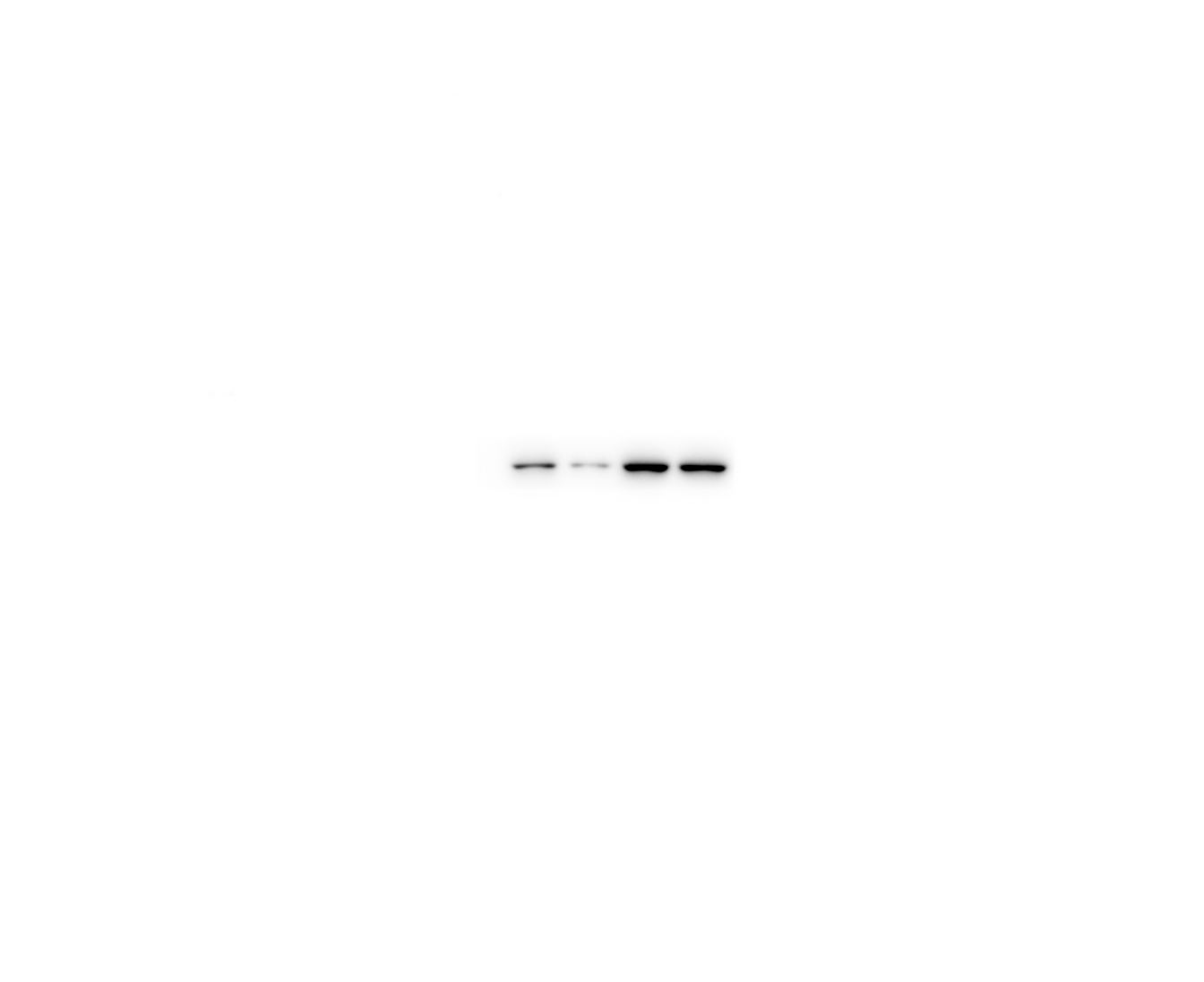** |
|  |  | **SP1** | | **GAPDH** | |
|  |  | **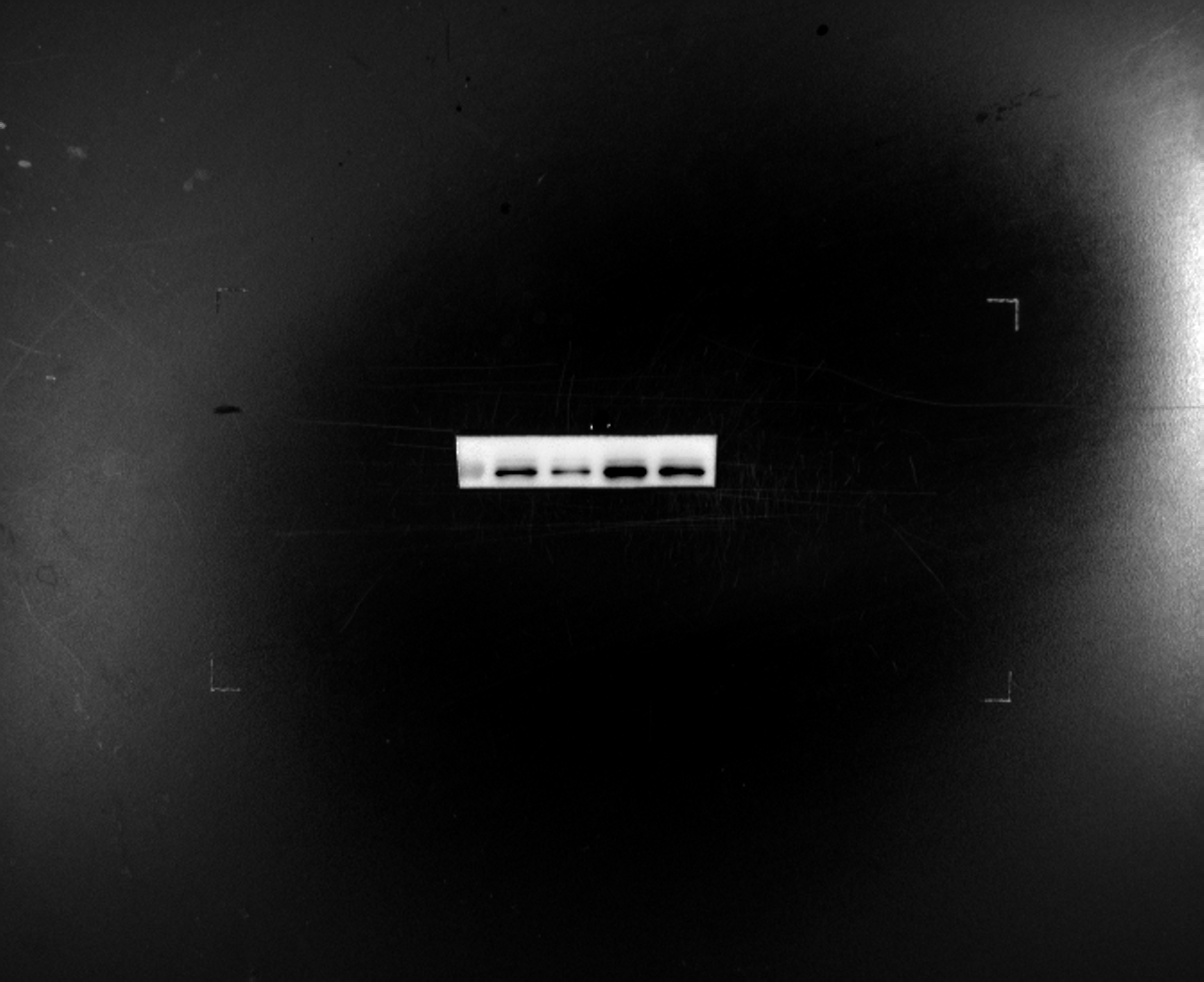** | **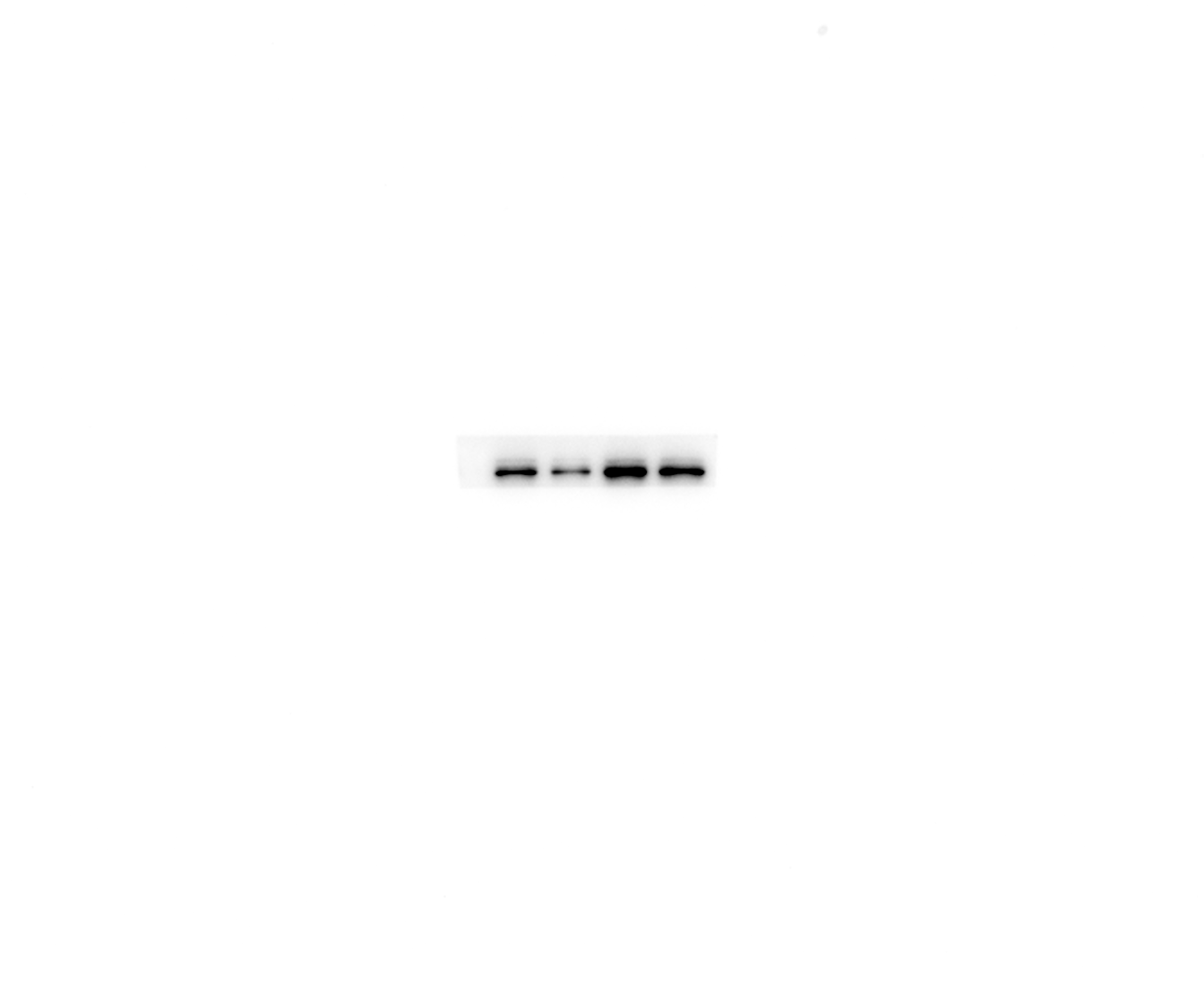** | **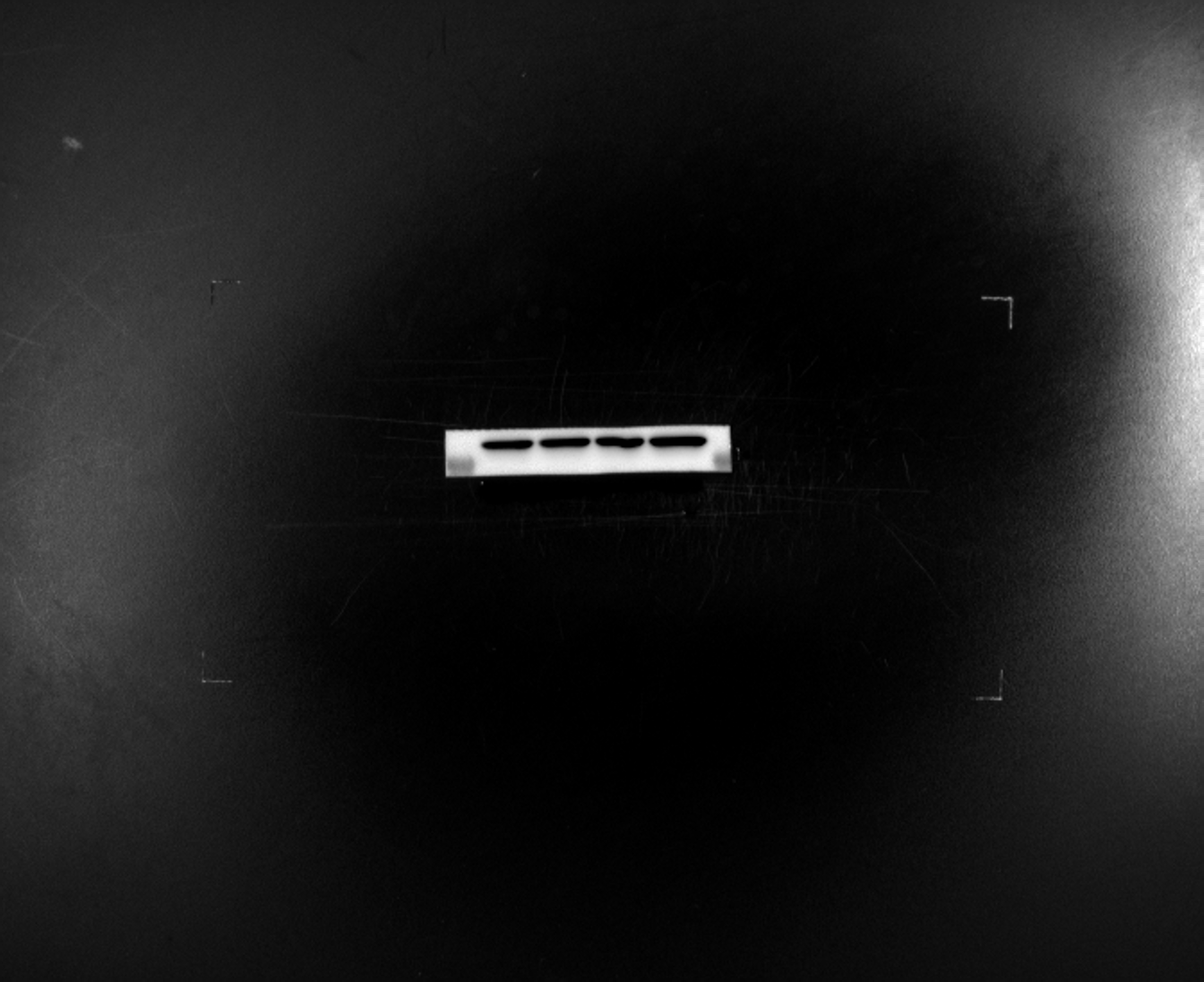** | **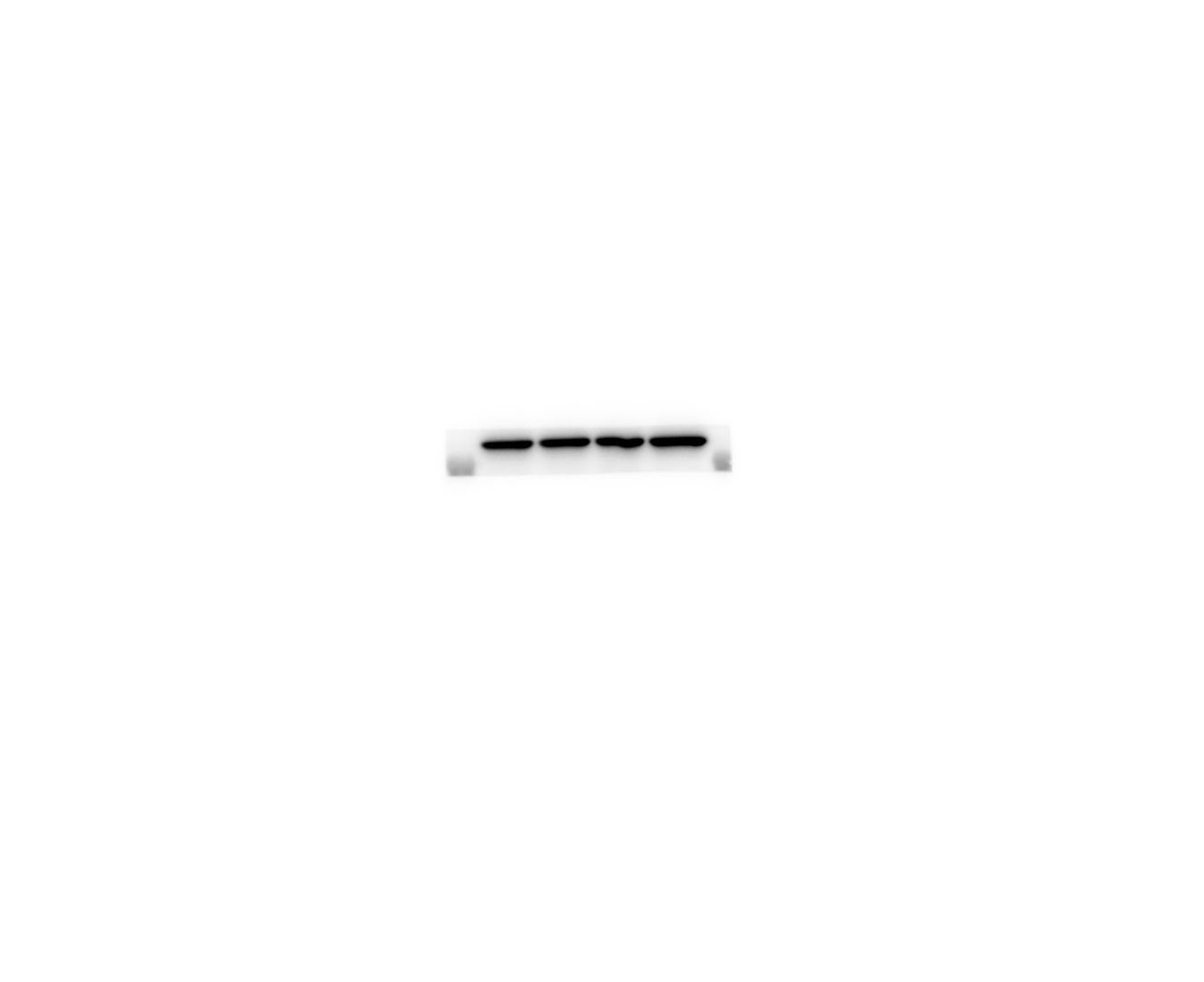** |
| **Fig 6A** | | **p-P53** | | **GAPDH** | |
| **SNU-216** | | **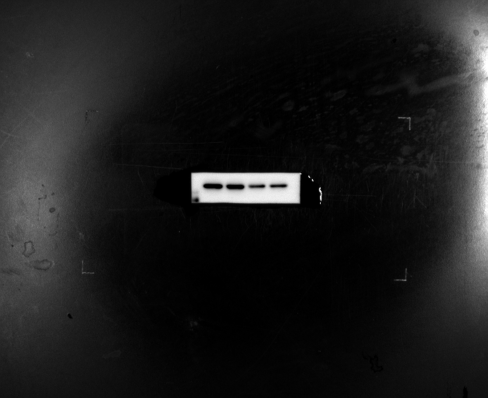** | **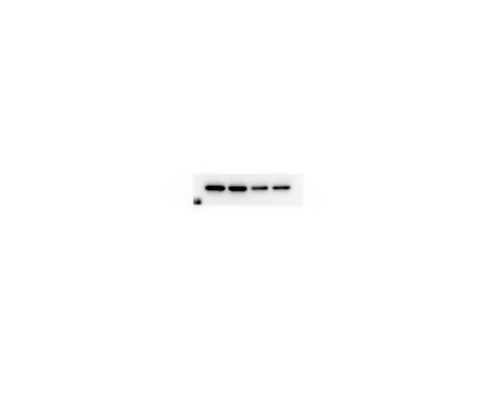** | **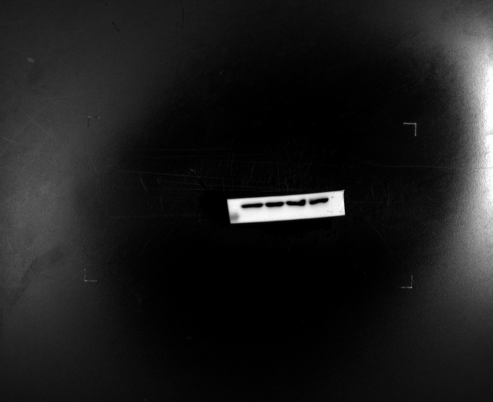** | **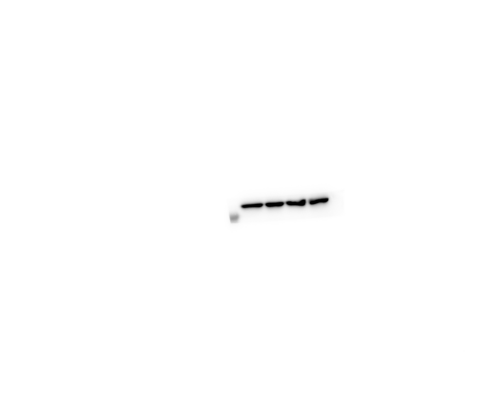** |
| **HGC-27** | | **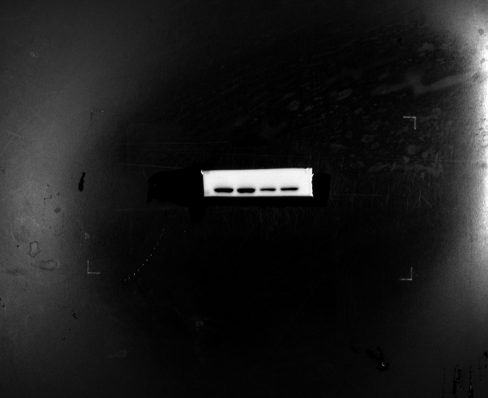** | **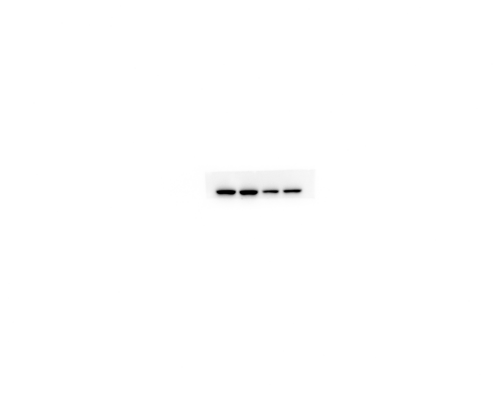** | **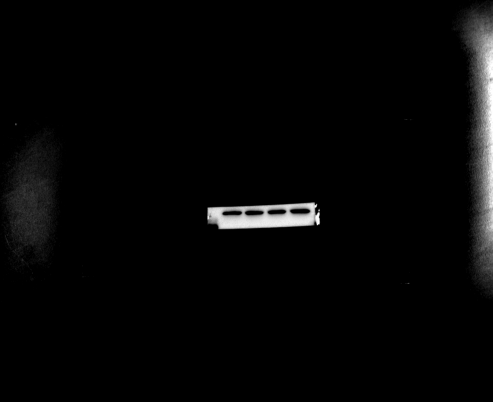** | **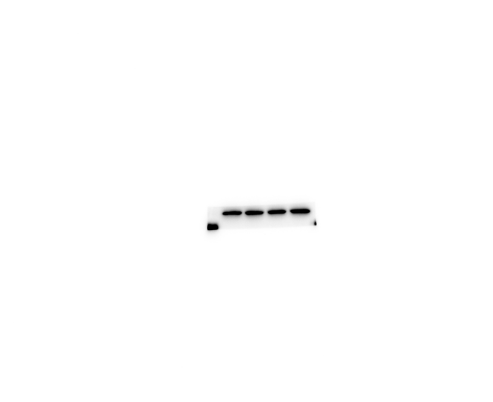** |
| **Fig 6B** | | **p-p53** | | **KLF4** | |
| **SNU-216** | | **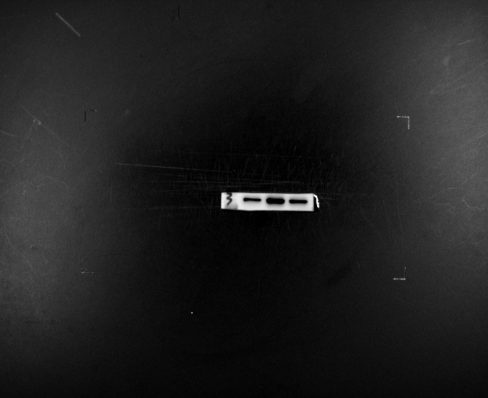** | **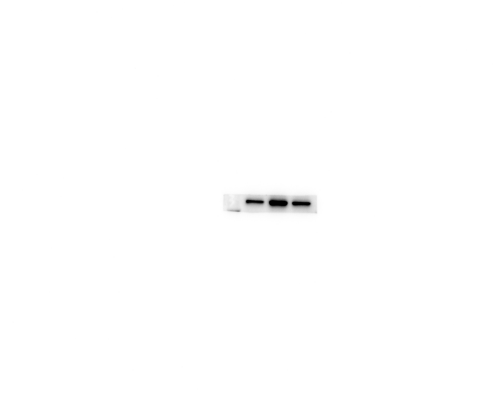** | **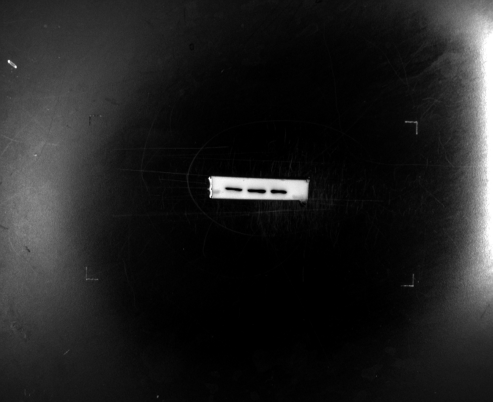** | **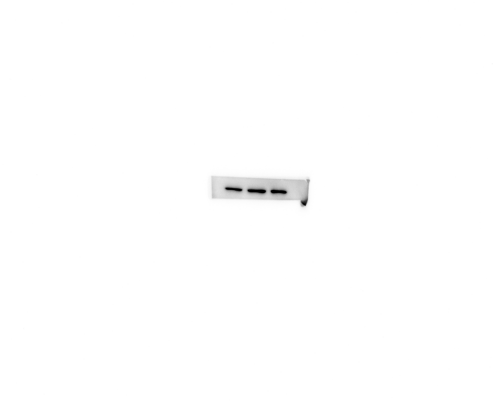** |
|  |  | **ZMAT3** | | **MDM2** | |
|  |  | **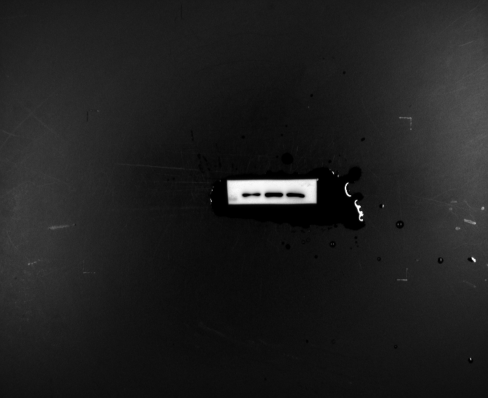** | **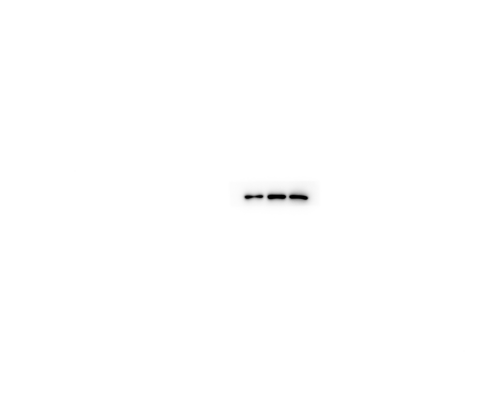** | **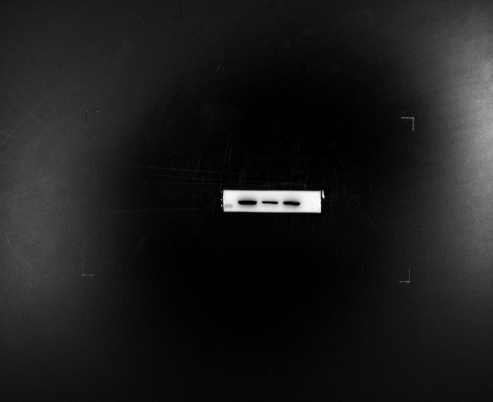** | **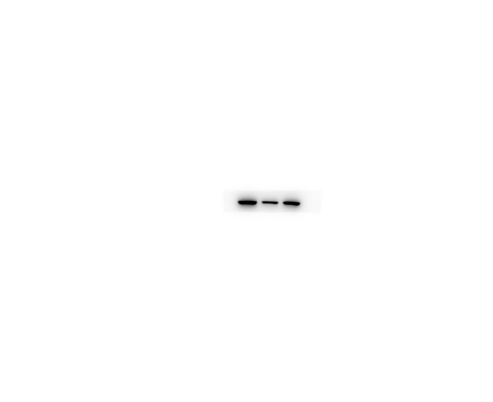** |
|  |  | **SP1** | | **GAPDH** | |
|  |  | **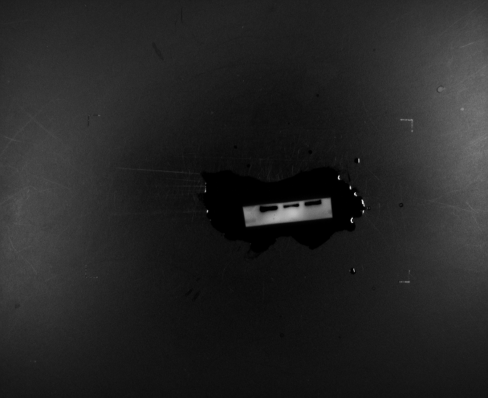** | **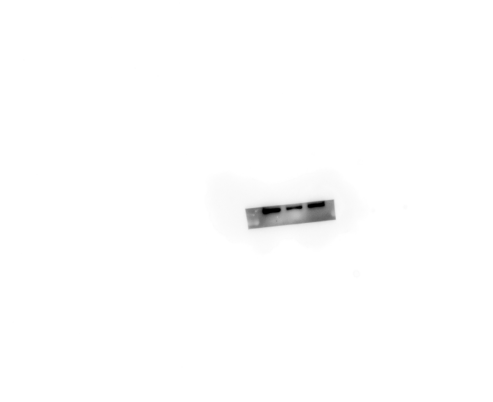** | **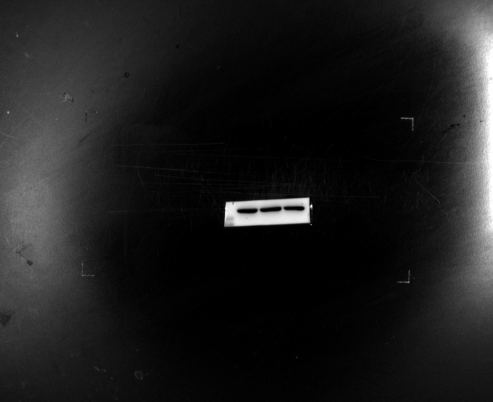** | **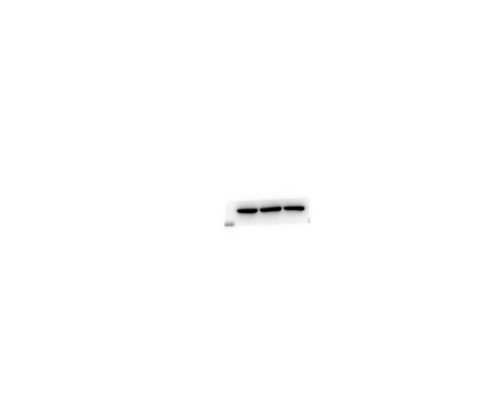** |
| **Fig 6B** | | **p-p53** | | **KLF4** | |
| **HGC-27** | | **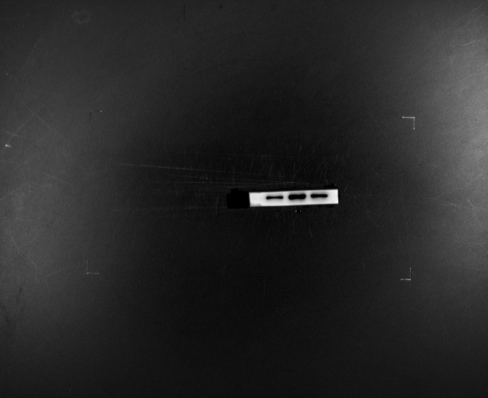** | **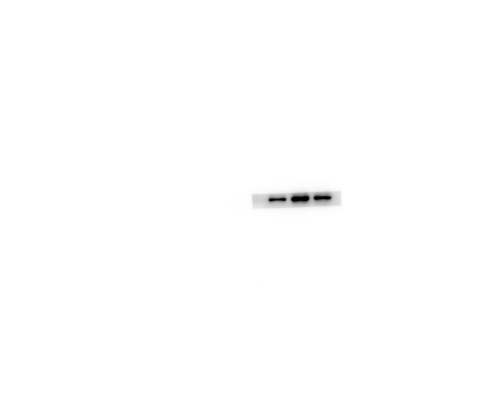** | **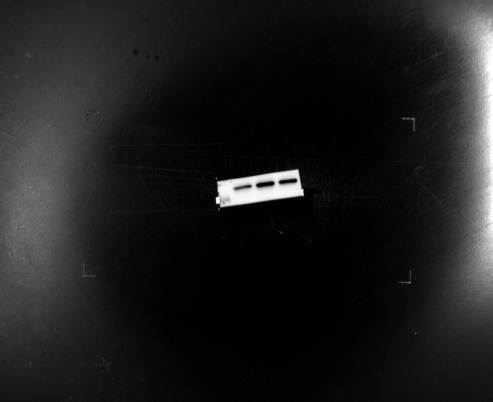** | **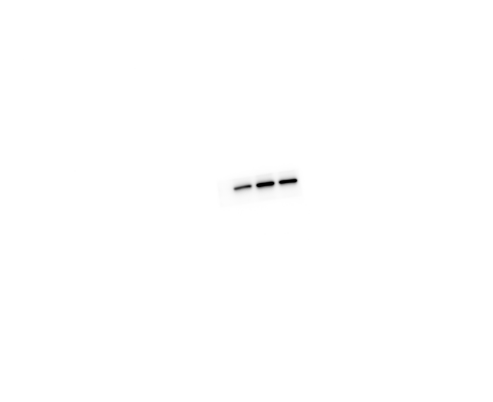** |
|  |  | **ZMAT3** | | **MDM2** | |
|  |  | **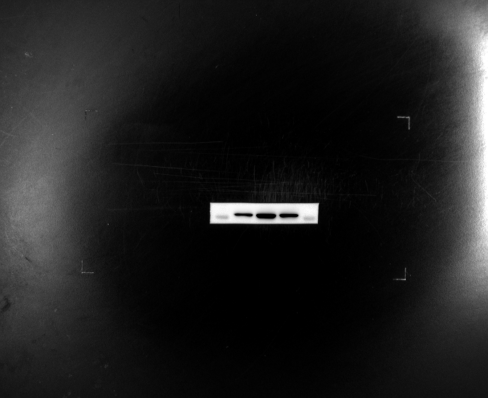** | **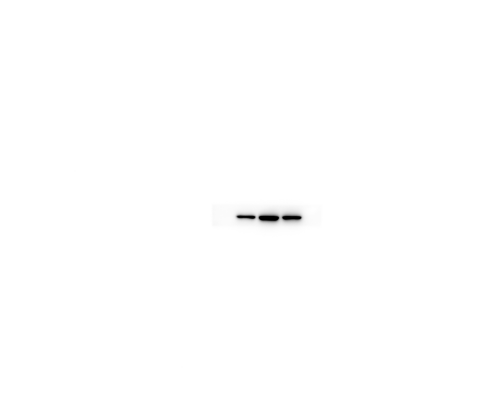** | **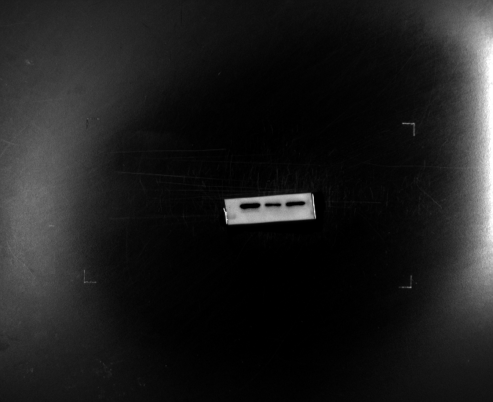** | **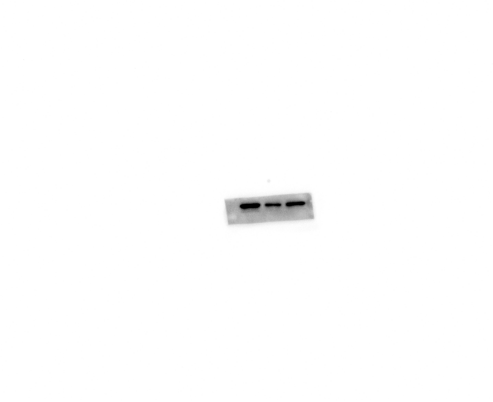** |
|  |  | **SP1** | | **GAPDH** | |
|  |  | **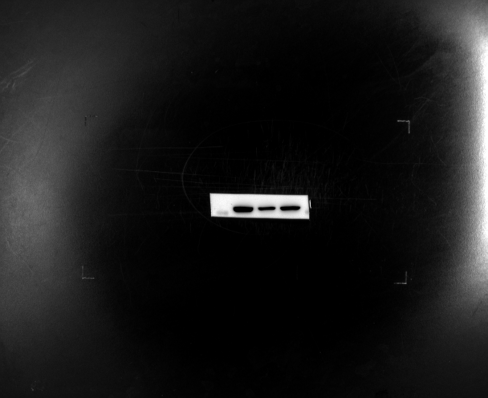** | **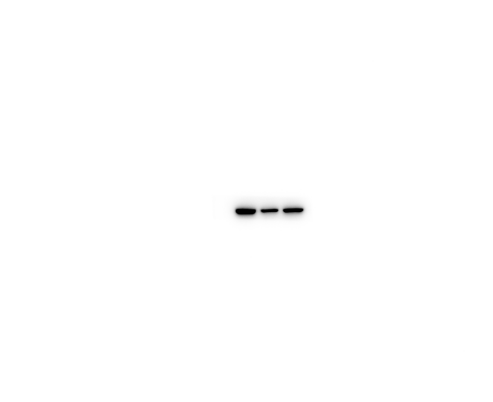** | **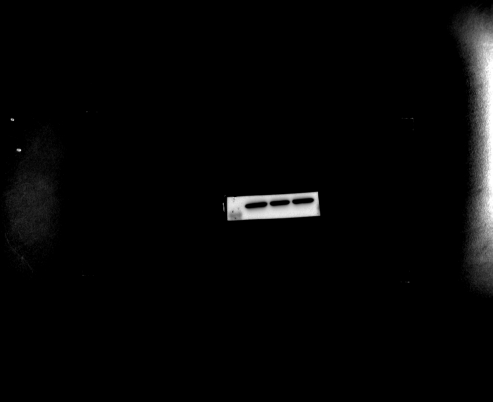** | **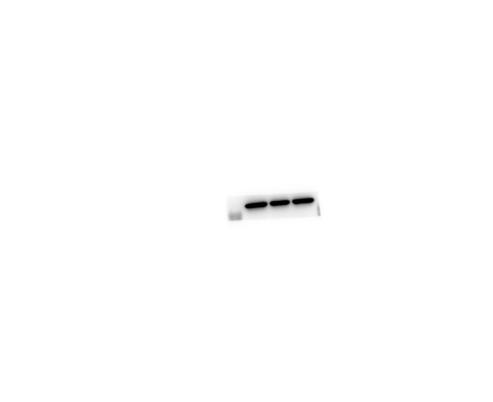** |
